# Transcriptome structure variability in *Saccharomyces cerevisiae* strains determined with a newly developed assembly software

**DOI:** 10.1186/1471-2164-15-1045

**Published:** 2014-12-01

**Authors:** Alessandro Sardu, Laura Treu, Stefano Campanaro

**Affiliations:** Department of Biology, Division of Biochemistry, University of Fribourg, CH-1700 Fribourg, Switzerland; Department of Environmental Engineering, Technical University of Denmark, DK-2800 Kgs Lyngby, Denmark; Department of Biology, University of Padova, Via Ugo Bassi 58/b, 35131 Padova, Italy

**Keywords:** *Saccharomyces cerevisiae*, *Saccharomyces sensu-stricto*, Transcriptome assembly software, Transcriptome variability, UTR, Non-coding RNA, Cell wall, Reproductive process in single-celled organism

## Abstract

**Background:**

RNA-seq studies have an important role for both large-scale analysis of gene expression and for transcriptome reconstruction. However, the lack of software specifically developed for the analysis of the transcriptome structure in lower eukaryotes, has so far limited the comparative studies among different species and strains.

**Results:**

In order to fill this gap, an innovative software called ORA (Overlapped Reads Assembler) was developed. This software allows a simple and reliable analysis of the transcriptome structure in organisms with a low number of introns. It can also determine the size and the position of the untranslated regions (UTR) and of polycistronic transcripts. As a case study, we analyzed the transcriptional landscape of six *S. cerevisiae* strains in two different key steps of the fermentation process. This comparative analysis revealed differences in the UTR regions of transcripts. By extending the transcriptome analysis to yeast species belonging to the *Saccharomyces* genus, it was possible to examine the conservation level of unknown non-coding RNAs and their putative functional role.

**Conclusions:**

By comparing the results obtained using ORA with previous studies and with the transcriptome structure determined with other software, it was proven that ORA has a remarkable reliability. The results obtained from the training set made it possible to detect the presence of transcripts with variable UTRs between *S. cerevisiae* strains. Finally, we propose a regulatory role for some non-coding transcripts conserved within the *Saccharomyces* genus and localized in the antisense strand to genes involved in meiosis and cell wall biosynthesis.

**Electronic supplementary material:**

The online version of this article (doi:10.1186/1471-2164-15-1045) contains supplementary material, which is available to authorized users.

## Background

Since the completion of its genome sequence in 1996 [1], almost all newly developed high throughput techniques have been applied to *Saccharomyces cerevisiae*.

Due to its high relevance as a model organism, differences in gene expression under different growth conditions in the same strain have been determined, while a smaller number of analyses have compared different yeast strains [2–5] or species [6, 7]. *S. cerevisiae* was also one of the first species in which transcriptome reconstruction using tiling arrays and RNA-seq was evaluated [8–10]. These studies allowed both a detailed determination of the transcriptome structure and the identification of entirely new classes of non-coding RNAs (ncRNAs) such as the Stable Untranslated Transcripts (SUT) [10, 11], whose function remains largely unknown. Although SUTs are pervasively transcribed, the majority of these ncRNAs have yet to be assigned a role, and their global functional significance remains controversial [12]. There are only a few exceptions of well-studied ncRNA such as *SRG1*, a ncRNA which is involved in the repression of *SER3* transcription [13, 14], and the ncRNA in antisense to the *IME4* gene that regulates entry into meiosis [15]. In contrast, in other yeast species such as *Schizosaccharomyces pombe,* a systematic program exists in which elevated antisense RNAs arising from both ncRNAs and overlapping convergent gene pairs determine a substantial reduction in protein levels throughout the genome [16]. These findings suggest that antisense transcripts can have a relevant role in fungal genomes.

Despite the high number of genomic studies, however, pivotal aspects of the transcriptome structure, such as its structural variability among different strains, have not been considered at all. The same is also true for the presence of non-coding transcripts in other species belonging to the *Saccharomyces* genus. The reason for this bias in literature studies is that the gene expression analysis performed on the same strain under different growth conditions is technically affordable, while the comparison of gene expression between different strains is more difficult. This is due to the lack of microarray platforms for many *S. cerevisiae* strains and *non-Saccharomyces* species. Arrays designed on a reference strain can actually be used for transcriptome analysis on other strains (or species), but this procedure can lead to biases in gene expression due to differences in the genomic sequences that can influence array hybridization.

Although new high-throughput sequencing techniques are a promising approach to absolve the need for “strain-” and “species-specific” microarrays, difficulties in transcriptome assembly and the lack of reference genomes, required for reads alignment, limits comparative studies on transcriptome structure. In principle, directional RNA-seq methods are more suitable than tiling arrays to deal with the complexity of the trascriptome, due to their single-nucleotide resolution, higher dynamic range and lower noise level [9, 17]. However, it has been demonstrated that in *S. cerevisiae*, tiling arrays are more precise for determining the transcriptome structure [10]. This is at least partly due to difficulties in managing the highly non-uniform coverage of the RNA-seq data that can produce gaps in the transcriptome assembly [18, 19]. Moreover, comparative studies on transcriptome structure have been so far neglected, perhaps due to an underestimation of the transcript variability between different yeast species and strains. However, recent studies have identified more than 13,000 RNA-binding proteins (RBPs) crosslinking sites in *S. cerevisiae*, of which a large number are localized in UTR regions. Since protein-RNA interactions are integral components of nearly every aspect of biology, a more detailed knowledge of the UTRs is needed. These results can help to better understand the role of this fundamental aspect of gene expression regulation and its variability in different *S. cerevisiae* strains and *Saccharomyces* species.

In recent years, two main categories of transcriptome reconstruction software tools have been developed: the reference-based software, that rely on reads previously aligned on a reference genome, and the “de-novo” assemblers that analyze RNA-seq reads without the need of a reference genome. Reference-based software include, for example, Cufflinks [20] and Scripture [21], while Trinity [22], Oases [23], TransAByss [24], Rnnotator [25] and Multiple-k [26] are de-novo assemblers.

Many of these software were developed to manage the transcriptome of higher eukaryotes, which are characterized by a large number of introns that determine extensive alternative splicing. Instead, with the exception of a software specifically developed for the identification of operons [27], transcriptome analysis of prokaryotes and lower eukaryotes rely only on programs developed for complex transcriptomes. These software cause an excessive fragmentation in transcriptome reconstruction which complicates further analysis. In contrast to higher eukaryotes, prokaryotes and some lower eukaryotes like *S. cerevisiae* are characterized by a low number of introns; a distinct characteristic that would require the use of specifically developed software. The presence of polycistronic transcripts must also be considered to obtain a reliable transcriptome reconstruction.

In order to fill this gap, a software named “ORA” (Overlapped Reads Assembler) was developed in this work. Furthermore, this software can be used for transcriptome analysis in prokaryotes because it can also deal with polycistronic transcripts. This work also highlights some of the main problems of the next-generation sequencing methods that made the reconstruction of the transcripts complex. Moreover, some computational solutions to improve software reliability were identified and integrated.

As a case study, the transcriptome of six yeast strains that have different origins was chosen. In this analysis, the laboratory strain S288c, a reference frequently used in gene expression studies, a commercial strain (EC1118) used in winemaking [28] and four vineyard strains (P283, R008, R103, P301) whose genomes have been recently sequenced [29] were considered. These strains were selected for their remarkable differences in fermentation that allowed their assignment into three classes of fermentation efficiency: “high” (EC1118, P283, P301), “intermediate” (R008) and “low” (P301, S288c). The availability of the sequences obtained by RNA-seq in these six strains presented an interesting opportunity to test our transcriptome assembly software. It was also possible to investigate some poorly studied characteristics, including transcriptome structure variability in different strains and the genomic distribution of ncRNAs with unknown functions such as the SUTs. These transcriptomes were analyzed in two different steps of the fermentation process: in mid-log phase and in early stationary phase. These two steps were named respectively “6 g/l” and “45 g/l” in relation to the amount of CO_2_ produced during alcoholic fermentation. Additionally, by comparing RNA molecules transcribed from orthologous genes, ORA allowed a global evaluation of UTR regions variability that can potentially influence the phenotype of the yeast strains. The widespread transcription of the ncRNAs localized in antisense to the protein-encoding genes was also confirmed in strains other than S288c and in three species of the *Saccharomyces sensu stricto* group (*Saccharomyces bayanus*, *Saccharomyces paradoxus* and *Saccharomyces mikatae*) that have accumulated approximately 5–20 million years of separate evolution [30]. This comprehensive overview of the transcriptome structure provided an estimation of the conservation level of these ncRNAs. Finally, our analysis allowed a functional role for a group of ncRNAs that are evolutionarily conserved among the *sensu stricto* group of yeasts to be proposed.

## Results and discussion

### The transcriptome assembly software

**Figure 1:**
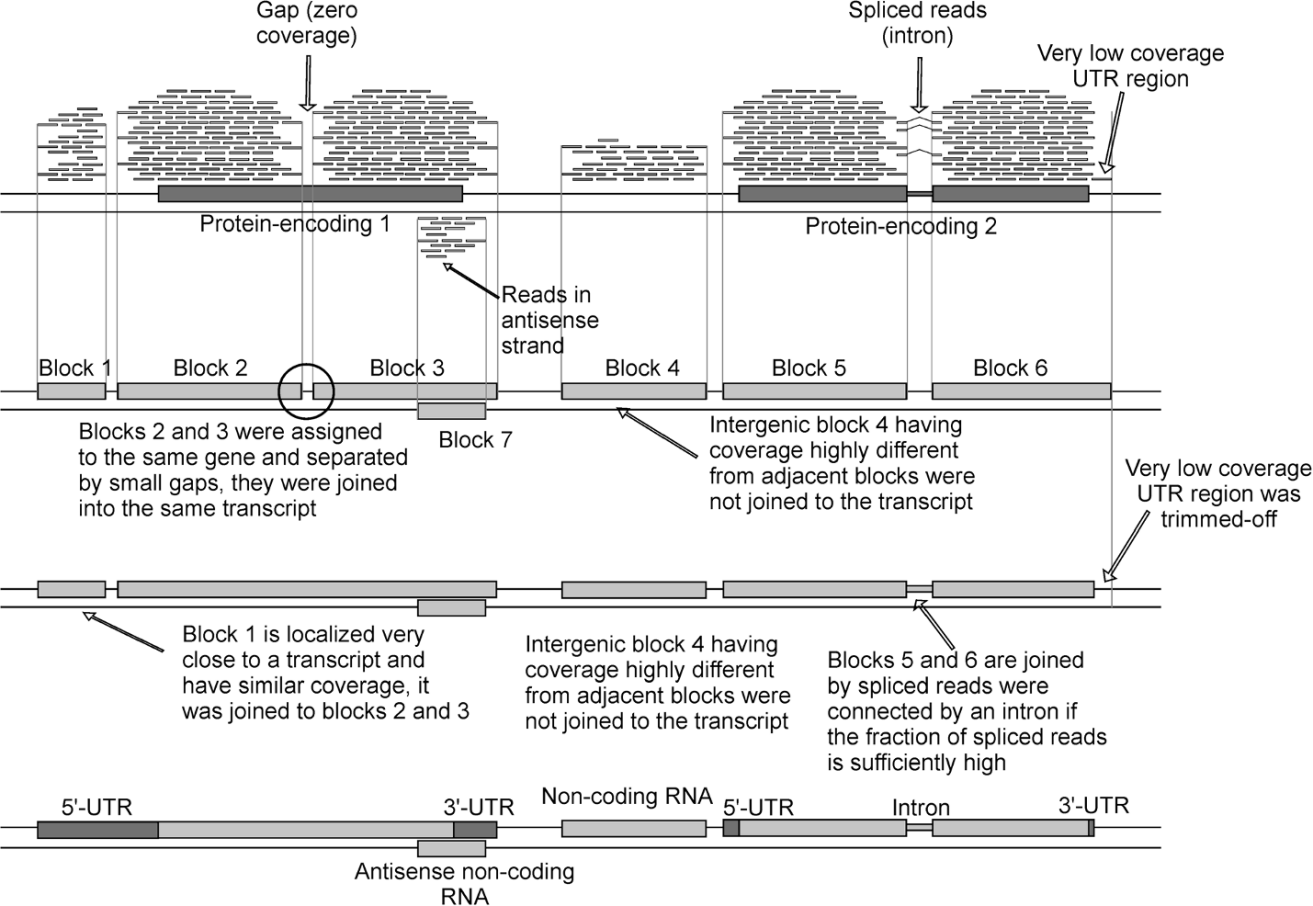
**Schematic representation of the transcriptome assembly process performed by ORA.** The circle indicates the gaps located between reference-based blocks.

ORA is a reference-based assembler taking RNA-seq reads aligned on a reference genome as input. The transcriptome assembly process is outlined in Figure 1 and described in detail in the Methods section. The main concept of the software is to join the overlapped reads aligned on the same strand to obtain “blocks” that, at best, encompass an entire transcript. Here, the most common occurrence is to obtain transcripts composed by some “blocks” separated by very short gaps where the coverage is equal to zero, usually due to biases in the sequencing process and/or introns. Since it is known that only 5% of *S. cerevisiae* genes have introns, in most cases these gaps are due to biases in the sequencing process [27]. These sequencing biases were also pointed out in previous studies and can also be evidenced in the coverage profile by the presence of peaks localized in the corresponding genomic position which are separated by low-coverage regions. This suggests that some genomic regions are more prone to sequencing than others. On the other hand, in a low number of cases, the internal gene regions with no coverage can be determined by the presence of introns. However, true introns are identified by a specific part of the software that focuses on “spliced” reads identification (Figure 1). At the end of the assembly procedure, the program provides the predicted transcripts, their coverage, the position on the genome, the gene(s) comprised in the transcribed region and the UTRs sizes.

Data reported in this paper refer to different species of the *Saccharomyces* genus, but ORA can be used also in prokaryotes and in lower eukaryotes with a small number of introns. As an example the transcriptome structure of *Naumovia castellii* [31] was analyzed and results are reported in Additional file 1: Table S1 and Additional file 2: Figure S2.

### Comparison of the transcriptome reconstruction obtained using different methods

**Figure 2:**
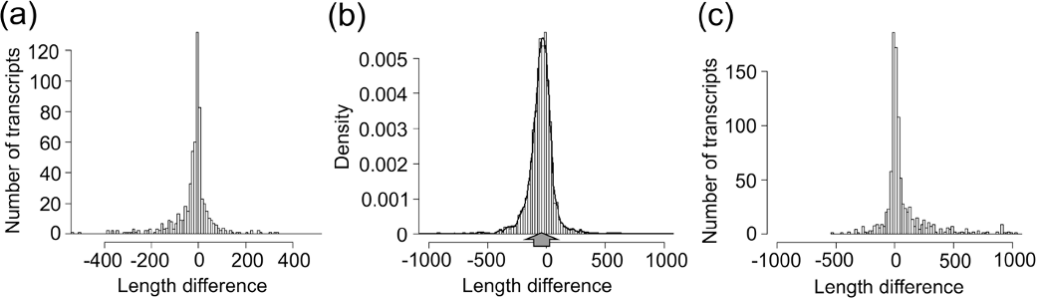
**Comparison between UTR sizes predicted using different methods. (a)** Comparison between the 5’-UTR size predicted by ORA and 5’-RACE in the S288c strain. Positive values indicate transcripts with larger 5’-UTR size in the prediction obtained with ORA. **(b)** Comparison between the 3’-UTR size obtained with ORA and the tiling arrays (S288c strain). Positive values indicate transcripts with larger 3’-UTRs in the prediction obtained using ORA. Note the slight underestimation of the 3’-UTR size obtained using ORA. **(c)** Histogram reporting the difference between the length of the 5’-UTR in S288c predicted by Cufflinks and by 5’-RACE. Positive values indicate a larger 5’-UTR determined by Cufflinks. Note the slight overestimation of the 5’-UTR size obtained using Cufflinks.

To determine the accuracy of ORA in the transcriptome assembly, we compared the results obtained with two previously published analyses (Table 1). The first [9] was performed using the 5’-RACE method that provides a reliable identification of the 5’-end of transcripts, while the second method [10] is based on tiling arrays. In the last procedure the transcripts identified were manually refined to improve the prediction. The results of these two transcriptome reconstructions have been previously compared [10] and it was demonstrated that 81% of the 5’-UTR predictions differed in less than 50 bases (Table 1). This threshold was used for comparing the results obtained in different experiments, which were considered reliable when differing in less than 50 bases. Comparison of data obtained with 5’-RACE and ORA revealed that 72% of the predictions (446 out of 615) for the 6 g/l experiment are reliable (Figure 2a). This percentage rose to 74% (307 out of 415) when considering only the transcripts with a coverage higher than 20. Similar values were obtained for the experiment performed at 45 g/l (Table 1).

**Table 1:** **Comparison among different methods used for transcriptome reconstruction**

		ORA (SOLiD)	Cufflinks (SOLiD)	Tiling arrays	5’-RACE	Illumina	
**5’-UTRs**							
**ORA (SOLiD)**	**45 g/l ^**	**-**	nd*	(1753/2473) 71% *	(446/615) 72% *	(1712/2298) 75% *	6 g/l*
**Cufflinks (SOLiD)**		nd ^	**-**	(1836/2903) 63% *	(586/885) 66% *	(1750/2656) 66% *	
**Tiling arrays**		(860/1336) 64% ^	(1882/3149) 60% ^	**-**	(1039/1281) 81% *	(3092/4180) 74% *	
**5’-RACE**		(488/721) 68% ^	(572/974) 59% ^	(1039/1281) 81% ^	**-**	(786/1009) 78% *	
**Illumina**		(1784/2739) 65% ^	(1599/2906) 55% ^	(3092/4180) 74% ^	(786/1009) 78% ^	**-**	
**3’-UTRs**							
**ORA (SOLiD)**	**45 g/l ^**	**-**	nd*	(1125/2473) 45% *	nd *	(1348/2527) 53% *	6 g/l*
**Cufflinks (SOLiD)**		nd ^	**-**	(1293/2903) 45% *	nd *	(1356/2923) 46% *	
**Tiling arrays**		(643/1336) 48% ^	(1491/3149) 47% ^	**-**	nd *	(2774/4551) 61% *	
**5’-RACE**		nd ^	nd ^	nd ^	**-**	nd *	
**Illumina**		(1609/3040) 53% ^	(1621/3214) 50% ^	(2774/4551) 61% ^	nd ^	**-**	

Percentages of 5’-UTRs and 3’-UTRs regions determined using different methods and software and having length differences of < = 50 bases. For each comparison, the number of UTR regions with a length difference of < = 50 bases and the total number of UTRs identified with both methods are shown in parenthesis. “SOLiD ORA” refers to the transcripts determined from our experiment using ORA, “Tiling arrays” refers to the data reported by Xu and colleagues [10], “5’-RACE” and “Illumina sequencing” refers to data reported by Nagalakshmi and colleagues [9] and “SOLiD Cufflinks” refers to the transcripts reported in our experiments and analyzed using Cufflinks [20]. In the top-right half of both matrices are reported 6 g/l results (marked using * symbol), in the bottom-left half of both matrices are reported 45 g/l results (marked using ^ symbol).

It is noteworthy that the previous data were obtained from very similar growth conditions and were manually refined to improve the prediction. On the contrary, ORA transcripts were determined in different growth conditions and were not manually refined. For this reason, the reliability of ORA predictions in this case is underestimated and it could be expected that in the same growth condition the results would be much more reliable.

The comparison of data obtained with ORA and those determined with tiling arrays revealed that 71% of the predictions (1753/2473) have a difference equal to or lower than 50 bases in the experiment performed at 6 g/l, a value that rises to 73% (1102/1501) when considering only the transcripts with coverage higher than 20. Similar values were obtained in the experiment performed at 45 g/l. Lower percentages (~48%) were obtained when considering the 3’-end of transcripts. The underestimation of the transcripts sizes is due to the loss of the reads overlapped to the polyadenylation site that were removed by the analysis software of the sequencer (see Methods section) (Figure 2b).

**Figure 3:**
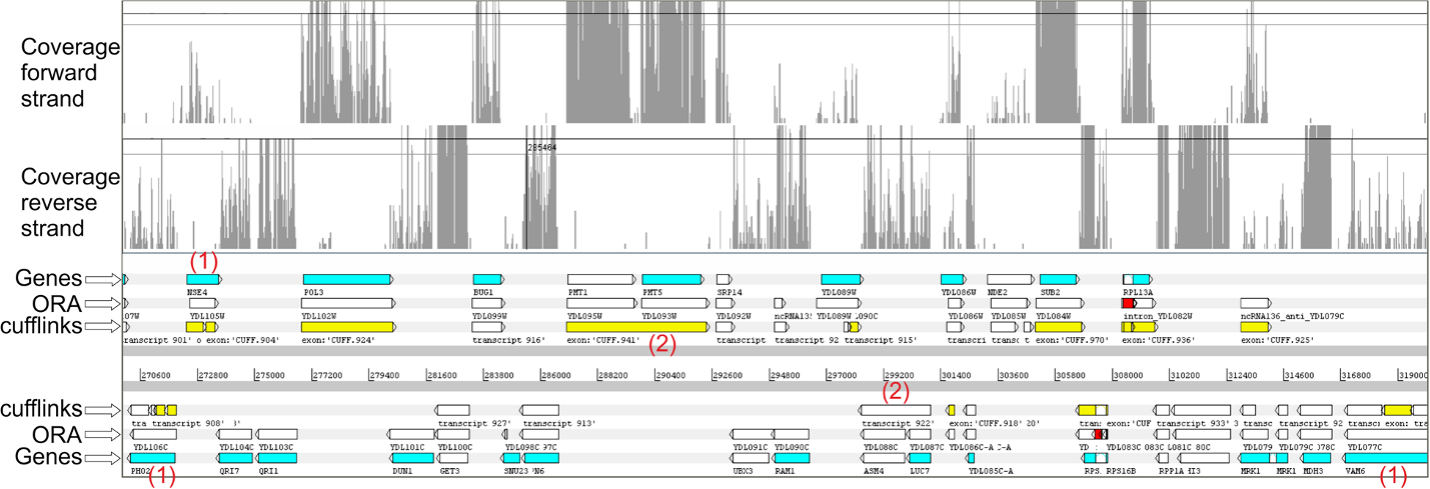
**Transcripts predicted in a region of** ***S. cerevisiae*** **chr IV (strain S288c) comprised between ~270.600 bp and ~319,000 bp.** From top to bottom: coverage on the forward strand, coverage on the reverse strand, genes (protein-encoding regions) (Genes), reconstruction of the transcripts obtained with ORA (ORA) and with Cufflinks (Cufflinks). In the row reporting the predictions of ORA, the introns are colored in red. Red numbers indicate key differences in transcript reconstruction between the two software: (1) transcripts formed by multiple “blocks” in the reconstruction with Cufflinks which are determined by the presence of gaps with no coverage in the coding region, (2) adjacent genes joined in polycistronic transcripts by Cufflinks despite large coverage differences.

Another comparison was performed between ORA and Cufflinks [20] on the same dataset (Figure 3). The predictions obtained for S288c were compared and the two software assembled a similar number of transcripts: 3254 from ORA and 3557 from Cufflinks. Using again the results obtained with tiling arrays as a reference [10] and 50 bp difference as a threshold it was found that ORA predicted 13% more reliable transcripts than Cufflinks (72% vs. 59%, Table 1). Using the results obtained with 5’-RACE experiment [9] as a reference, ORA predicted about 11% more reliable transcripts than Cufflinks (71% vs. 60%, Table 1).

The main discrepancy with RACE is due to an overestimation of the 5’-UTR size obtained using Cufflinks (Figure 2c), probably caused by the very low coverage regions localized at the ends of the transcripts. The same underestimation at the 3’-end seen with ORA was highlighted by Cufflink. In fact the low percentage of reliable transcripts (47%) is again due to the “loss of sequences” at the 3’-end in the SOLiD sequencing. This result confirms the sequencing method as the principal cause.

**Figure 4:**
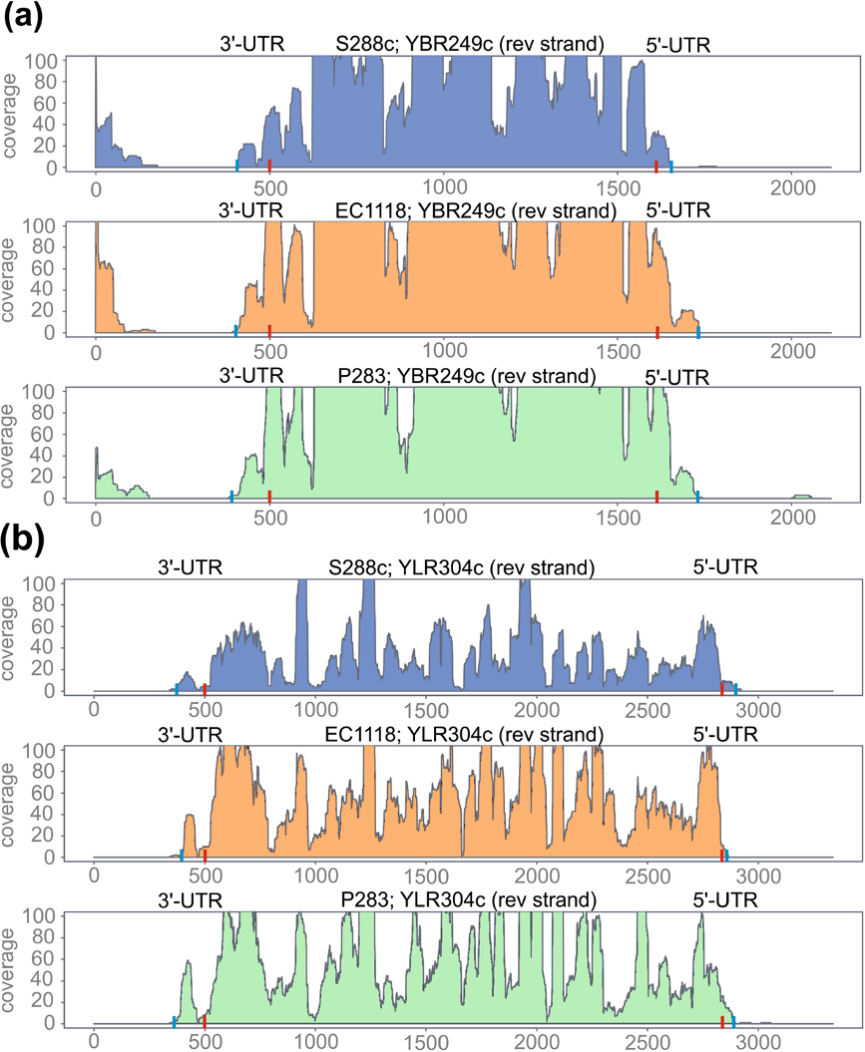
**Two examples of the transcript structure obtained in the reference strain S288c and vineyard strains EC1118 and P283. (a)** Transcript reconstruction of the gene YBR249C (*ARO4*, 3-deoxy-D-arabino-heptulosonate-7-phosphate (DAHP) synthase) at 6 g/l. **(b)** The transcript of the gene YLR304C (*ACO1*; aconitase, required for the TCA cycle) at 45 g/l. The red and blue rods indicate the end of the UTR region and transcript, respectively. The y axis reports the coverage, while the x axis shows the relative position. In both examples, the genes are encoded in the reverse strand, and consequently the 5’-UTR is on the right part of the graph.

Finally, a manual check of specific transcripts was performed to ensure maximum accuracy of the results, as small inconsistencies were observed in the reconstruction process. To simplify this process, a custom perl script was developed to visualize the coverage of the six strains analyzed in a graph (Figure 4, Additional file 3: Figure S2).

### Analysis of the UTR regions in six *S. cerevisiae*strains

Based on the reliable ORA transcriptome structure prediction, the structure of the 5’-UTRs of six *S. cerevisiae* strains (S288c, EC1118, P283, R008, R103, P301) was investigated (Additional file 4: Table S2). Strong phenotypic differences between these strains were found in a previous study, with the most relevant being those involved in determining the alcoholic fermentation performance [29]. Moreover, the phenotypic characters of these strains have been correlated to their gene expression profiles [32].

In order to maximize the reliability of the prediction, this analysis focused on the transcripts with a coverage higher than 20 (in all strains) (Additional file 5: Table S3), which amounted to 1124 in the experiment at 6 g/l and 768 at 45 g/l. ORA identified between 13% to 20% of the transcripts with variable UTRs at 6 g/l (depending on the strains pair considered), and between 17% to 28% at 45 g/l. Transcripts with variable UTRs were considered those that differed by 50 bases or more. The highest number of variable transcripts was found when comparing S288c with the other strains at 45 g/l. This is due to the greater diversity of this laboratory strain compared to the others that are much more similar at a genomic level [29]. Considering the six strains and the two growth conditions in this work, 30 pairwise comparisons were analyzed. In order to verify if specific functional classes of genes tend to have differences in the transcript structure between strains we performed a Gene Ontology (GO) analysis. We considered three group of genes: those with a 5’-UTR highly conserved between different strains (conserved genes), genes with variable 5’-UTRs (more than 50 bases) in at least one comparison between strains (variable genes) and genes with their 5’-UTR varying (more than 50 bases) in 6–14 comparisons (highly variable genes). No genes varied in more than 14 comparisons in a specific growth condition.

The results highlighted some GO functional classes significantly enriched in genes with highly conserved 5’-UTRs and others enriched in genes with variable 5’-UTRs. Three GO categories are noteworthy for their possible impact on the metabolic processes related to alcoholic fermentation (Table 2). The most relevant results are the presence of 22 genes with variable 5’-UTRs found at 6 g/l classified in the “sulfur compound metabolic process” category, 5 genes found in the “amino acid catabolic process to alcohol via Ehrlich pathway” category, and 13 genes involved in sterol metabolic process (Table 2). The first finding is of particular interest because genes involved in the “sulfur compound metabolic process” are important for the oxidative stress response and resistance to sulfur dioxide in yeast. In fact, SO_2_ is a preservative compound widely used in foods and beverages and is toxic at high concentrations [33]. Genes involved in SO_2_ detoxification (i.e. *MET1*, *MET5*, *MET8* and *MET10*) were previously shown to be induced after exposure of yeast cells to this compound [34]. The Ehrlich pathway instead is involved in the production of long chain and complex alcohols, representing one of the main classes of fermentation flavors in wine [35, 36]. The third very important class of genes interestingly includes both genes with highly conserved 5’-UTRs and also with variable 5’-UTRs (Table 2). Concordantly, a high variability in the expression level of genes involved in the biosynthesis of sterols in different yeast strains has been previously reported [32]. It is known that incorporation of sterols into membranes counteracts the stress induced by the high ethanol concentrations that accumulate during alcoholic fermentation [37].

**Table 2:** **Selected results obtained from GO analysis**

GO category	Characteristics of the category	Genes	***p-***value
GO:0006790 - Sulfur compound metabolic process	Variable 5’-UTR in *S. cerevisiae* strains	*CYS3, MIS1, PHO3, THI2, MET6, YHR112C, BAT1, MET1, IRC7, GLO2, THI4, TRX2, YLL058W, GTT2, SAM1, GSH2, THI20, THI80, GLR1, THI6, MRI1, THI22*	1.5*10^−5^ (6 g/l)
GO:0000947 - Amino acid catabolic process to alcohol via Ehrlich pathway	Variable 5’-UTR in *S. cerevisiae* strains	*SFA1, ADH4, PDC5, ADH3, ADH1*	6.4*10^−4^ (6 g/l)
GO:0016125 - Sterol metabolic process	Highly conserved 5’-UTR in *S. cerevisiae* strains	*ERG28, NCP1, NSG1, UPC2, ERG26, ERG4, ERG20, HMG2, ERG5, ERG12, ERG8, MVD1, HES1, ERG10, DAP1*	6.1*10^−6^ (6 g/l)
GO:0016125 - Sterol metabolic process	Variable 5’-UTR in *S. cerevisiae* strains	*RSP5, ERG11, ERG7, ERG9, ERG25, ERG3, ERG27, ERG6, ERG2, CYB5, ERG24, IDI1, KES1*	1.1*10^−3^ (6 g/l)
GO:0055085 - Transmembrane transport	Conserved SAUT among strains at 6 g/l	*SUL1, YHK8, PDR11, VCX1, AZR1, PEX21, YMR279C, YNL095C, FRE7, CTR1*	0.0052 (6 g/l)
GO:0031505 - Fungal-type cell wall organization	Conserved SAUT among *S. cerevisiae* strains at 45 g/l	*TIP1, UTR2, SIM1, TAX4, SPO75, YLR194C, WSC2, HPF1*	0.0059 (45 g/l)
GO:0006820 - Anion transport	Conserved SAUT among *S. cerevisiae* strains at 45 g/l	*BAP2, SUL1, FAT3, FET3, YMR279C, ATO2, FAA1*	0.019 (45 g/l)
GO:0022413 - Reproductive process in single cell organisms	Conserved SAUTs among *sensu stricto* yeast species	*RRT12, SPO77, SMA2, PRM1*	1.66*10^−3^

Relevant results obtained from analysis of the enrichment of genes involved in selected GO processes. Enrichment was calculated with respect to the entire set of *S. cerevisiae* genes using YeastMine and the *p-*value is reported on the rightmost column (http://yeastmine.yeastgenome.org/yeastmine/begin.do).

It is still difficult to identify a causal relationship between variations in the transcript structure, its expression and the final phenotypic effect. However, the variability of the transcript structure of genes belonging to specific functional classes suggests that this relationship may actually exist.

A manual inspection was carried out on the most interesting genes (Figure 4, Additional file 3: Figure S2).

One of these is *ARO4*, a gene involved in the first step of aromatic amino acid biosynthesis [38]. The 5’-UTR region is longer in the oenological strains with a size between 109 and 123 bases, compared to S288c where it is only 43 bases long. In the terminal part of this transcript at 56–70 bases from the 5’-end a binding site for a RBP was identified by gPAR-CLIP [39]. From ORA’s prediction it could be concluded that this binding site is absent in S288c since this strain lacks the terminal part of the transcript. Despite in enological strains the 5’-UTR region of *ARO4* is longer, it does not overlap the predicted TATA box (Additional file 6: Figure S3).

Finally, analysis of the RBPs was extended to all the binding sites previously identified [39] (Additional file 7: Table S4) and *ARO4* resulted not to be an exception. In fact, among the 4576 biding sites identified in the 5’-UTR region of 2550 genes, 546 are absent in one or more strains at 6 g/l, while 531 are absent in transcripts identified at 45 g/l. Accordingly, it is possible that differences in the 5’-UTR region of the transcripts could represent a major source of variability among *S. cerevisiae* strains. More generally, this feature, hitherto neglected, could play a relevant role also among strains of other species, with an impact on the stability of the transcripts and on the expression level.

### Non-coding transcripts (SUT-SAUT) in *S. cerevisiae*and in other yeast species

**Figure 5:**
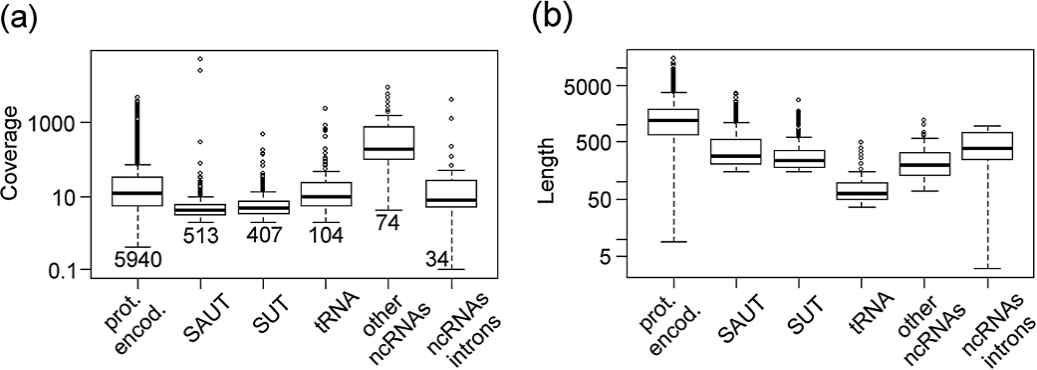
**Coverage (a) and length (b) of six classes of transcripts identified by ORA in the S288c strain at 6 g/l.** From left to right are reported: transcripts encoding proteins (prot. encod.), non-coding transcripts localized in antisense to other genes (mainly protein-encoding) (SAUT), non-coding transcripts localized in intergenic regions (SUT), tRNAs, other non-coding RNAs (mainly small nuclear RNAs) and ncRNAs localized in intronic regions. The number of transcripts identified for each class in the S288c strain at 6 g/l is shown in **(a)**.

As shown in previous studies [10, 11], the yeast transcriptome includes numerous ncRNAs localized in intergenic regions or in antisense to protein-encoding genes [10]. A high strand-specificity of the reads obtained from sequencing is important for the identification of SAUTs. This specificity was evaluated by determining the frequency of the SAUT identified in antisense to genes with different coverage values (Additional file 8: Figure S4). From the results obtained, the number of SAUTs was higher in genes with low expression suggesting that they are not influenced by reads lacking strand-specificity. Their expression level determined by ORA is on average lower than expression of protein-encoding genes (Figure 5) and of structural RNAs of known function (i.e. snoRNAs). The low expression level (Figure 5) and lack of a functional role for most SUTs have cast doubt upon their global functional significance [12] and suggest that some SUTs could be the result of a “transcriptional background”. In order to clarify their putative role and their effect on transcription, we classified SUTs by considering their localization with respect to other genes. Those found in intergenic regions were named as SUT (Stable Untranslated Transcripts) and those located in antisense to protein-encoding genes as SAUT (Stable Antisense Untranslated Transcripts). SAUTs are the more interesting for their putative effect on the transcription and translation of the gene located on the antisense strand [15, 16]. An examination of the putative effect of SAUTs on gene expression in the opposite strand (data not shown) did not reveal a common mechanism. In fact, depending on the gene, increased expression of a SAUT corresponded to either decreased or increased expression of the antisense gene, and sometimes had no effect at all. This finding suggests that only a fraction of the SAUTs identified have a functional role and they do not have a common mechanism of action.

To have a better understanding of the possible role of these transcripts and their conservation level in different strains, we verified the presence of SAUTs on the antisense strand of the orthologous genes of the six yeast strains analyzed. If an orthologous gene had a SAUT in the antisense region in all the six strains tested, the SAUT was considered to be conserved. Despite the number of SAUTs in the genomes being quite high (between 401 to 1209 depending on the strain), only 66 of them are conserved in all of the strains at 6 g/l and 117 at 45 g/l. Considering only the conserved SAUTs, we focused our attention on those with a potential role in the cell, while omitting “strain specific” ones and those representing a sort of “transcriptional background”.

GO analysis of the protein-encoding genes present in the antisense strand of conserved SAUTs was performed. Only a small number of biological processes were identified and two of them seem to be particularly affected by the presence of conserved SAUTs: transmembrane transport and fungal-type cell wall organization (Table 2).

**Figure 6:**
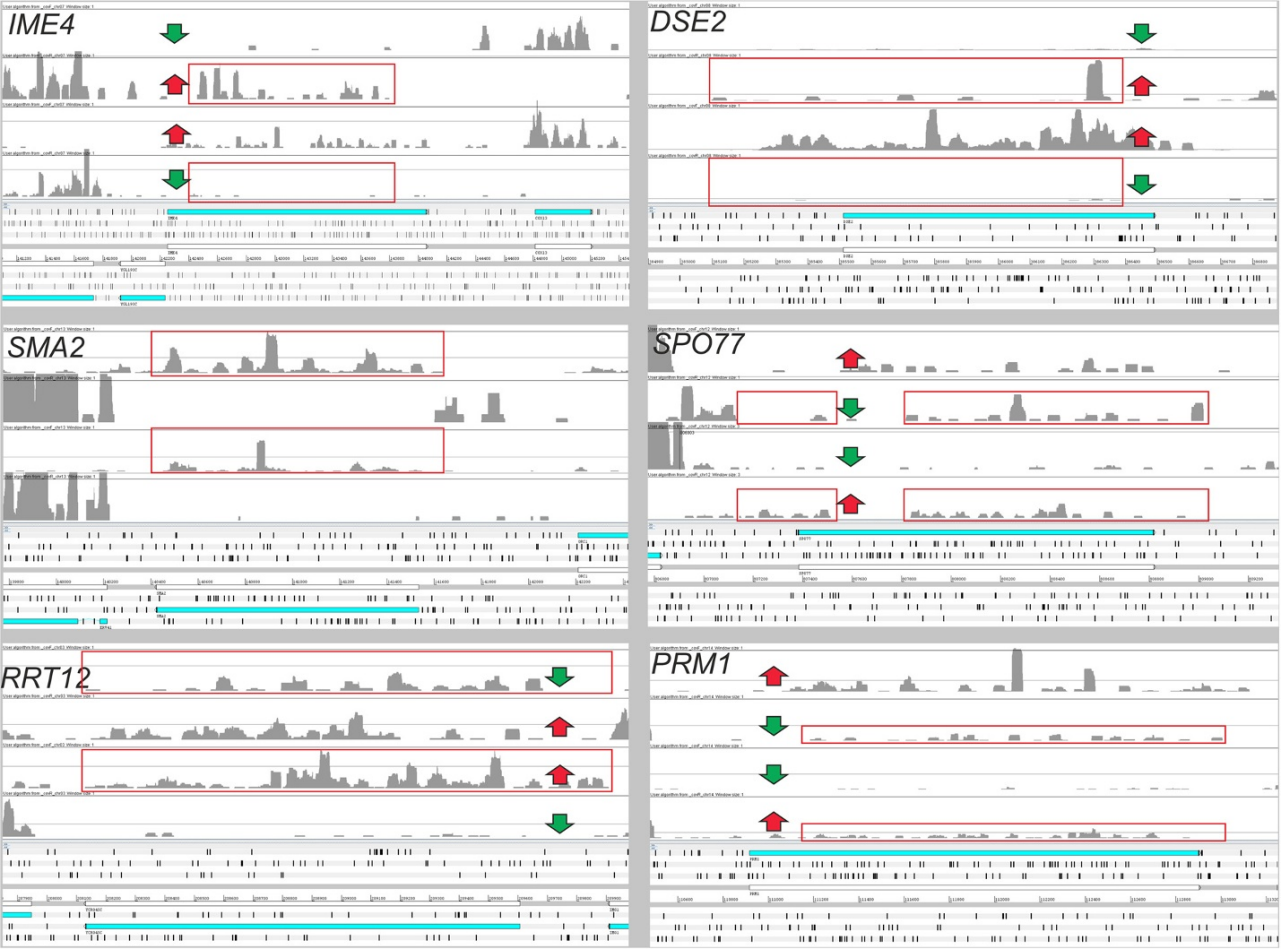
**Coverage profiles on forward and reverse strands for six selected genes.** Genes reported in figure belong to the GO categories “reproductive process in single-celled organism” (*RRT12*, *SMA2*, *SPO77*), “sporulation resulting in formation of a cellular spore” (*IME4*) and “fungal-type cell wall” (*DSE2*). SAUTs conserved in all the *Saccharomyces* species analyzed (indicated by red boxes) were found in all the genes except *IME4* and *DSE2*. An inverse correlation in gene expression between the protein-encoding transcript and the SAUT is highlighted by red/green arrows and was previously demonstrated for *IME4*[15].

In order to widen the analysis at a higher taxonomic level, the existence of conserved SAUTs was evaluated in three species of the *Saccharomyces sensu-stricto* group recently sequenced: *Saccharomyces paradoxus*, *Saccharomyces bayanus* and *Saccharomyces mikatae* [40]. High throughput sequencing data obtained for these three species and also for a diploid *S. cerevisiae* strain were used [6]. Their transcriptome structure was predicted with ORA and SAUTs were identified. Like for the previous analysis performed on the six *S. cerevisiae* strains, only genes with a SAUT in the antisense strand in all the four species were considered. Only two genes (*OPT2* and YDL129W) had SAUTs in all the four species. However, this number rises to 25 (Table 3) when considering the results previously obtained for S288c in a gene expression study [36]. This discrepancy can be explained by the fact that the S288c strain analyzed by Busby and colleagues [6] is diploid, while the strain considered by Treu and colleagues [36] is haploid. It is known that the expression of the antisense transcripts (SAUTs) of some *S. cerevisiae* genes (i.e. *IME4*; YGL192W) decreases in the diploid strain to allow the expression of the protein-encoding gene on the opposite strand [15]. *IME4* encodes a N6-adenosine methyltransferase required for entry into meiosis that has high expression levels in diploid cells (Figure 6). In haploids, antisense transcription prevents sense transcription of the *IME4* gene by means of transcription interference. In this process, a strong constitutive transcription of the *IME4* antisense interferes with transcription of the sense RNA. Other genes with a lower expression in the haploid strain were previously identified [41] and some of these are involved in the biosynthesis of the cell wall like *DSE2* (YHR143W), a daughter cell-specific secreted protein with similarity to glucanases. Considering normalized values obtained for the S288c haploid and diploid strains, it was found that SAUTs in antisense to the genes *DSE2* and *IME4* are more highly expressed in the haploid strain. They are also inversely related to the expression level of the protein encoding transcript (Figure 6). This finding suggests, therefore, that not only *IME4* but also *DSE2* is regulated by an antisense transcript.

**Table 3:** **Protein-encoding genes with a SAUT in the antisense strand**

Gene systematic name	Gene standard name	Gene name
YPR194C	*OPT2*	Oligopeptide Transporter
YDL129W	*-*	-
YOR042W	*CUE5*	Coupling of Ubiquitin conjugation to ER degradation
YOR040W	*GLO4*	Glyoxalase
YNR002C	*ATO2*	Ammonia (Ammonium) Transport Outward
YNL279W	*PRM1*	Pheromone-Regulated Membrane protein
YKL151C	*-*	-
YJR129C	*-*	-
YDR242W	*AMD2*	Amidase
YDR124W	*-*	-
YBR033W	*EDS1*	Expression Dependent on Slt2
YML066C	*SMA2*	Spore Membrane Assembly
YKL187C	*FAT3*	Fatty acid transporter 3
YHR177W	*GON3*	Regulator Of Fluffy
YGL224C	*SDT1*	Suppressor of Disruption of TFIIS
YDR222W	*-*	-
YCR045C	*RRT12*	Regulator of rDNA Transcription
YPL021W	*ECM23*	ExtraCellular Mutant
YMR182C	*RGM1*	-
YML118W	*NGL3*	-
YLR341W	*SPO77*	Sporulation
YKR102W	*FLO10*	Flocculation
YGL251C	*HFM1*	Helicase Family Member
YGL059W	*PKP2*	Protein Kinase of PDH
YER176W	*ECM32*	ExtraCellular Mutant

Protein-encoding genes with a SAUT in the antisense strand in all the species of the *Saccharomyces* genus analyzed (*S. cerevisiae*, *S. bayanus*, *S. paradoxus*, *S. mikatae*).

Of the 25 genes identified by the comparison performed among the *sensu-stricto* yeast species, it was found that *SMA2* (YML066C) and *SPO77* (YLR341W) encode meiosis-specific proteins (Figure 6). The protein encoded by *SMA2* is required to produce the bending force necessary for the proper assembly of the prospore membrane during sporulation [42, 43], while *SPO77* is required for spore wall formation during sporulation [42, 44, 45]. *PRM1* gene is a pheromone-regulated multispanning membrane protein involved in membrane fusion during mating [46]. Among the 25 genes there is also *RRT12,* a subtilisin-family protease involved in spore wall formation [47], with a role in dityrosine layer formation. Other previous experiments confirmed that the expression of some proteins involved in biosynthesis of the spore wall is lower in diploids than in haploid cells [48]. The regulation of spore wall biosynthesis by the presence of SAUTs is a major finding and underlines the importance of coordinating the dynamic remodeling of the yeast wall with cell morphogenetic events.

These 4 genes (*PRM1*, *SPO77*, *SMA2*, *RRT12*) belong to the GO class “reproductive process in single-celled organism” that is enriched in genes with an antisense transcript. It is clear that not all of these genes have a common mechanism for regulating the expression level of the protein-encoding gene (as in the case of *IME4* and *DSE2*) (Figure 6). Nevertheless, the high conservation level of SAUTs in different yeast species suggests a functional role for these transcripts in the control of protein expression. In particular, entry into meiosis is a key developmental decision and antisense ncRNAs seem to play a relevant role in various steps of this process. These data revealed here for the first time a general role for a group of SAUTs both in *S. cerevisiae* and probably in other species belonging to the sensu-stricto group of yeasts.

## Conclusions

In this work, a software for the analysis of transcriptional landscapes of lower eukaryotes and prokaryotes was specifically developed. This software, named ORA, proved to perform very well when compared with other software and produced results comparable to those obtained *via* reliable methods such as 5’-RACE. ORA proved to be a very useful tool for the identification of 5’- and 3’-UTRs starting from RNA-seq reads. In this study the variability of the transcriptome structure of different *S. cerevisiae* strains was highlighted. Furthermore, it was discovered that this variability can influence the binding sites of the RBPs in regions localized at the 5’-UTR. This finding indicates that differences in UTRs can change the stability and the translation efficiency of the transcripts in different strains. Finally, we have demonstrated that ORA allows the identification of “criptic” transcripts such as SAUTs. In conclusion, this new software is able to simplify comparative analyses between strains and species and, in some cases, the results can be used to suggest a functional role for unknown transcripts.

## Methods

### Yeast strains

Vineyard strains P283, P301, R008, R103 were obtained from previous yeast selection program [29]. The laboratory strain S288c was purchased from CBS collection (CBS8803). The industrial wine strain Lalvin EC1118 (EC1118), also known as “Prise de mousse”, is a *S. cerevisiae* wine strain isolated in Champagne (France) and manufactured by Lallemand Inc. (Montreal, CA). EC1118 has been deposited in the Collection Nationale de Cultures de Microorganismes (Institut Pasteur, Paris, France) as strain I-4215.

### RNA-seq analysis performed using the SOLiD 3 sequencing instrument

Cells were collected during the fermentation process performed in monitored bioreactors. Three biological replicates of the fermentations were analyzed for each strain. Samples for RNA-seq were collected at the beginning of the process, when the CO_2_ produced by the cells was 6 g/l (mid log phase), and in the middle of fermentation when it was 45 g/l (early stationary phase). The total RNA was extracted from each sample using the RiboPure™-Yeast kit (Ambion, Carlsbad, CA, USA). The quality and the quantity of the total RNAs were measured using Nanodrop (ThermoFisher Scientific, Waltham, MA, USA) and the Agilent 2100 bioanalyzer (Agilent, Santa Clara, CA, USA). Four μg of each biological replicate were pooled together and freeze-dried. The RiboMinus™ Transcriptome Isolation Kit (Life Technologies, Carlsbad, CA, USA) was used to selectively remove rRNAs from total RNA. Libraries were prepared using the “SOLiD Whole Transcriptome Analysis Kit protocol” (Life Technologies) and generated approximately 30–50 Gbp reads 50 bases long. PASS software [49] was used to univocally align 9–25 million reads for each sample to the reference genome. Reads mapped on the repeated regions were discarded. RNA-seq data comply the standards proposed by the Microarray Gene Expression Data Society and were deposited in MIAME-compliant format to GEO database with accession numbers GSE44845, GSM1092504–GSM1092515. Data were also reported in previous papers [29, 32] where the influence of the promoter regions on gene expression has been investigated.

SOLiD reads obtained from *S. paradoxus* Y-17217, *S. mikatae* IFO1815, *S. bayanus* MCYC623 were downloaded from GEO database (accession number GSE32679).

Alignment results in SAM format were managed using SAMtools [50].

Coverage profiles and transcripts were visualized on the *Saccharomyces* genome using Artemis browser [51] or with a perl script embedding R commands that was used to plot the coverage.

Gene Ontology analysis was performed on the YeastMine database (http://yeastmine.yeastgenome.org/) considering the Biological Process ontology; GO category enrichment was calculated considering all the *S. cerevisiae* genes reported in the database.

### Analysis of the RBS in *ARO4*promoter

The number of binding sites in the *S. cerevisiae* genomes analyzed was determined using PATMAN software [52] and allowing from 0 to 3 variable positions in the predicted sequence. Results are reported in Additional file 6: Figure S3.

### Analysis of the *N. castellii*transcriptome

RNA-seq reads were downloaded from GEO database (GSE58884) and aligned on the reference genome using PASS software v1.6 [49]. The maximum distance allowed for “spliced reads” was 1 kb. Transcriptome reconstruction was performed using ORA with default parameters except the ratio between spliced and unspliced reads in intron identification that was increased to 0.65 to reduce false positives. Selected regions of the genome were visualized using Artemis [51] and are reported in Additional file 2: Figure S1.

### The transcriptome assembly software ORA

ORA takes as input reads aligned on a reference genome using software like PASS [49], or TopHat [53] which is based on Bowtie for reads alignment [54]. Only shotgun reads information is considered by ORA (and not paired-end reads) for two main reasons. The first reason is that generally in lower eukaryotes alternative splicing are absent or very rare and therefore the transcripts assembly process does not require the information of the paired-end reads. The second reason is that paired-end sequencing is not available for some of the sequencing methods recently introduced such as the proton and the ion-torrent [55], but these sequencers are becoming widespread for the low cost of both the instrument and the sequencing reagents.

A specific function developed in ORA uses the spliced reads information for introns identification.

As first step in the transcriptome reconstruction, the user is asked to select some of the parameters used for the transcriptome assembly, like the minimum size of gaps with no coverage (below which the software is forced to join two “blocks” together) and the minimum number of reads to be joined into a “block”. The last parameter is important to reduce the “background noise” in transcriptome reconstruction, determined by the presence of single or small groups of reads localized in intergenic regions or in genes having very low coverage. Default parameters suggested by ORA were optimized on species of the *Saccharomyces* genus with transcriptome sequenced using SOLiD or Illumina platforms [56]. Finally, in order to manual inspect the results obtained by the software, files containing the single-base coverage on each chromosome and/or scaffold of the genome are generated (Figures 3 and 4). These files can be visualized using Artemis software [51].

In the second step, the software classifies the “blocks” in two groups based on annotation. The first group includes “blocks” that are totally or partially overlapping to an annotated gene (named “reference-based blocks”). The second group includes the “blocks” that cannot be assigned to predicted genes. At each run, the software automatically calculates the average size of the gaps located between “reference-based blocks” matching the same gene (referred as GRBB) and their standard deviation (GRBB-SD) (Figure 1). The mean and the SD of the coverage ratio of these “blocks” are also calculated (referred as CRRBB and CRRBB-SD). These parameters are fundamental for the next step of the computation where ORA analyzes the “blocks” not assigned to any annotated gene (located in intergenic regions) to determine whether they can be joined with an adjacent “reference-based” block. The program joins two “blocks” when the distance between them and their coverage is similar (within a range of 2 SD in the distribution previously calculated, GRBB-SD and CRRBB-SD) (Figure 1). Otherwise, they are considered to be transcripts generated by genes not previously identified. At this step, as “blocks” with different coverage are merged, ORA re-calculates size and coverage of the new “reference-based blocks”. These steps are recursively repeated until no more “blocks” in intergenic regions can be further joined with pre-existing ones.

In the third and final step, the software generates the final transcriptome prediction. “Blocks” located within an annotated gene not separated by introns are merged reducing the transcript “fragmentation” (Figure 3). This is based on the assumption that the number of introns is extremely low and most of them can be detected through the spliced reads. To avoid the identification of false positive introns, the “spliced reads” coverage must be equal to or greater than 1/10 of the “unspliced reads” coverage in the intron-exon boundary. This ratio has been empirically determined considering the presence of “false positives spliced reads” randomly scattered on the genome and the presence of reads that are not correctly aligned on exons boundaries.

ORA has some modules designed to execute two additional analyses: one focused on polycistronic transcripts and another one on 5’ and 3’ ends. During the first analysis, ORA performs a further check to verify the reliability of polycistronic transcripts as their presence in *S. cerevisiae* was previously predicted [10]. The analysis is based on the method described by Campanaro and colleagues [57] and verifies whether two adjacent genes are part of a real polycistronic transcript or if the prediction is a false positive determined by the closeness of their transcripts on the genome (Figure 1). Reliability of this approach was previously determined [58] comparing results with a bioinformatics prediction method [59].

ORA identified a small number of bicistronic transcripts (19 in S288c), some of these YIL165C-YIL164C, YKL022C-YKL021C, YOR059C-YOR060C, YOR376W A-YOR377W, YFR033C-YFR034C, YDR481C-YDR482C were previously identified [8, 10]. Sixteen of these transcripts were identified only at 6 or 45 g/l and were not considered real fusion genes but distinct transcripts not separated by untranscribed intergenic regions. This result highlights the importance of comparing transcriptome reconstruction in different growth conditions. A validation of the remaining 3 bicistronic transcripts (YFR033C-YFR034C, YFL057C-YFL056C and YIL165C-YIL164C) was performed considering paired-end reads [56] and polyadenylation sites [60] (Additional file 9: Figure S5). Results suggest that only YIL165C-YIL164C is transcribed as a bicistronic transcript and this is expected because these two genes in closely related species and in some *S. cerevisiae* strain backgrounds likely constitute a single ORF encoding a nitrilase gene [61].

The second additional analysis is performed on transcripts with coverage higher than 20 to improve the prediction of their 5’ and 3’-ends (Figure 1). In a comparison between UTRs size determined by tiling arrays experiments and those determined by RNA-seq [10] it was seen that the latter method tends to overestimate the size of high coverage transcripts (Figure 2c). Due to its high sensitivity the RNA-seq detects very low abundance transcripts of unclear origin. Our results highlighted the presence of an “anomalous” elongation of the UTRs with “tails” having very low coverage. For this reason, in high coverage genes a better estimation of the transcript ends can be obtained introducing a trimming step. In this process the terminal parts of the transcript where the coverage become equal or lower than three were removed (Figures 2, 3 and 4). However, it remains to be clarified whether these 5’ and 3’-ends with very low coverage can have a functional value or if they are generated through anomalous transcriptional initiations.

One of the main problems in transcriptome reconstruction is the prediction of very low coverage transcripts, in particular those not assigned to a reference gene (i.e. *S. cerevisiae* SUTs). In specific cases, the transcript prediction tends to be splitted into separate portions and this led to an overestimation of the real number of ncRNAs. This issue is very hard to solve with currently available sequencing methods and for a reliable prediction very deep transcriptome sequencing is needed.

Another critical point was highlighted by our results from analysis of RNA-seq reads obtained using the SOLiD 3 platform: the SOLiD analysis software removes the reads partially or completely overlapped to the poly-A. This led to an underestimation of the 3’-UTR size (Figure 2). The lack of these reads was demonstrated through a specific check performed on the 3’-terminal sequences partially overlapped to the poly-A (data not shown). Due to the underestimation of the 3’-UTR size, in the Results and Discussion section we will focus only on the results obtained for the 5’-UTR regions.

ORA software was written in perl (5.17) and was tested in Linux (Ubuntu 12.04) operating system. The software can be downloaded from https://sourceforge.net/projects/transcriptomeassemblyora/files/ together with the manual and the test files. It requires approximately 5 Gb RAM to assemble 10–20 million sequences on a reference genome.

## Electronic supplementary material

Additional file 1: Table S1: Transcriptome reconstruction of *Naumovia castellii* performed using ORA software. The worksheet “UTRs” reports the length of the UTR regions in protein encoding genes. In column (a) is reported the gene name, in (b) the coverage, in (c) the 5’-UTR size [bp], in (d) the 3’-UTR size and in (e) the chromosome. The worksheet “Transcriptome” reported the output of ORA in “gff” format, in column (a) the chromosome, (d) transcript start, (e) transcript end, (g) strand and in column (i) are resumed the ID of the gene encoding the transcript (obtained from the gff annotation file), the length of the transcript and its coverage (reported as “note=”). In column (i) the transcripts expressed in antisense to other annotated genes are reported as “ncRNA_anti_gene ID in antisense strand”, while genes in intergenic regions are reported as “ncRNAs_ID of the closest annotated gene”. (XLSX 509 KB)

Additional file 2: Figure S1: The transcriptome reconstruction of *Naumovia castellii* obtained using ORA. Histograms reporting the size distribution of the 5’ and 3’-UTR size of *N. castellii* (a-b) and *Saccharomyces cerevisiae* S288c (at 6 g/l) (c-d). Notice the small 5’-UTR size of *N. castellii* (54 bp on average) in comparison to *S. cerevisiae* (81 bp on average). Analysis of the data allowed the identification of numerous introns in the 5’-end of the genes (frequently in the 5’-UTR) (red rectangles). Some of these introns were not previously predicted. (e) (h) Analysis of the 5’-UTR size revealed some genes having an incorrect prediction of the start codon (red arrows). In (e-h) the coverage in the forward and reverse strands determined using RNA-seq are indicated with “cov. for” and “cov. rev”, the transcriptome prediction obtained using ORA is indicated with “ORA” and the gene prediction obtained from ncbi database is indicates as “genes”. (PDF 756 KB)

Additional file 3: Figure S2: Selected results of the transcript structure obtained for the six strains under analysis. Genes reported belong to the GO categories “sulfur compound metabolic process” and “sterol metabolic process” that are described in the main text. Genes YDR213W and YGL012W have highly conserved 5’-UTRs and were reported in order to show that gene expression level has little influence on the transcript structure prediction. The 5’-end of the transcript is on the left for genes encoded on the forward strand and on the right for genes encoded on the reverse strand. 5’-UTR is indicated by a small arrow. (PDF 7 MB)

Additional file 4: Table S2: Transcriptome reconstruction performed using ORA software. Each worksheet reports the trascriptome structure of a *S. cerevisiae* strain or a *Saccharomyces sensu stricto* species. The worksheets “*S. cerevisiae*_S288c_haploid_6gl”, “*S. cerevisiae*_S288c_haploid_45gl”, “*S. cerevisiae*_EC1118_6gl”, “*S. cerevisiae*_EC1118_45gl”, “*S. cerevisiae*_P283_6gl”, “*S. cerevisiae*_P283_45gl”, “*S. cerevisiae*_R008_6gl”, “*S. cerevisiae*_R008_45gl”, “*S. cerevisiae*_R103_6gl”, “*S. cerevisiae*_R103_45gl”, “*S. cerevisiae*_P301_6gl”, “*S. cerevisiae*_P301_45gl” report the transcriptome structures determined for the *S. cerevisiae* strains S288c, EC1118, P283, R008, R103, P301 in two points of the fermentation curve (6 and 45 g/l). The worksheets “*S. cerevisiae*_S288c_diploid_1”, “*S. cerevisiae*_S288c_diploid_2”, “*S. bayanus*”, “*S. paradoxus*” and “*S. mikatae*” report the transcriptome structure determined for the *Saccharomyces sensu stricto* species whose transcriptomes were sequenced by Busby and coll. [6]. Each worksheet reports the output of ORA in “gff” format: in column (a) the chromosome, (d) transcript start, (e) transcript end, (g) strand and in column (i) are resumed the ID of the gene encoding the transcript (obtained from the gff annotation file), the length of the transcript and its coverage (reported as “note=”). In column (i) the transcripts expressed in antisense to other annotated genes are reported as “ncRNA_anti_gene ID in antisense strand”, while genes in intergenic regions are reported as “ncRNAs_ID of the closest annotated gene”. (XLSX 8 MB)

Additional file 5: Table S3: Comparison between 5’-UTR length in the six strains determined using ORA software. Results were obtained analyzing data obtained at 6 g/l (worksheet “6gl_5 UTR”) and at 45 g/l (worksheet “45gl_5 UTR”). On each worksheet are reported: (a) the primary SGD ID, (b) the gene name, (c) the gene description, (d-i) 5’-UTR length, (j-o) coverage, (p) the number of pairwise comparisons where the difference in 5’-UTR length is higher than 50 bases. (XLSX 432 KB)

Additional file 6: Figure S3: Analysis of the 5’-UTR region of *ARO4* gene. (a) To verify if the RBS in *ARO4* (YBR249c) promoter was differentially represented between oenological and laboratory strains, PATMAN software [52] (https://bioinf.eva.mpg.de/patman/patman-1.2.html) was used. In Freeberg and colleagues [39] the consensus sequences for RBSs were not provided, for this reason the number of RBS in the genomes was estimated allowing up to 3 mismatches in the sequence of the RBS obtained from S288c strain. Results indicate that the RBS is only slightly over-represented in S288c. Since the genome of the enological strains is not complete, results in the table were normalized considering the length of the genomes. (b) Visual representation of the *ARO4* promoter showing the predicted TATA box and the RBS. (PDF 249 KB)

Additional file 7: Table S4: Distance between the RNA binding sites (RBS) identified by Freeberg and colleagues [38] and the 5’-UTR of the transcripts. Negative values refer to the RBS localized upstream of the 5’-end of the transcript reconstructed by ORA; these RBS are not included in the transcript of that strain in that growth condition. Worksheet “6 gl” refers to the transcript structure determined using RNA-seq reads obtained from the first point of the fermentation curve (early stationary), while “45 gl” refers to the transcript structure determined in the second point of the fermentation curve (mid-log phase). (XLSX 95 KB)

Additional file 8: Figure S4: Directional reads validation. Frequency of antisense ncRNAs (named SAUTs in the paper) in highly expressed genes was determined and results are reported in (a). Frequency of genes having SAUT was calculated with respect to the coverage of the corresponding genes. Some selected images of highly expressed genes are reported (b-e) to show the high strand-specificity of the library, while two examples (f-g) are reported to show genes with high antisense transcription, SAUTs are highlighted by red boxes. (PDF 483 KB)

Additional file 9: Figure S5: Verification of bicistronic transcripts identified using ORA. Sixteen bicistronic transcripts were identified by ORA only in one of the two growth conditions (6 or 45 g/l) and were excluded from further investigation. This result highlights the importance of comparing transcriptome reconstruction in different growth conditions. The remaining three transcripts are reported in (a-c) and include three pairs of genes YFR033C-YFR034C, YFL057C-YFL056C and YIL165C-YIL164C. These transcripts were further verified considering two lines of evidence: the presence of polyadenylation sites previously determined (red boxes in the figure) [60] and the distribution of the paired-end reads obtained from a previous study (top of the figures) [56]. The first two bicistronic transcripts (a-b) are distinct transcripts not separated by untranscribed intergenic regions; this is evidenced by the high coverage differences, the paired-end distribution and/or the presence of polyadenylation sites. Only genes YIL165C-YIL164C seem to be transcribed in a real bicistronic transcript. Red arrows indicate putative transcription start sites. (PDF 369 KB)
