# Supplementary material for: Transcriptome structure variability in Saccharomyces cerevisiae strains determined with a newly developed assembly software
Source: BMC Genomics. 2014 Dec 1;15(1):1045. doi: 10.1186/1471-2164-15-1045 (PMC4302112; doi:10.1186/1471-2164-15-1045)
Supplement: Supplementary file 2 — Additional file 2: Figure S1: The transcriptome reconstruction of Naumovia castellii obtained using ORA. Histograms reporting the size distribution of the 5’ and 3’-UTR size of N. castellii (a-b) and Saccharomyces cerevisiae S288c (at 6 g/l) (c-d). Notice the small 5’-UTR size of N. castellii (54 bp on average) in comparison to S. cerevisiae (81 bp on average). Analysis of the data allowed the identification of numerous introns in the 5’-end of the genes (frequently in the 5’-UTR) (red rectangles). Some of these introns were not previously predicted. (e) (h) Analysis of the 5’-UTR size revealed some genes having an incorrect prediction of the start codon (red arrows). In (e-h) the coverage in the forward and reverse strands determined using RNA-seq are indicated with “cov. for” and “cov. rev”, the transcriptome prediction obtained using ORA is indicated with “ORA” and the gene prediction obtained from ncbi database is indicates as “genes”. (PDF 756 KB) [file 12864_2014_6763_MOESM2_ESM.pdf]

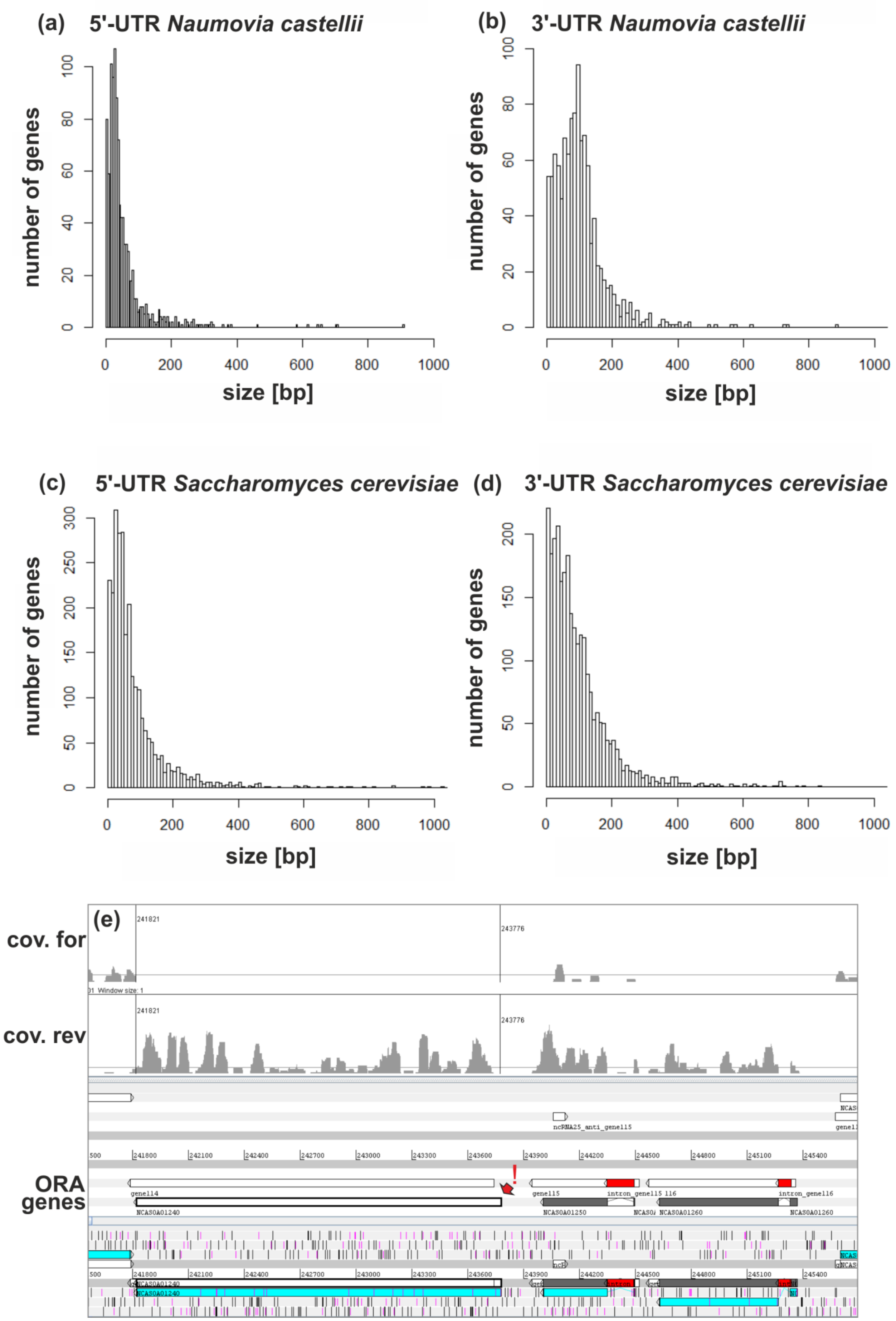

(a) 5'-UTR *Naumovia castellii*
number of genes
size [bp]
(b) 3'-UTR *Naumovia castellii*
number of genes
size [bp]
(c) 5'-UTR *Saccharomyces cerevisiae*
number of genes
size [bp]
(d) 3'-UTR *Saccharomyces cerevisiae*
number of genes
size [bp]
(e)
cov. for
cov. rev
ORA genes
241821
243776
gene114
NCASOA01240
gene115
NCASOA01250
NCASOA01260
intron_gene115
intron_gene116
ncRNA25_anti_gene115

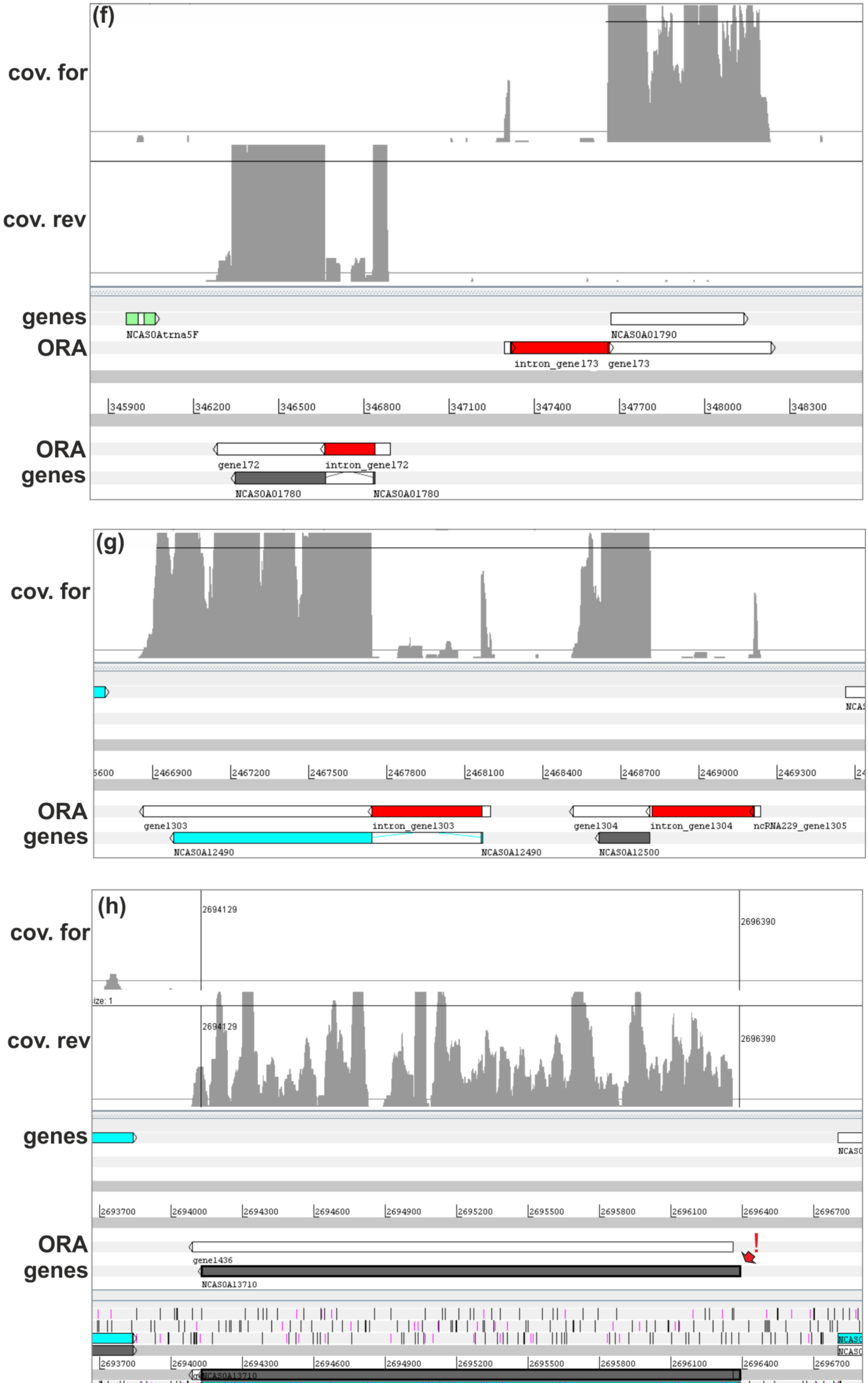
(f)
cov. for
cov. rev
genes
ORA
NCAS0Atrna5F
NCAS0A01790
intron_gene173
gene173
345900
346200
346500
346800
347100
347400
347700
348000
348300
ORA
genes
gene172
intron_gene172
NCAS0A01780
NCAS0A01780
(g)
cov. for
2466900
2467200
2467500
2467800
2468100
2468400
2468700
2469000
2469300
ORA
genes
gene1303
intron_gene1303
gene1304
intron_gene1304
ncRNA229_gene1305
NCAS0A12490
NCAS0A12490
NCAS0A12500
(h)
cov. for
cov. rev
2694129
2696390
genes
2693700
2694000
2694300
2694600
2694900
2695200
2695500
2695800
2696100
2696400
2696700
ORA
genes
gene1436
NCAS0A13710
!

Additional file 2: Figure S1. The transcriptome reconstruction of *Naumovia castellii* obtained using ORA. Histograms reporting the size distribution of the 5' and 3'-UTR size of *N. castellii* **(a-b)** and *Saccharomyces cerevisiae* S288c (at 6 g/l) **(c-d)**. Notice the small 5'-UTR size of *N. castellii* (54 bp on average) in comparison to *S. cerevisiae* (81 bp on average). Analysis of the data allowed the identification of numerous introns in the 5'-end of the genes (frequently in the 5'-UTR) (red rectangles). Some of these introns were not previously predicted. **(e)** **(h)** Analysis of the 5'-UTR size revealed some genes having an incorrect prediction of the start codon (red arrows). In **(e-h)** the coverage in the forward and reverse strands determined using RNA-seq are indicated with "cov. for" and "cov. rev", the transcriptome prediction obtained using ORA is indicated with "ORA" and the gene prediction obtained from ncbi database is indicates as "genes".
