# Supplementary material for: Transcriptome structure variability in Saccharomyces cerevisiae strains determined with a newly developed assembly software
Source: BMC Genomics. 2014 Dec 1;15(1):1045. doi: 10.1186/1471-2164-15-1045 (PMC4302112; doi:10.1186/1471-2164-15-1045)
Supplement: Supplementary file 3 — Additional file 3: Figure S2: Selected results of the transcript structure obtained for the six strains under analysis. Genes reported belong to the GO categories “sulfur compound metabolic process” and “sterol metabolic process” that are described in the main text. Genes YDR213W and YGL012W have highly conserved 5’-UTRs and were reported in order to show that gene expression level has little influence on the transcript structure prediction. The 5’-end of the transcript is on the left for genes encoded on the forward strand and on the right for genes encoded on the reverse strand. 5’-UTR is indicated by a small arrow. (PDF 7 MB) [file 12864_2014_6763_MOESM3_ESM.pdf]

sulfur compound metabolic process

YAL012W; *CYS3*

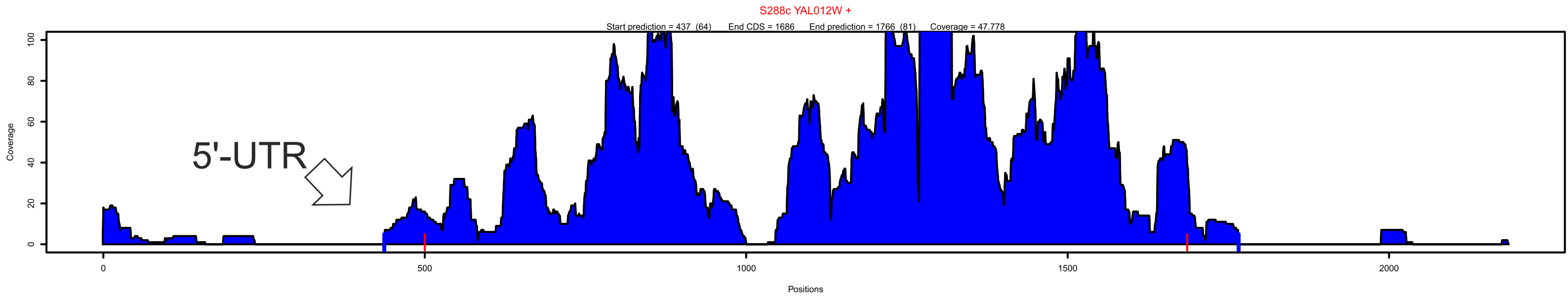

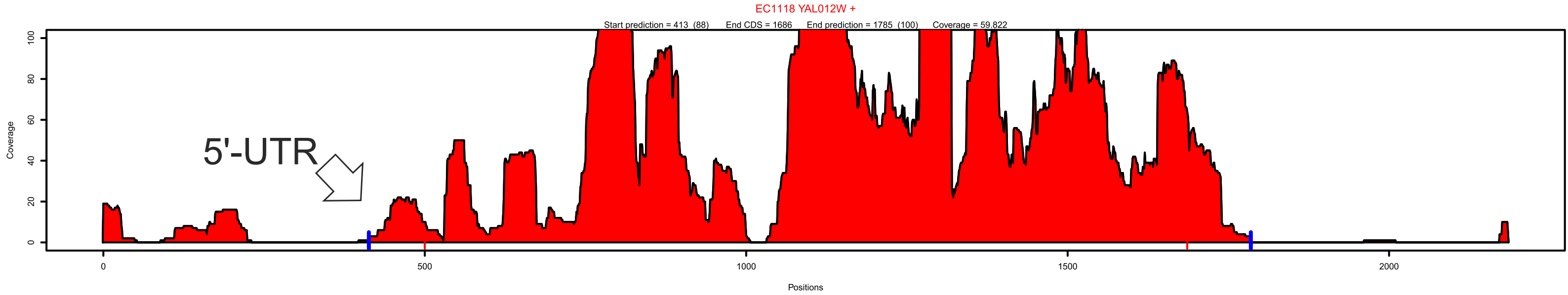

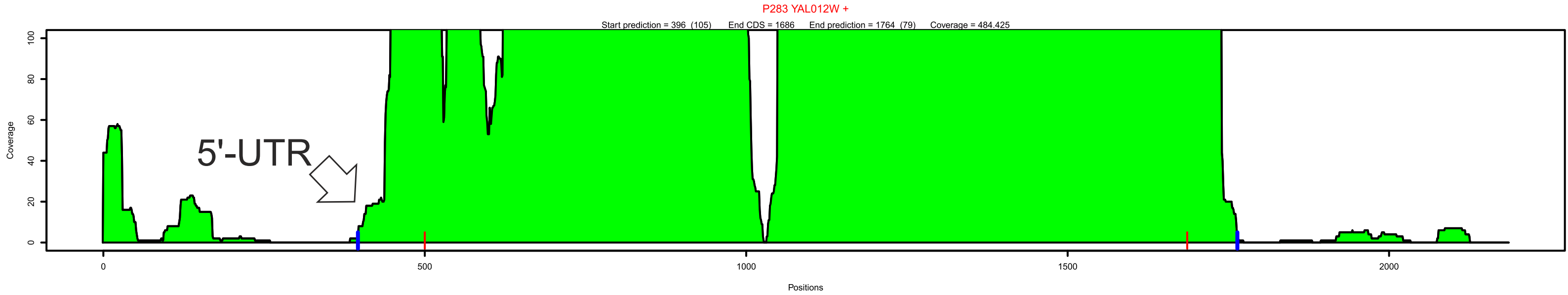

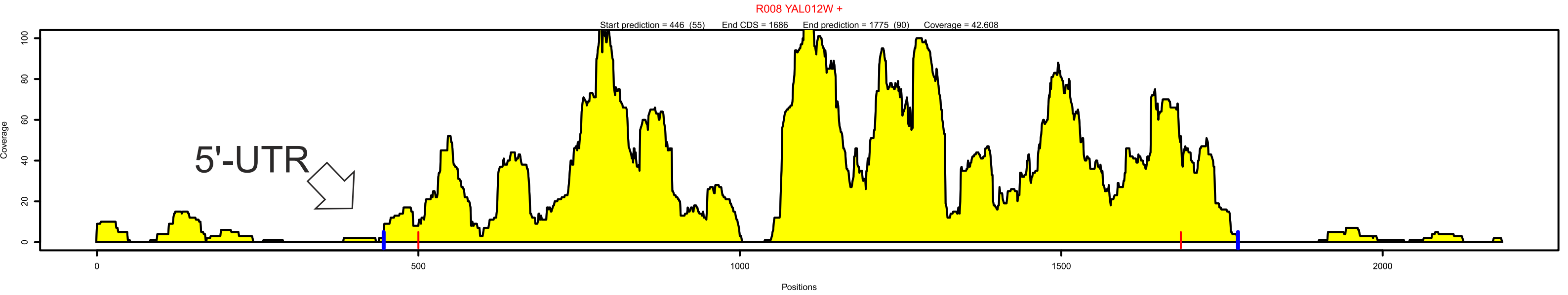

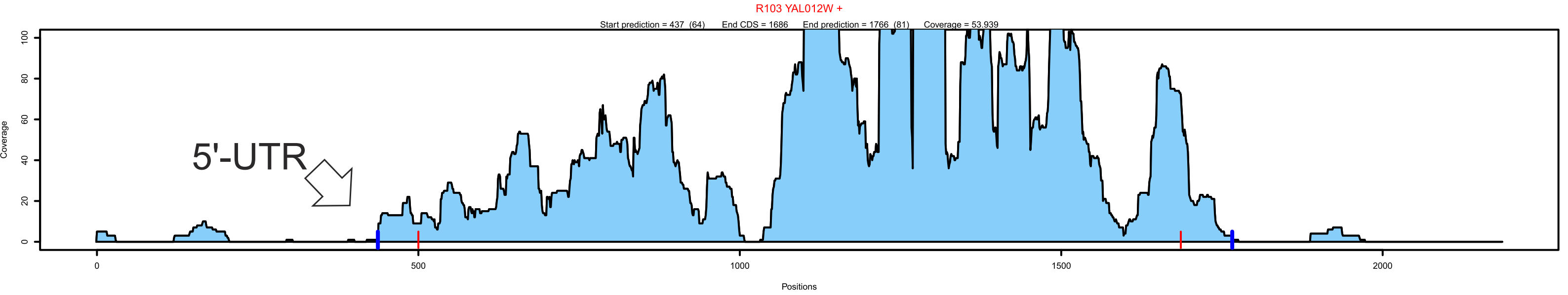

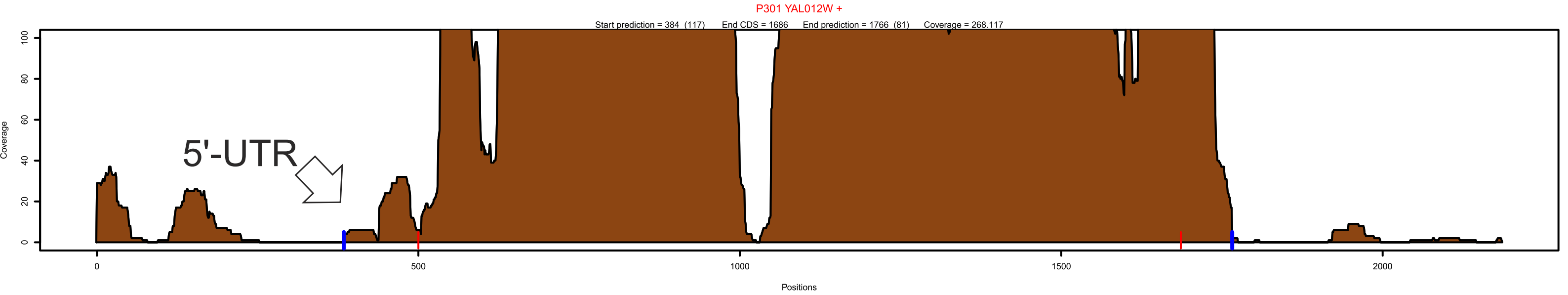

sulfur compound metabolic process

YBR084W; *MIS1*

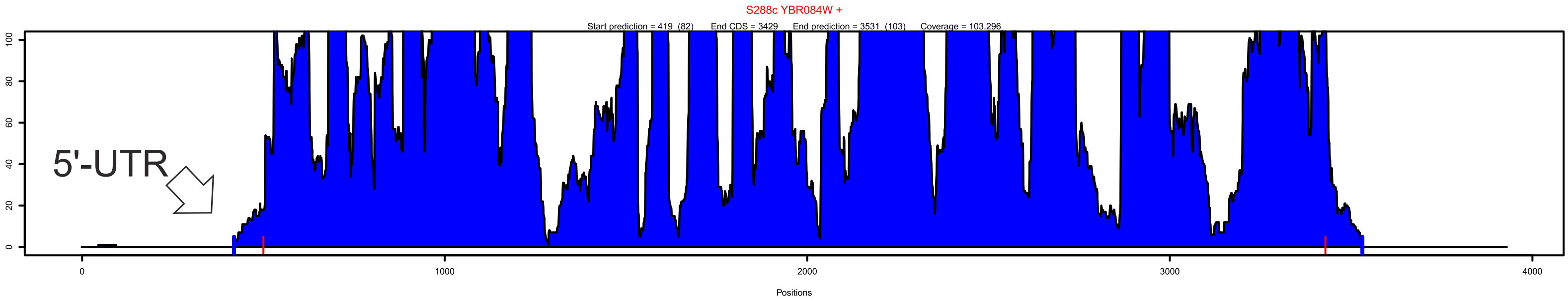

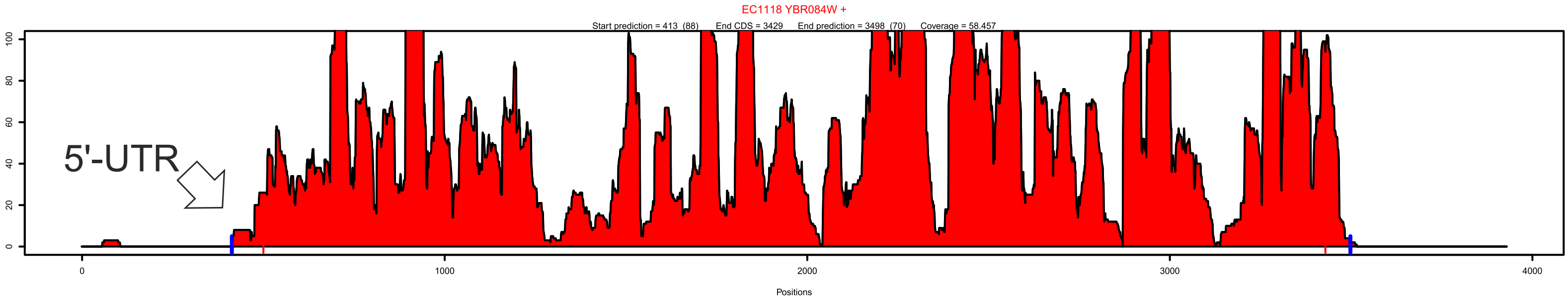

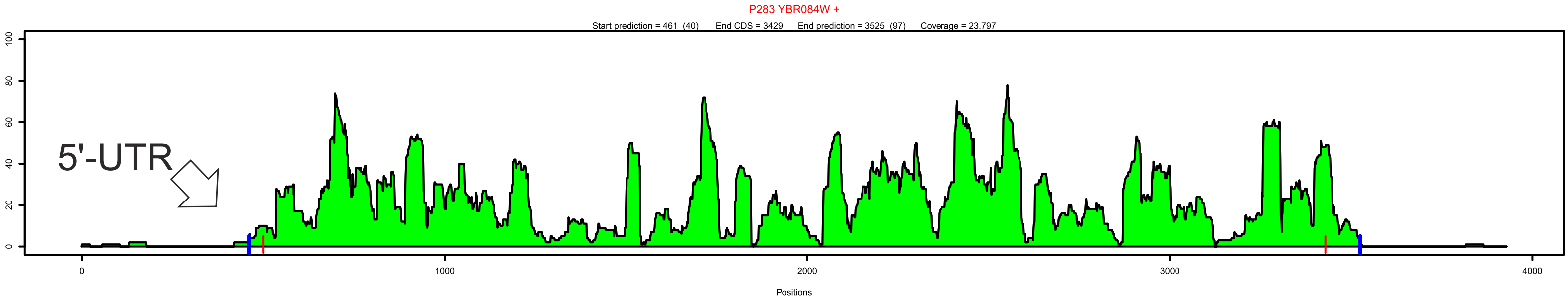

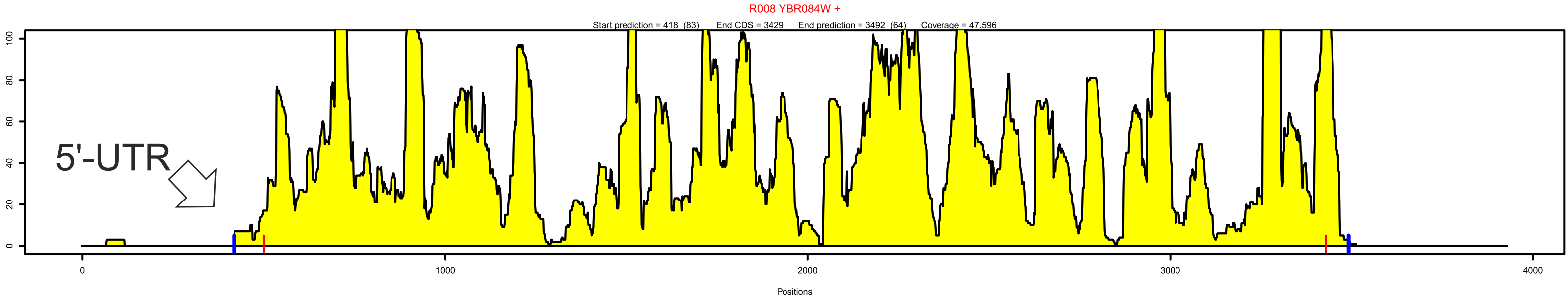

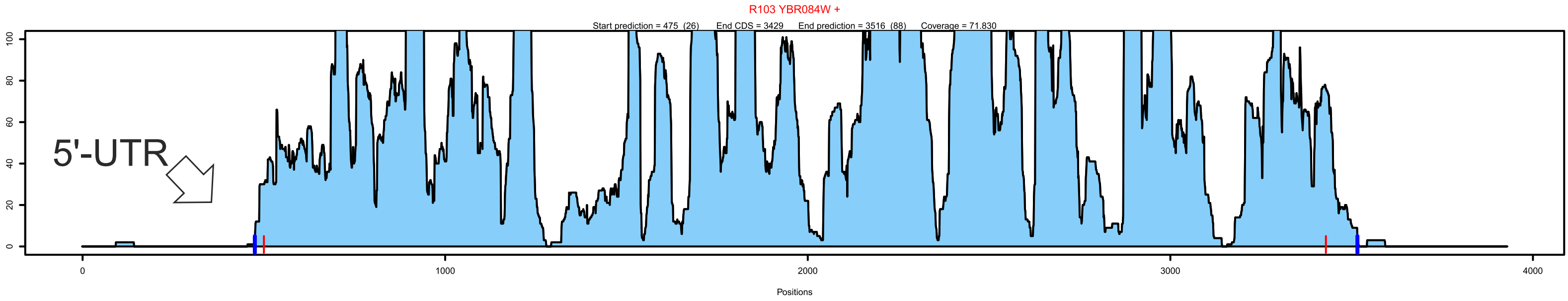

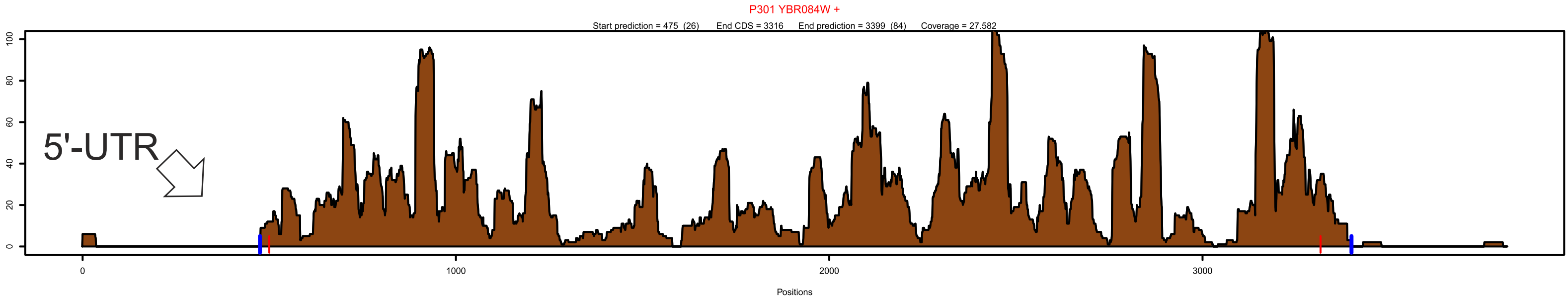

sulfur compound metabolic process

YBR240C; *THI2*

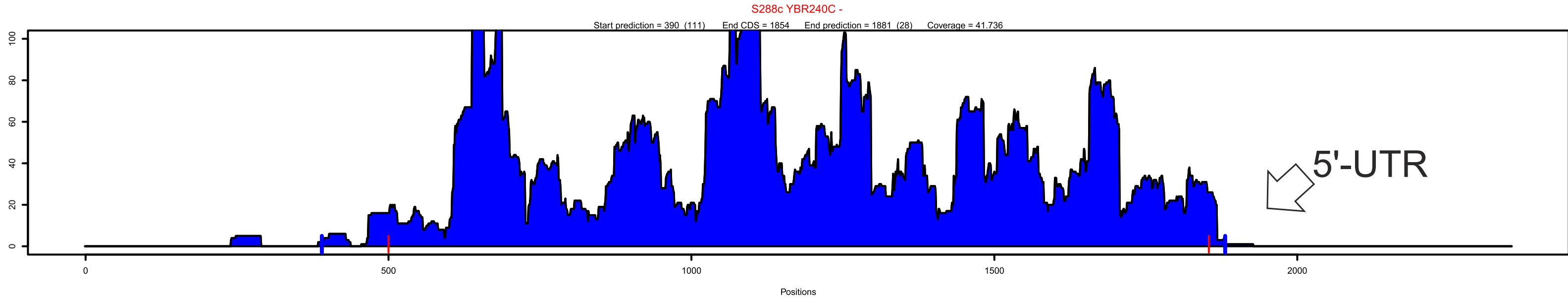

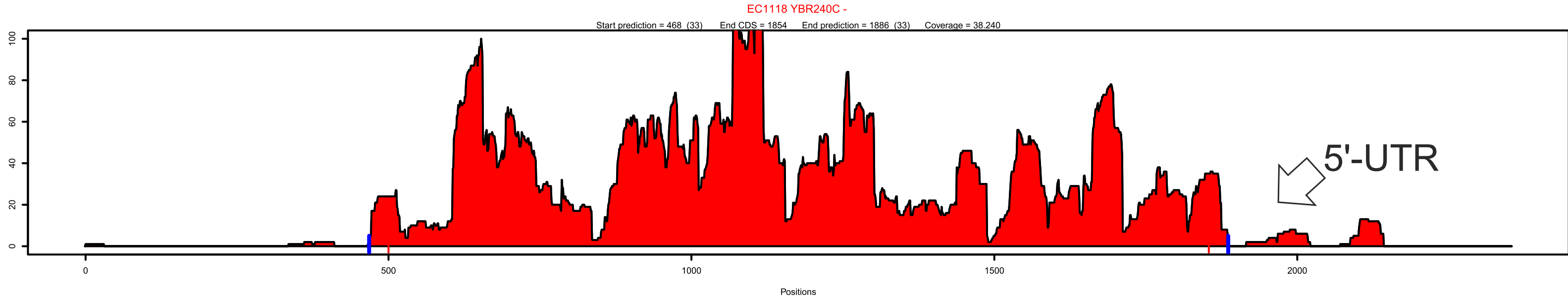

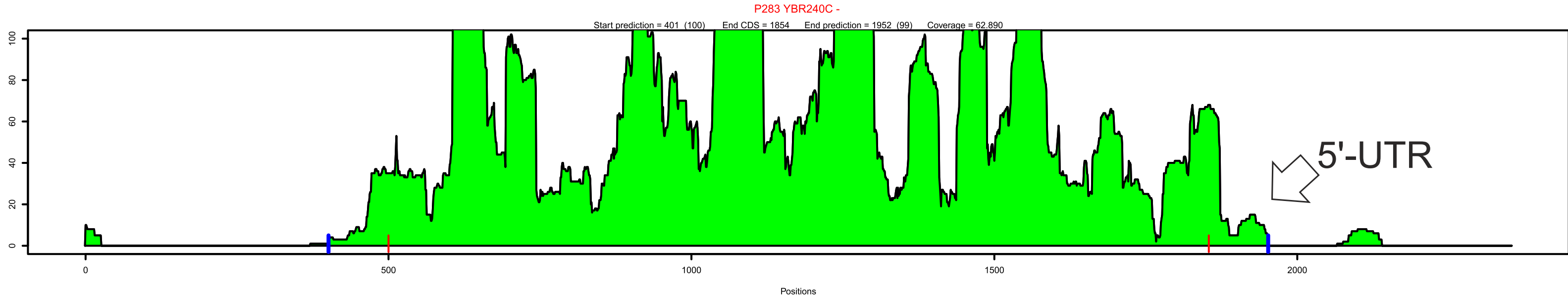

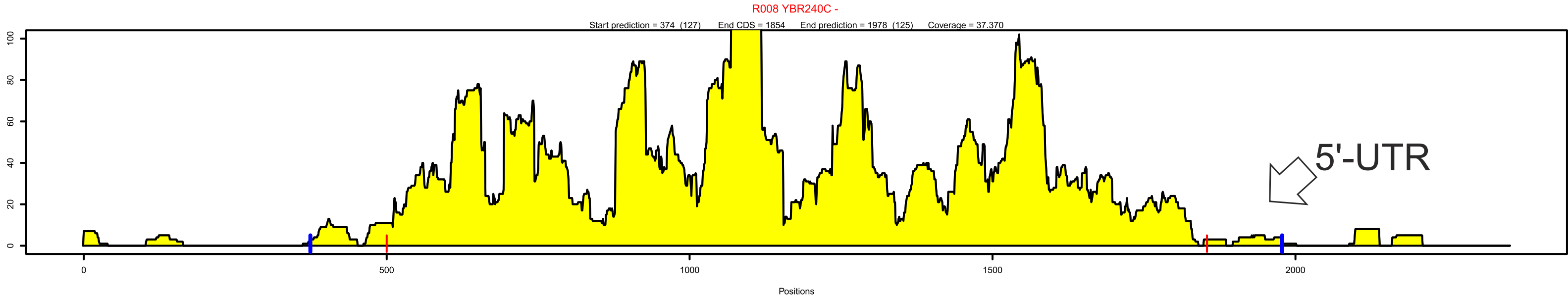

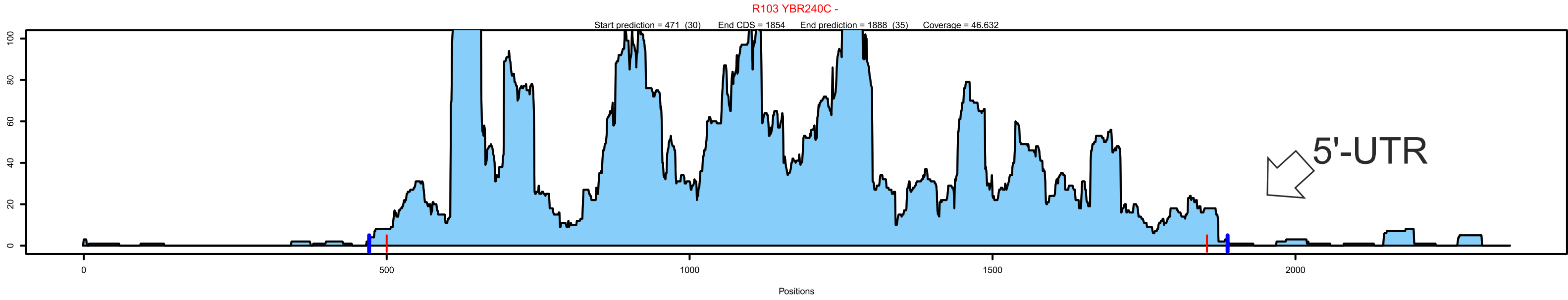

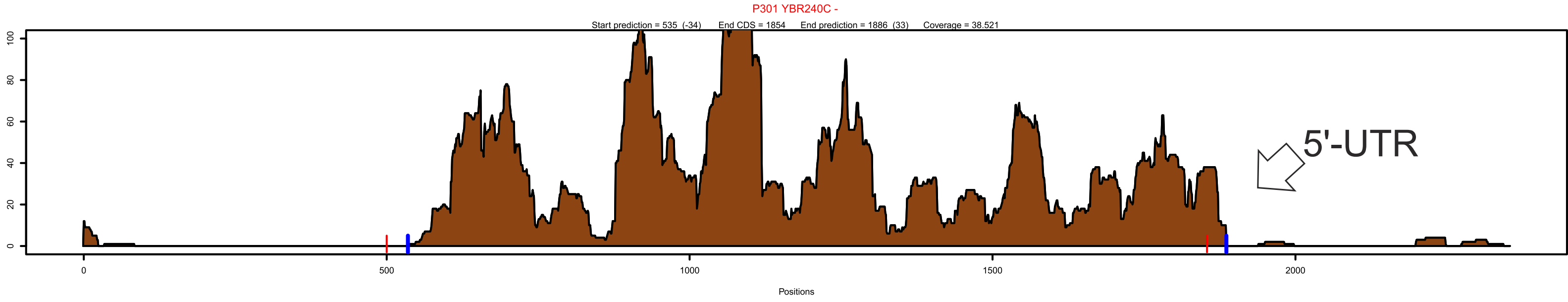

sulfur compound metabolic process

YHR208W; *BAT1*

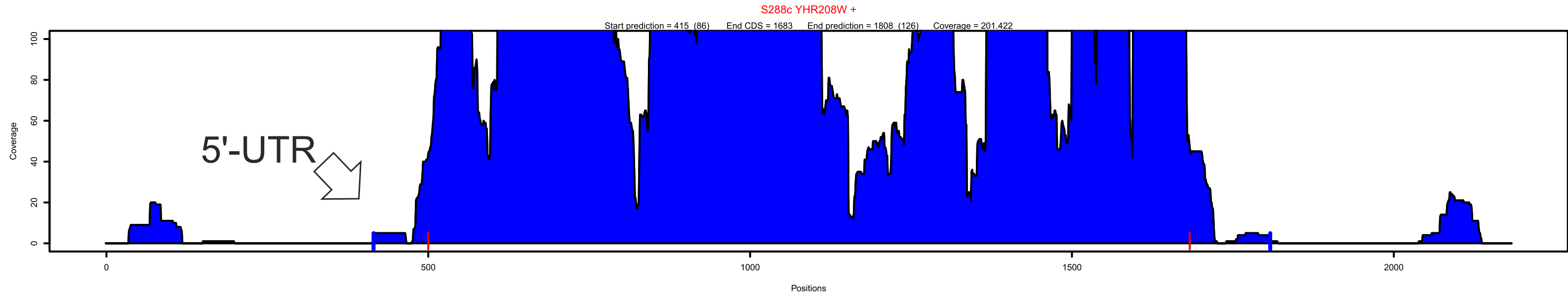

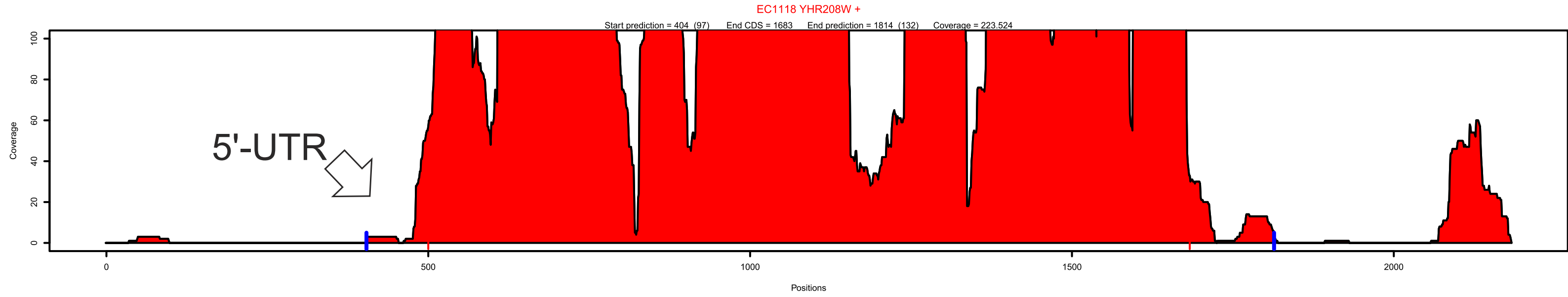

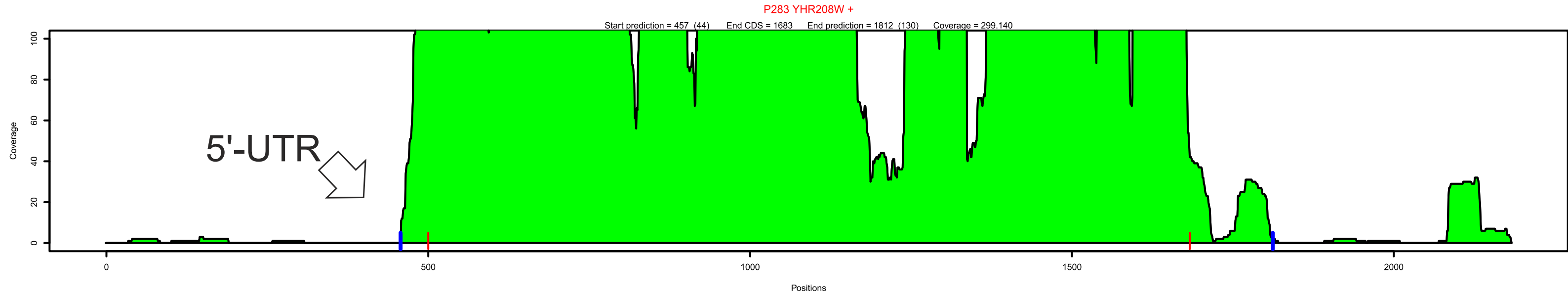

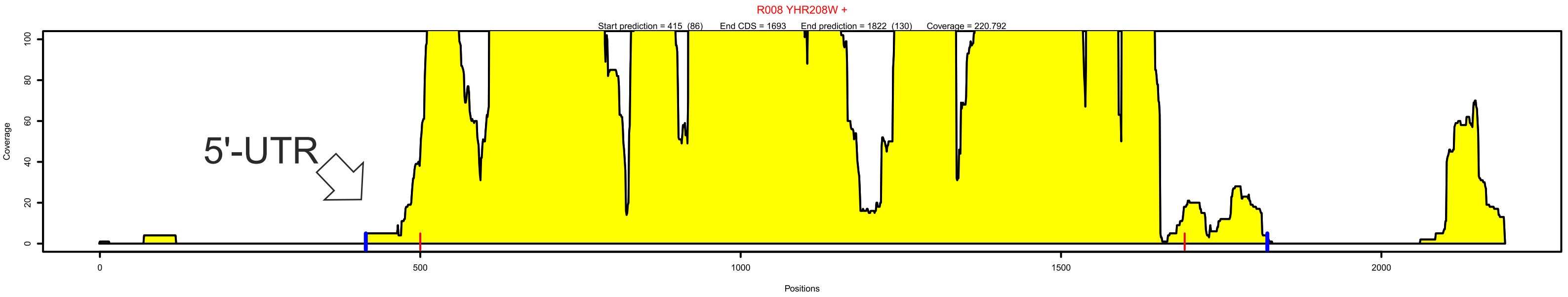

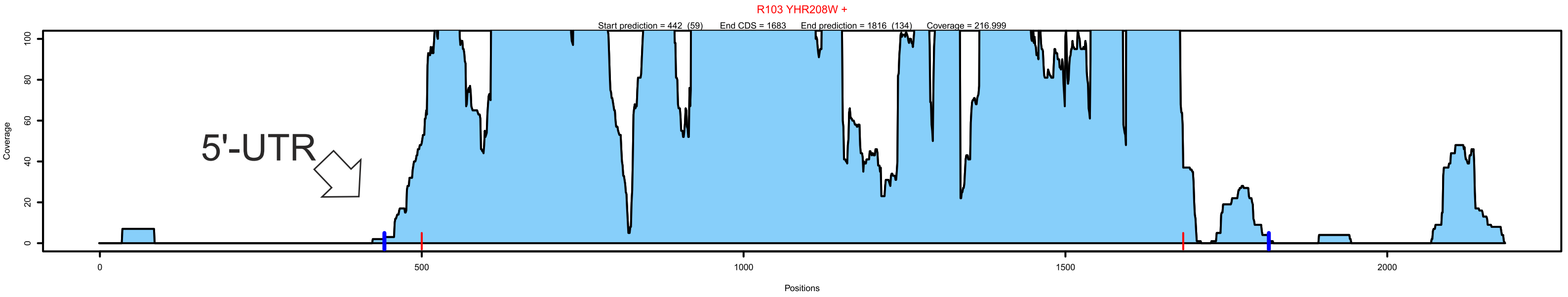

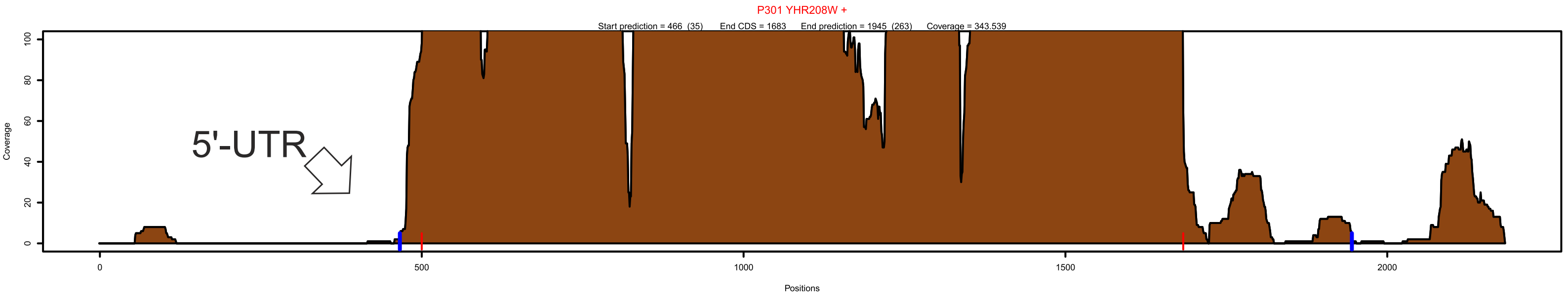

sulfur compound metabolic process

YKR069W; *MET1*

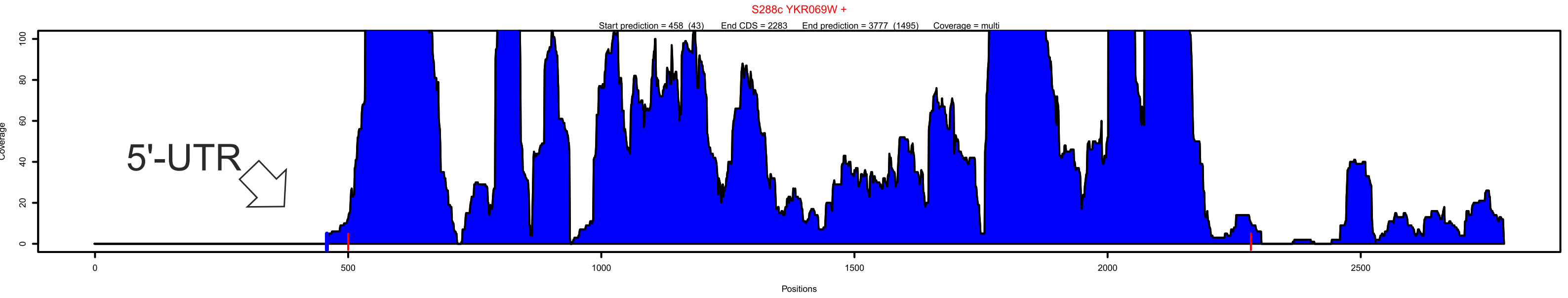

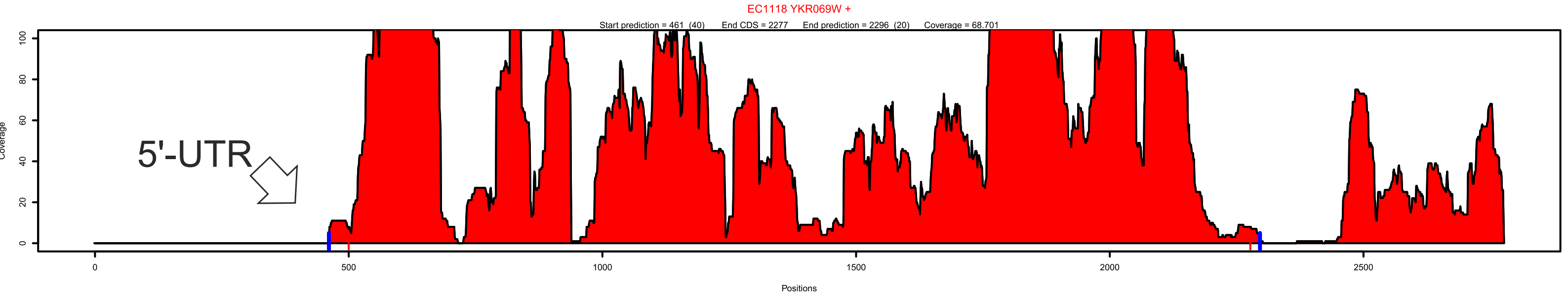

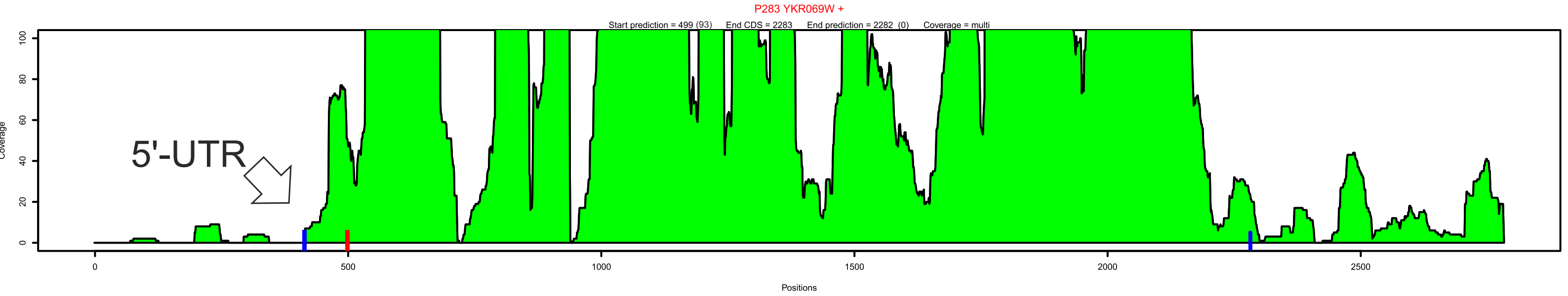

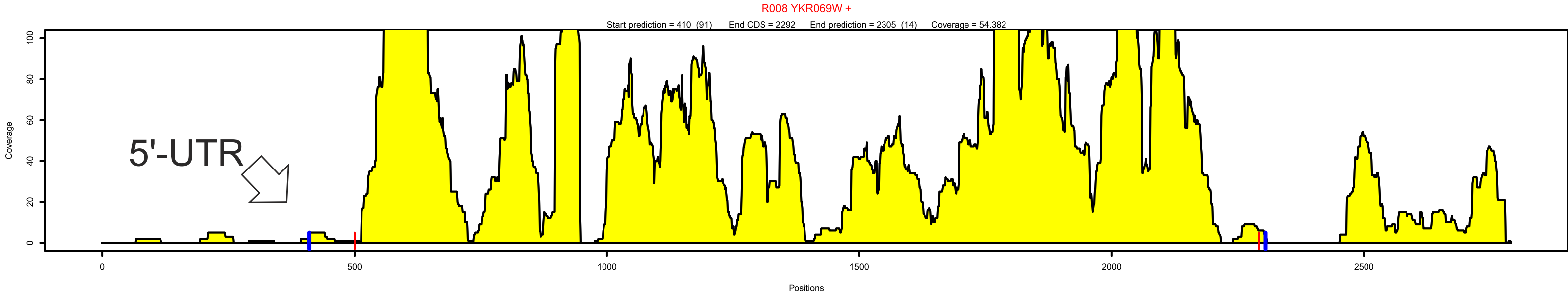

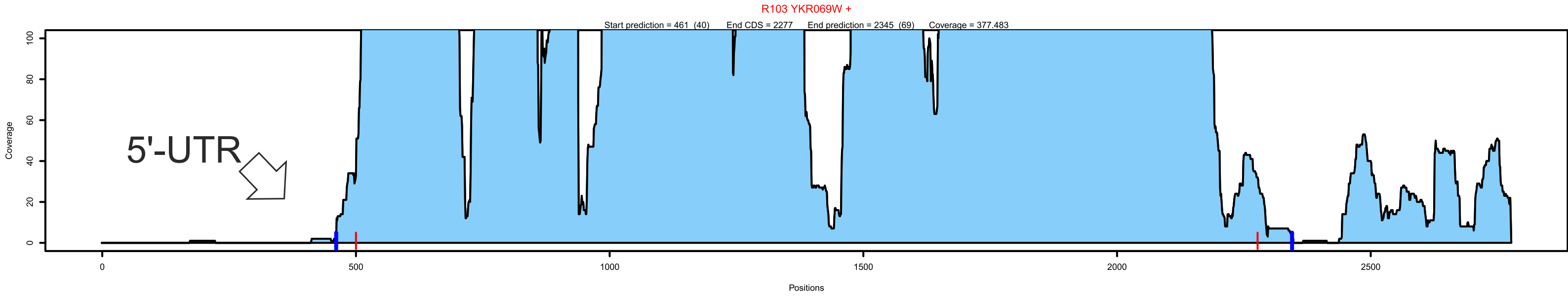

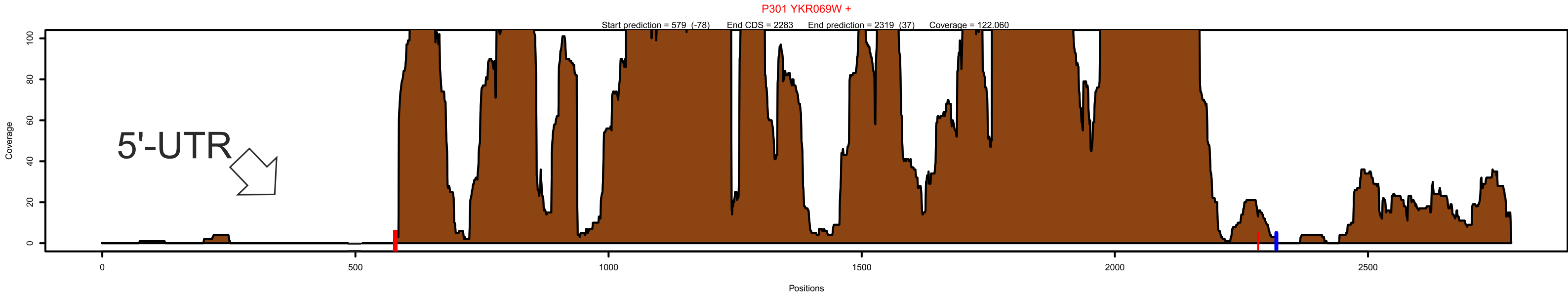

sulfur compound metabolic process

YDR272W; *GLO2*

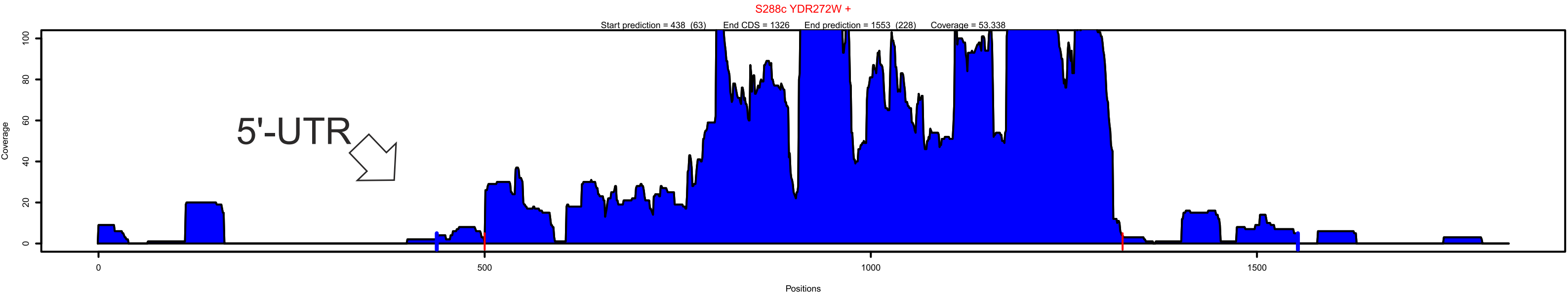

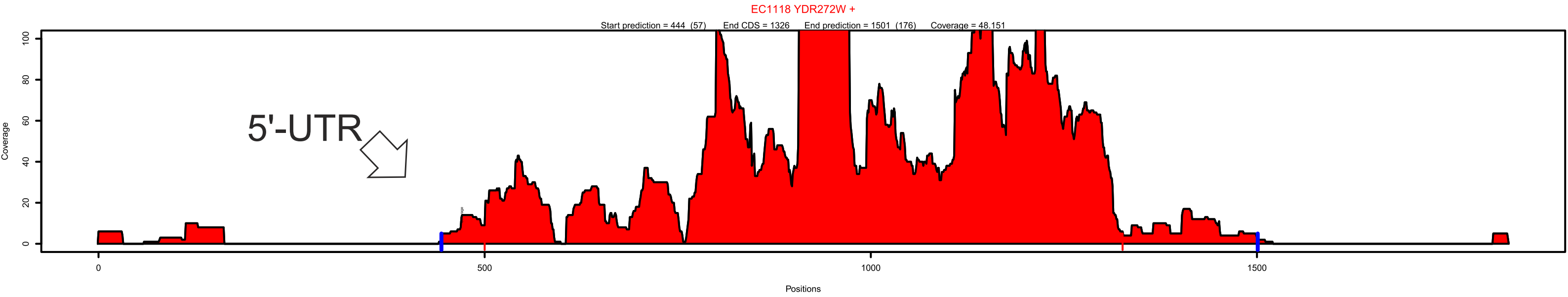

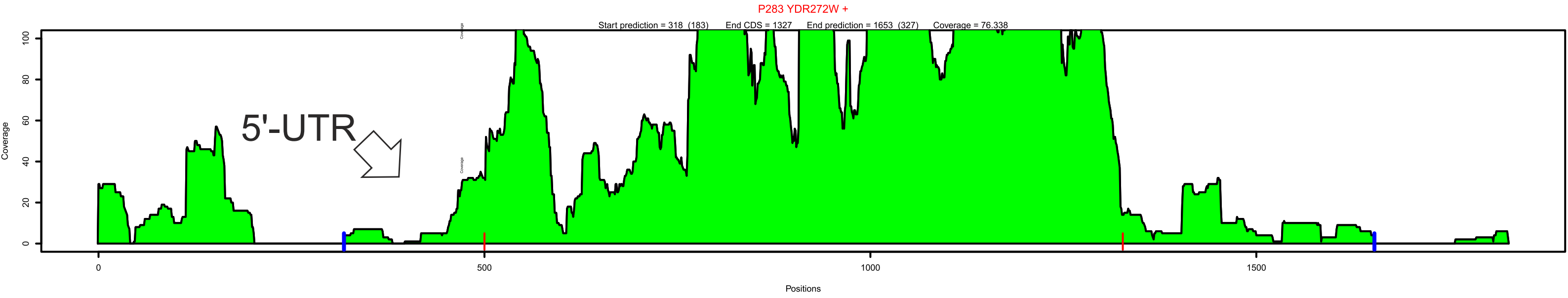

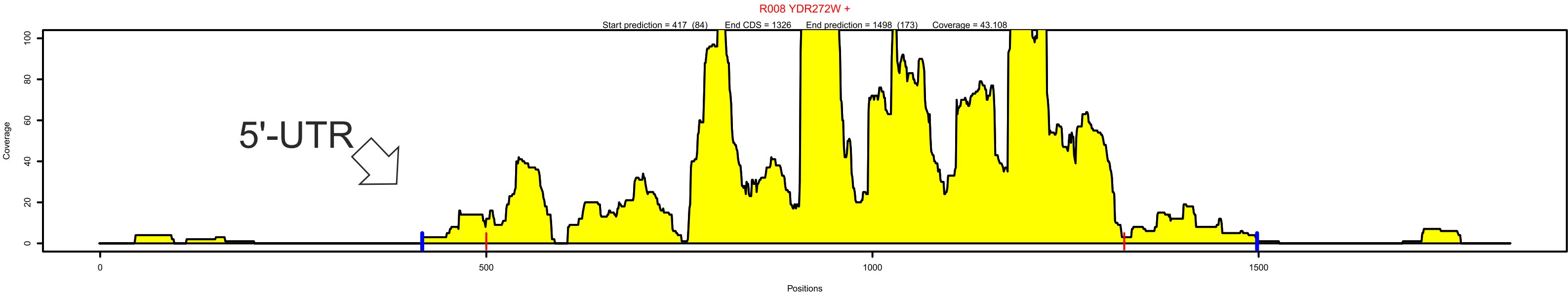

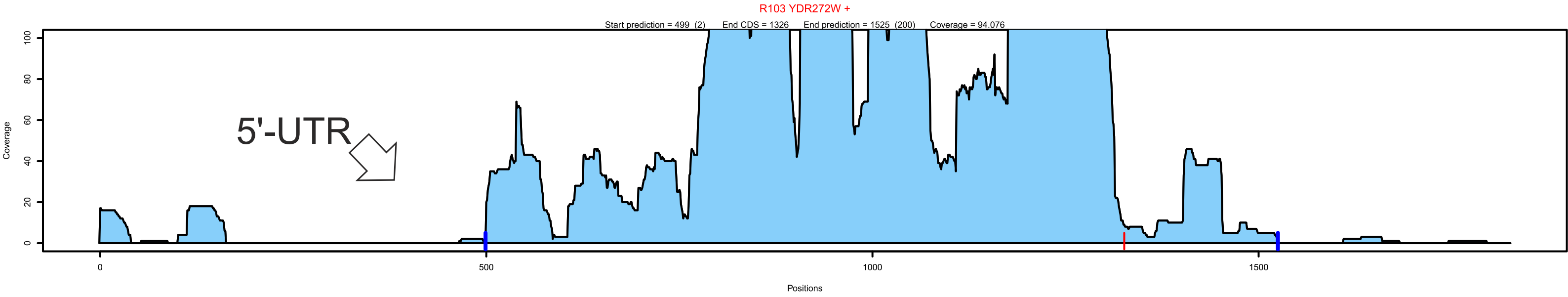

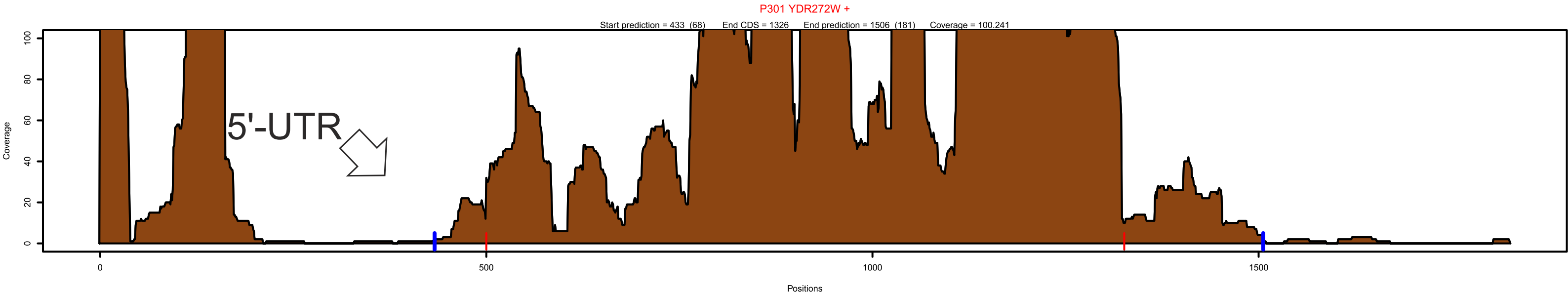

sulfur compound metabolic process

YLL058W

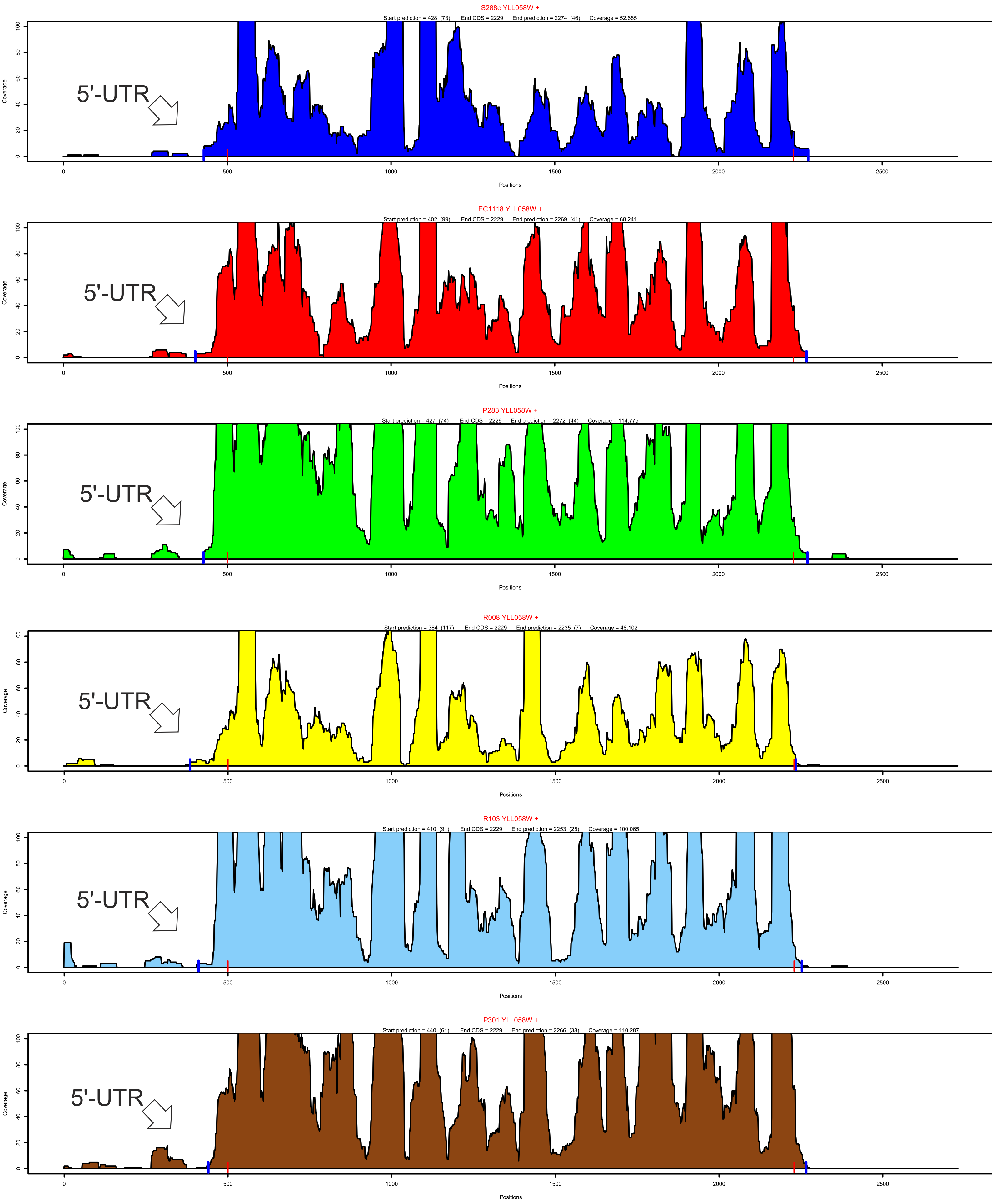

sulfur compound metabolic process

YLR180W; *SAM1*

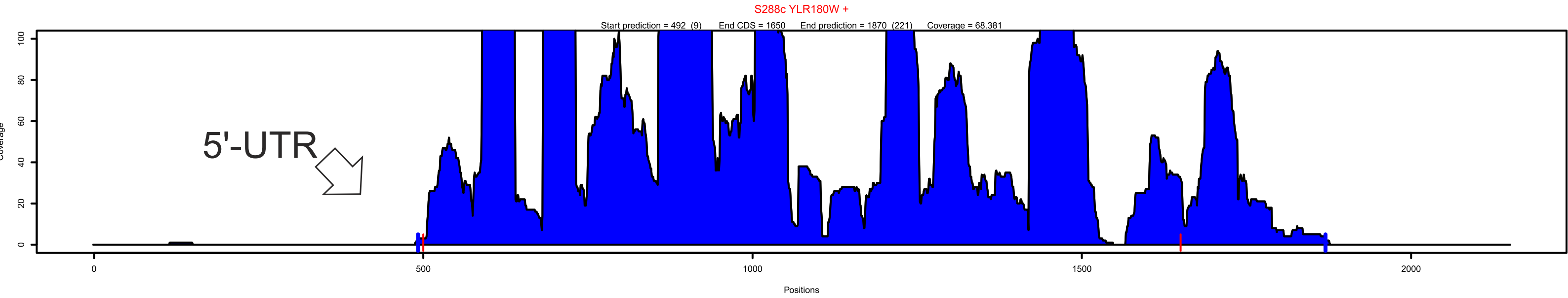

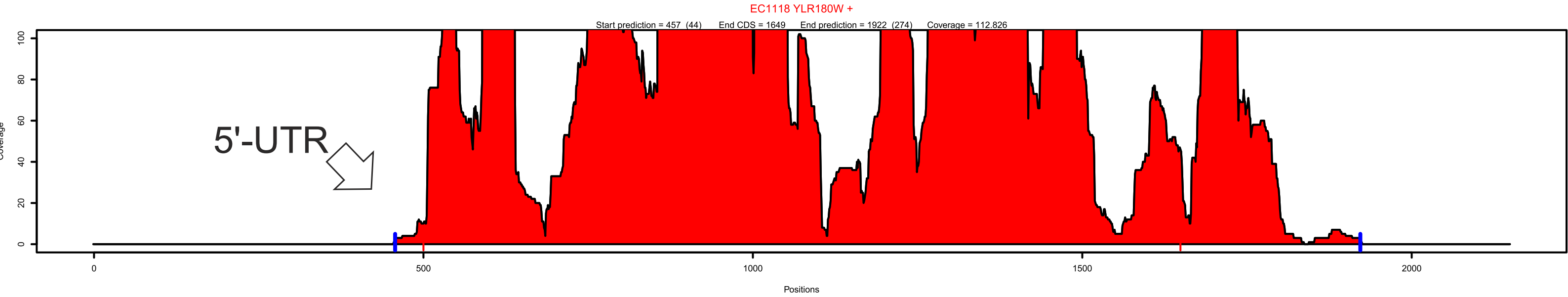

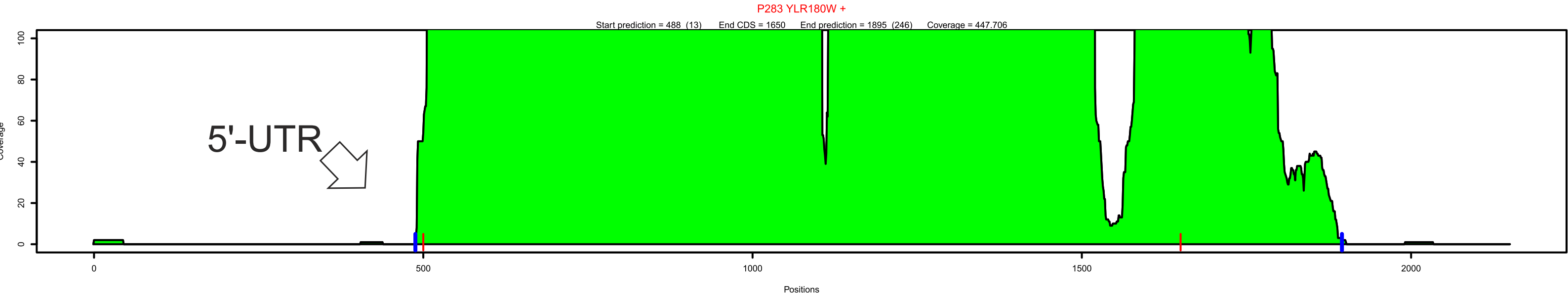

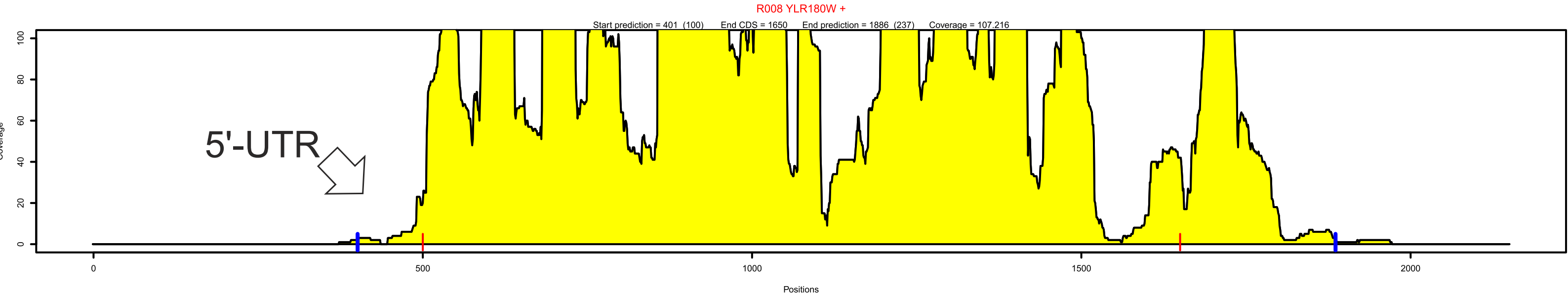

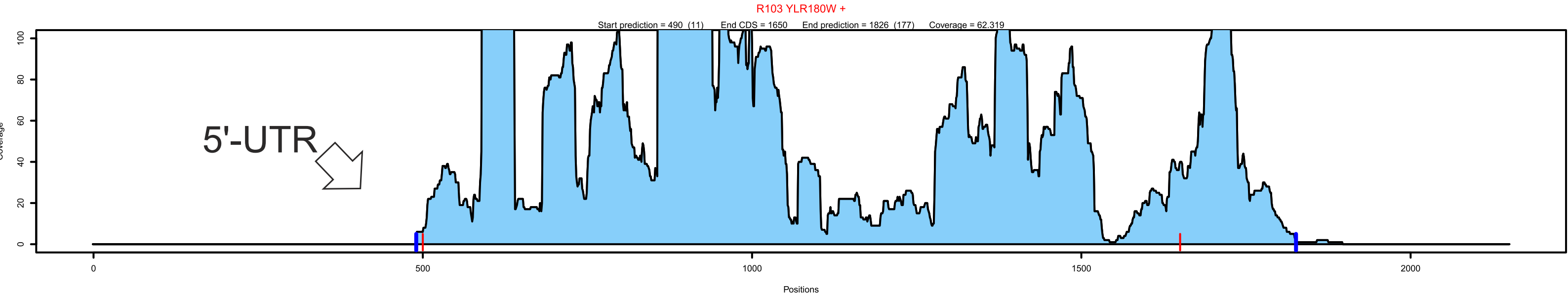

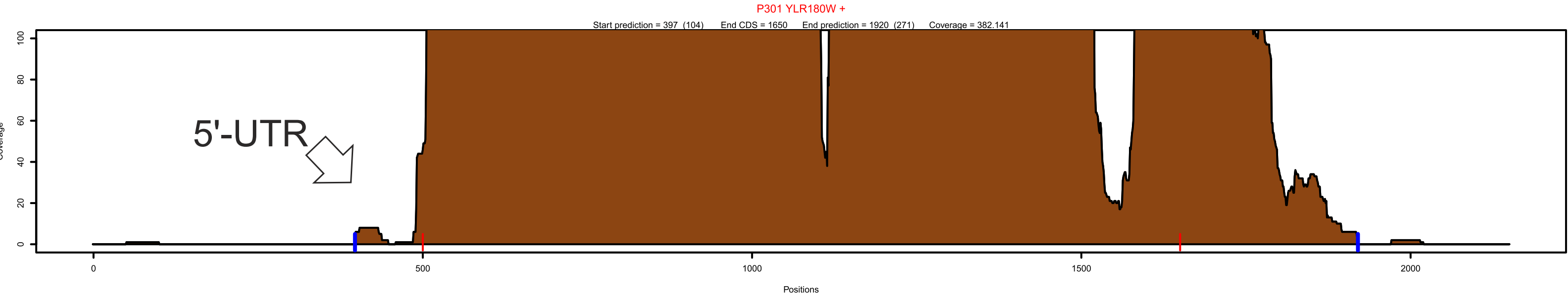

sulfur compound metabolic process

YOL049W; *GSH2*

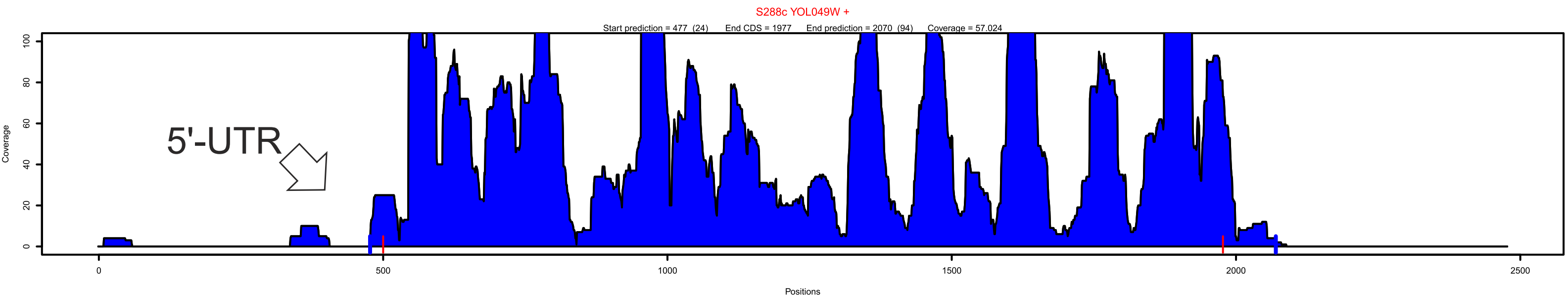

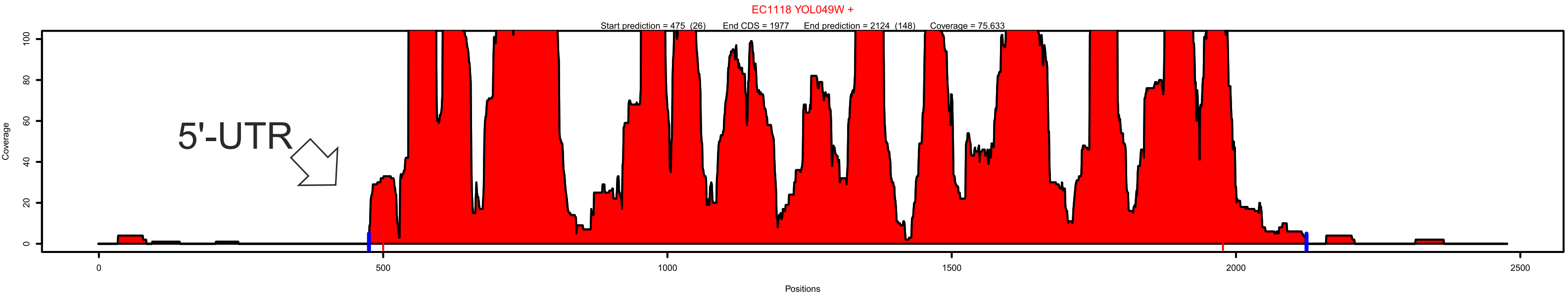

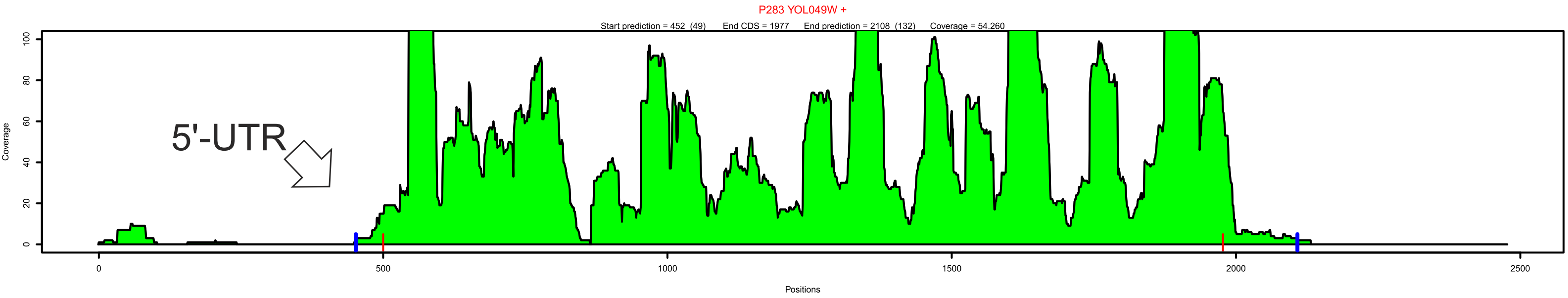

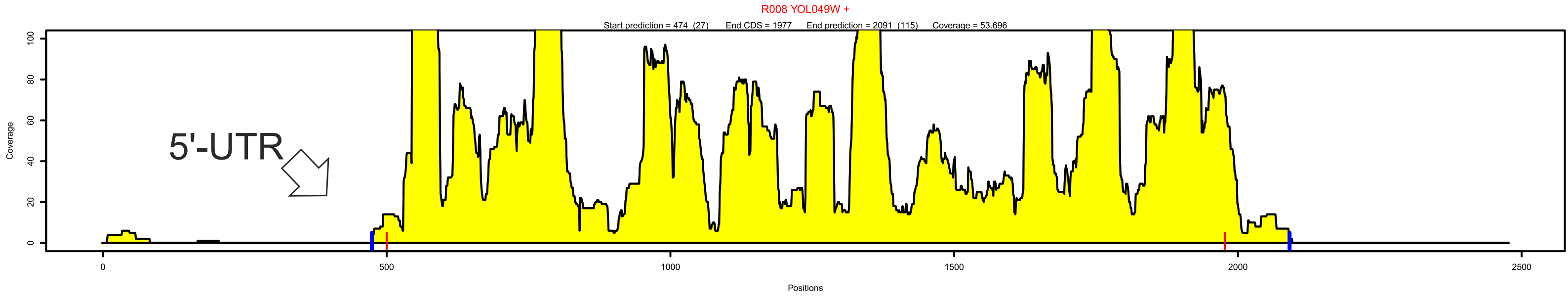

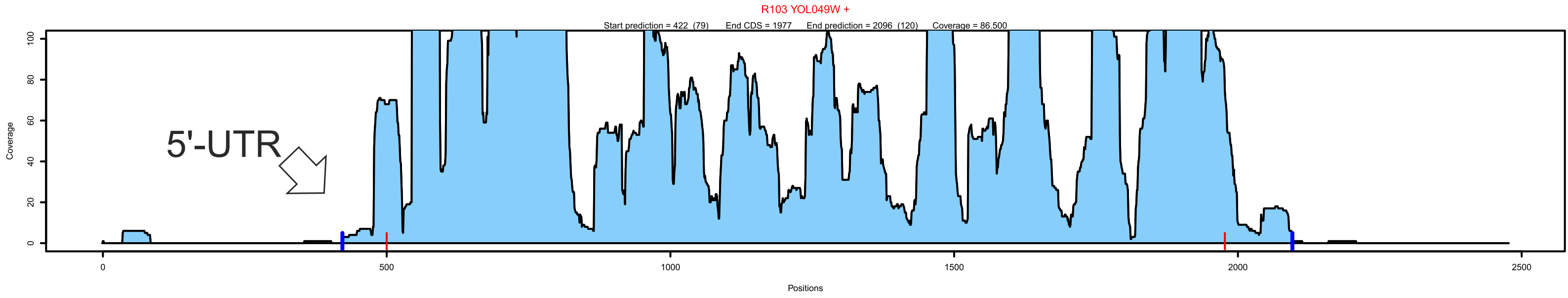

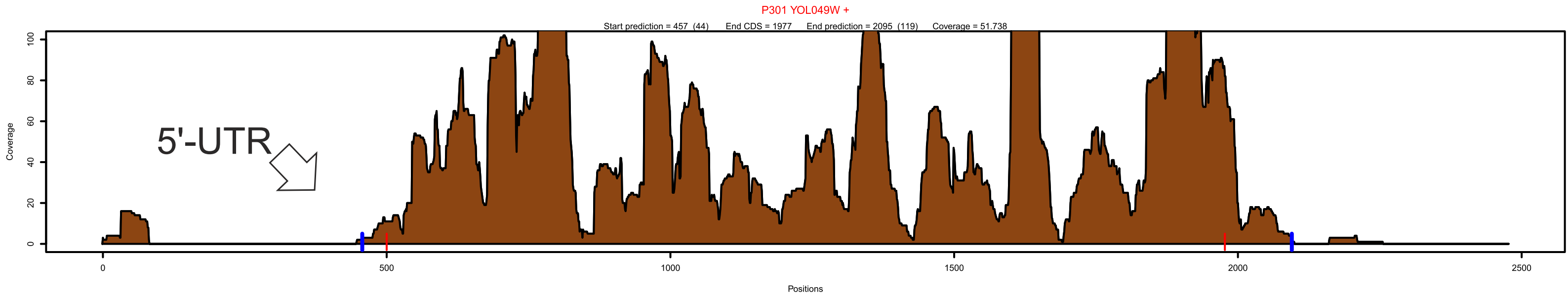

sulfur compound metabolic process

YPL214C; *THI6*

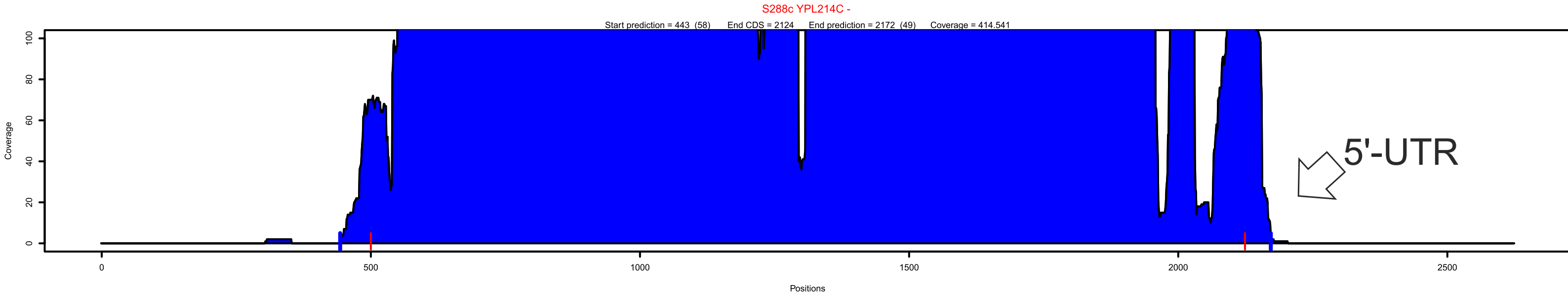

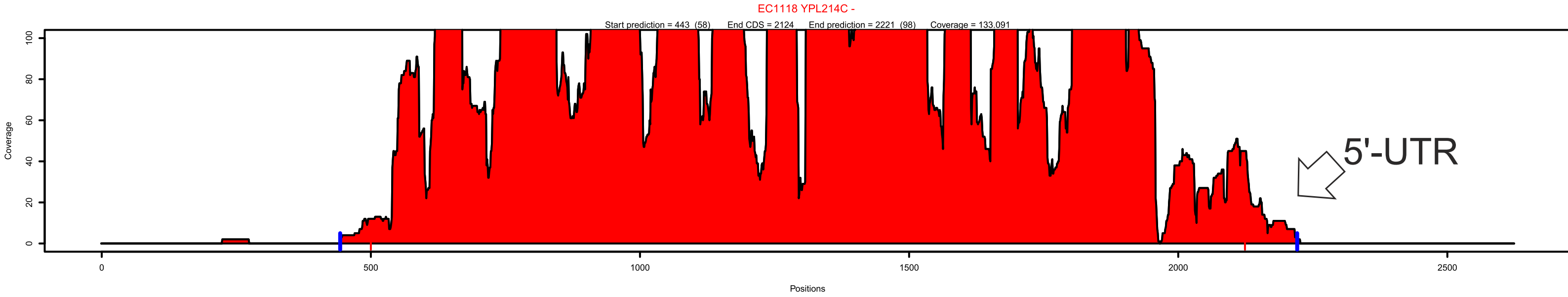

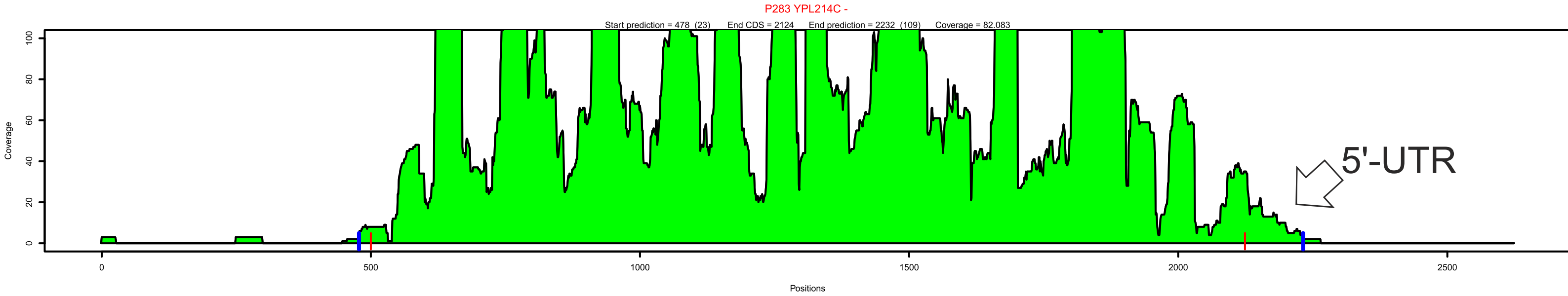

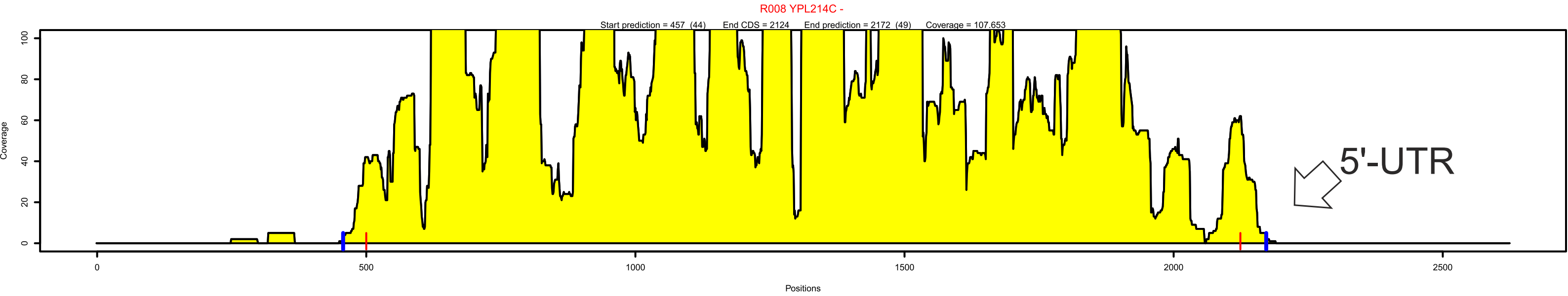

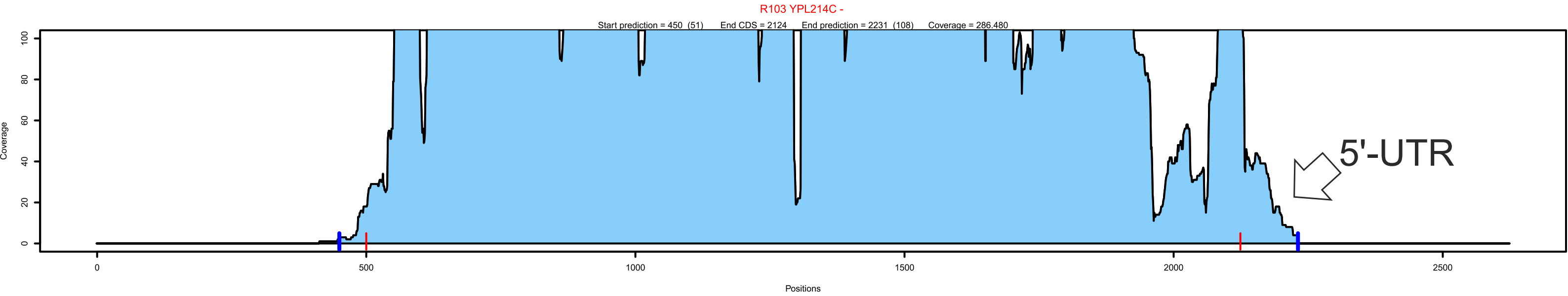

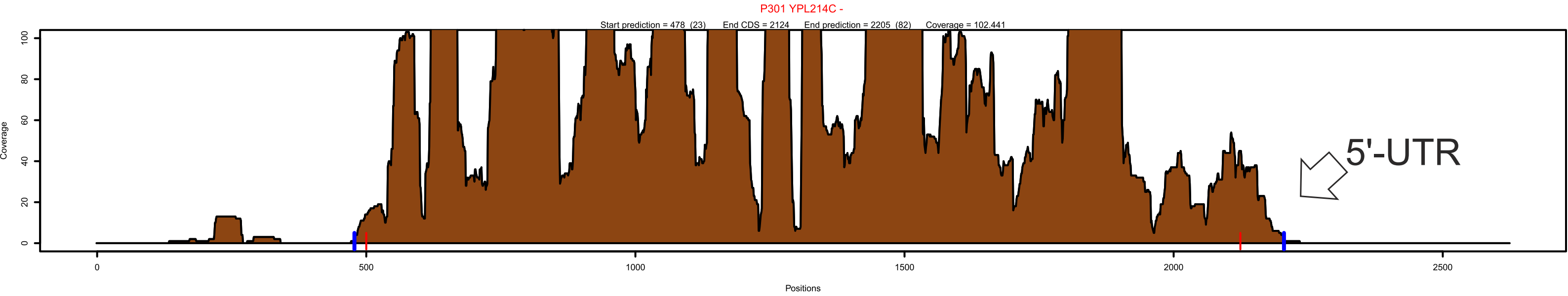

sterol metabolic process

YDR213W; *UPC2* - highly conserved 5'-UTR

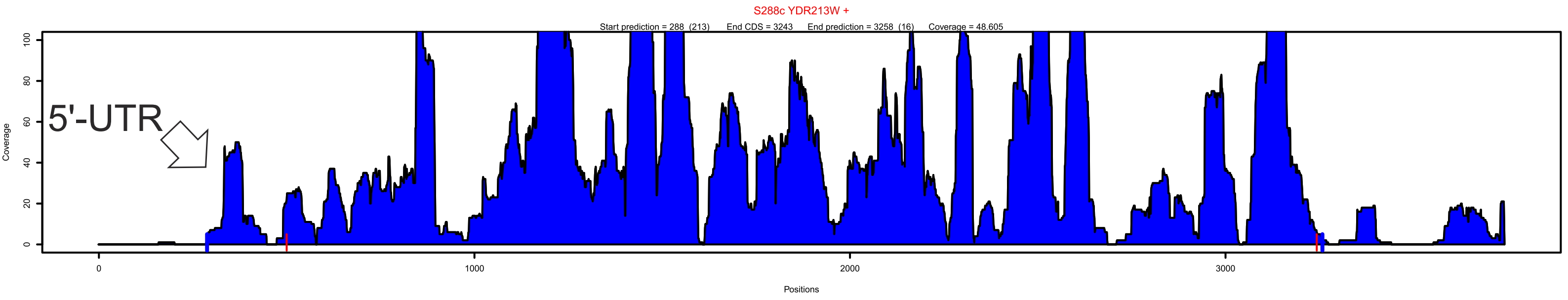

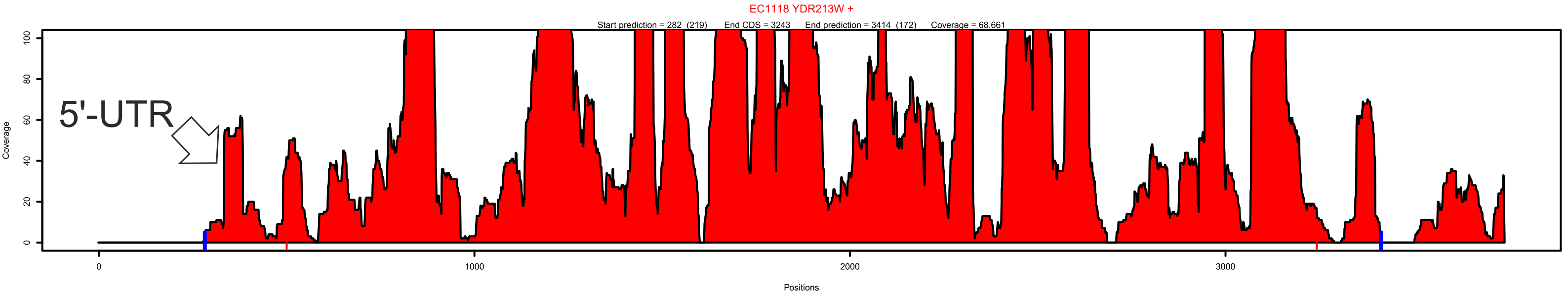

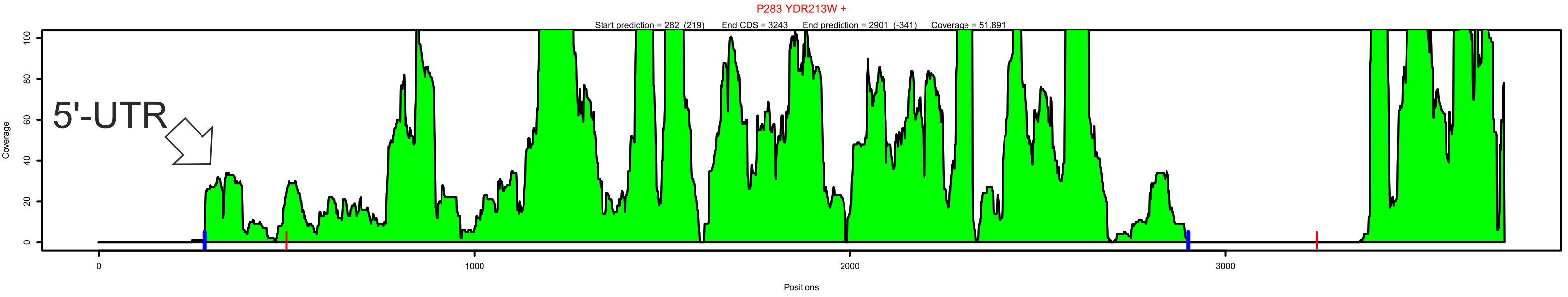

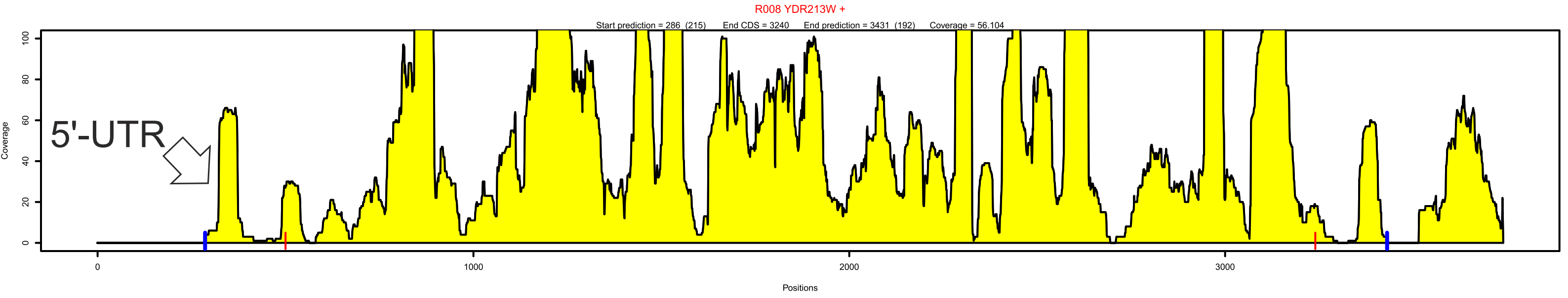

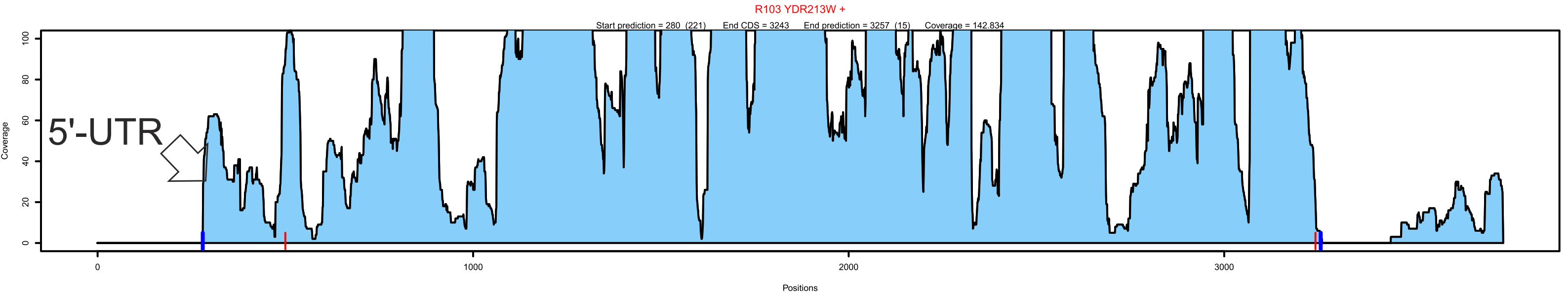

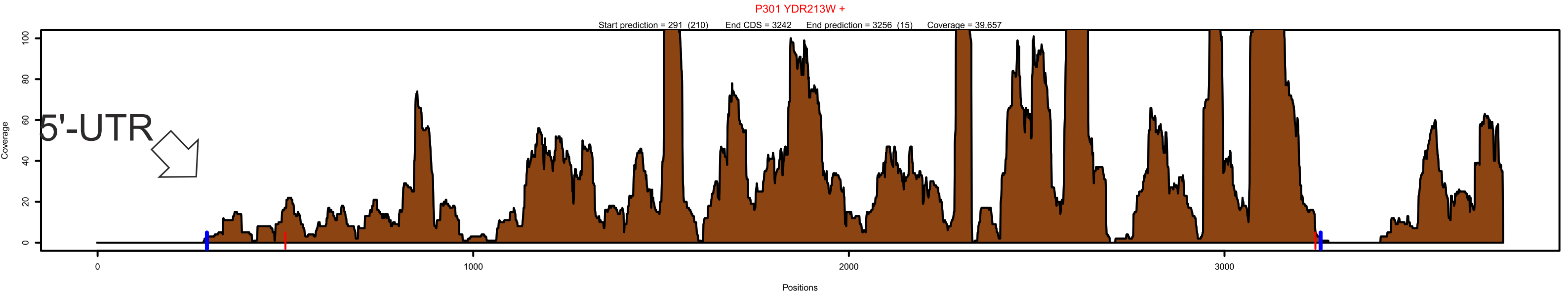

sterol metabolic process

YGL012W; *ERG4* - highly conserved 5'-UTR

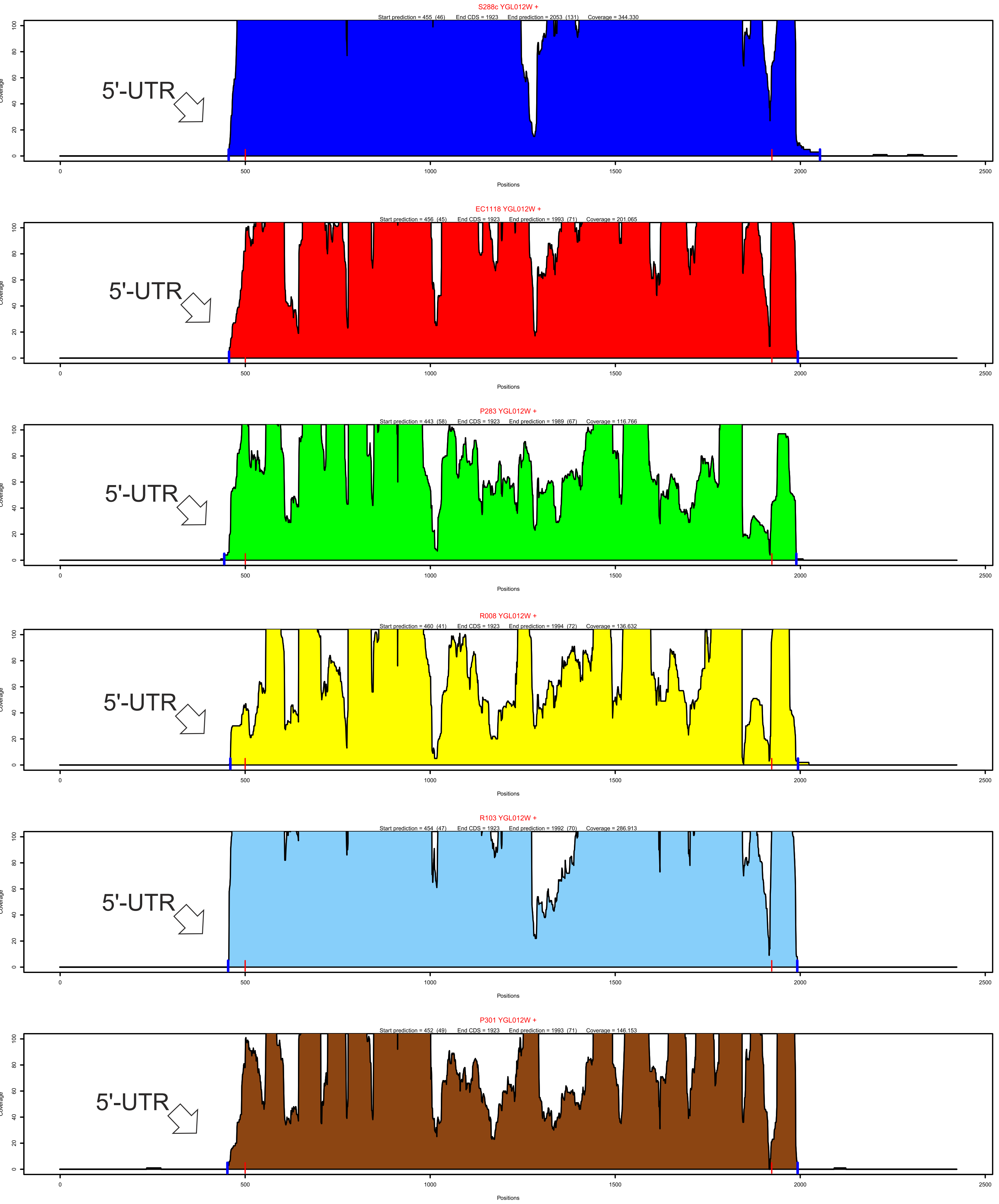

sterol metabolic process

YGR060W; *ERG25*

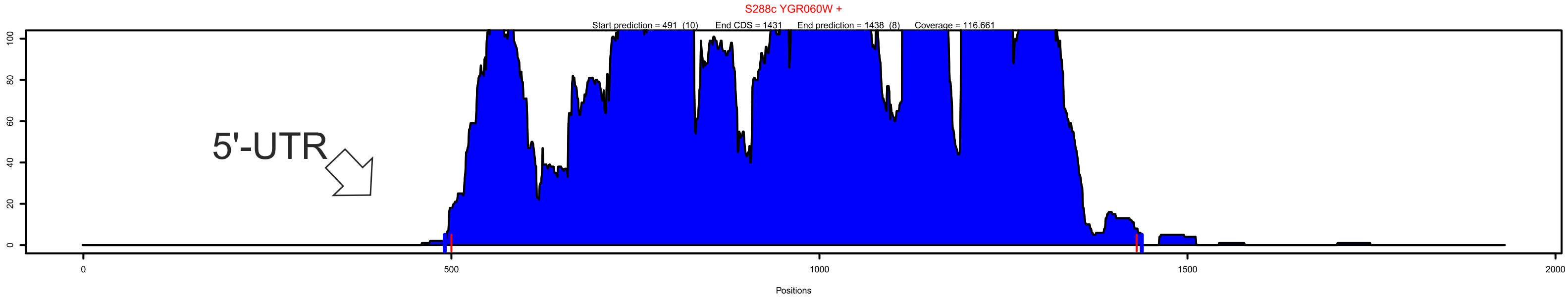

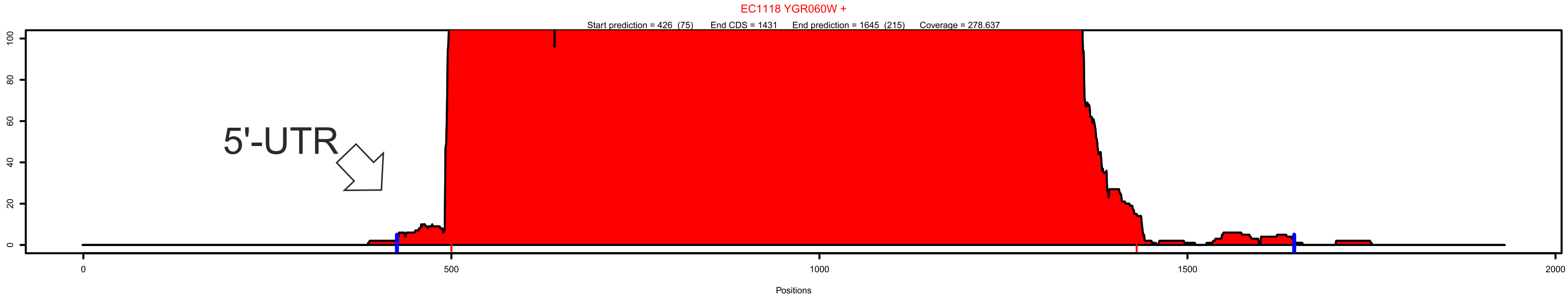

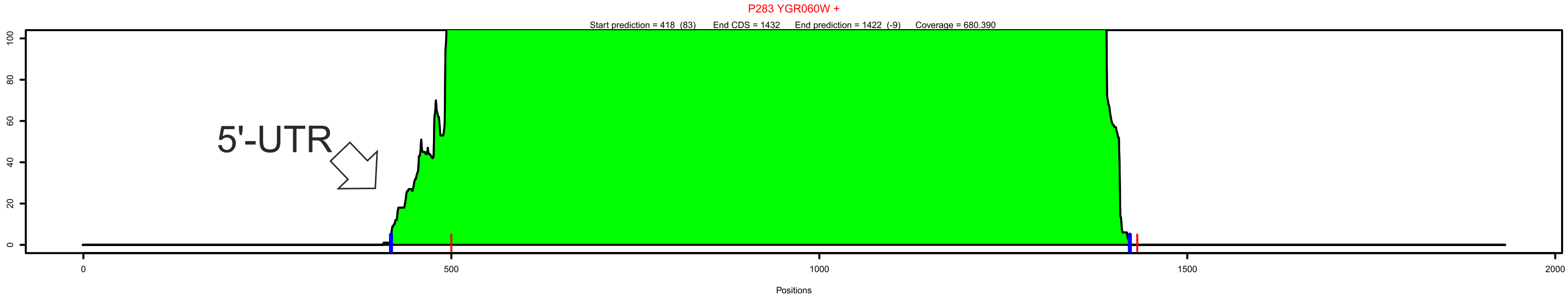

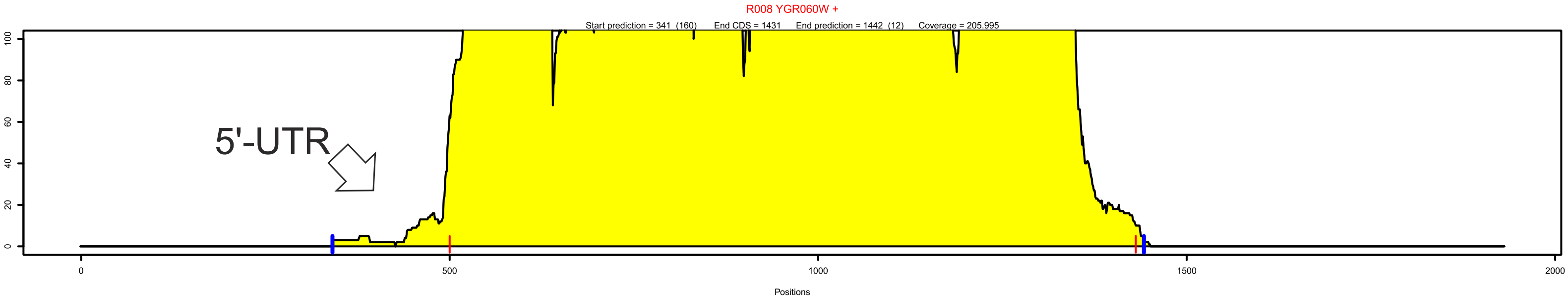

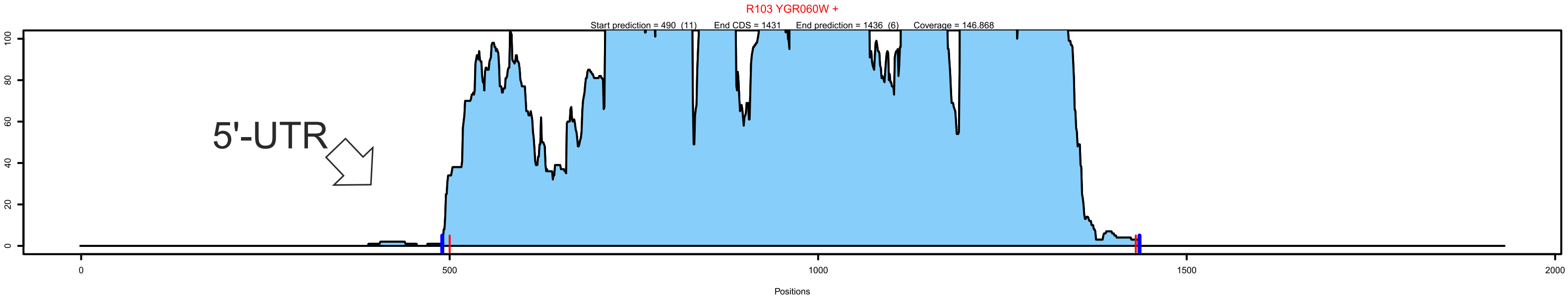

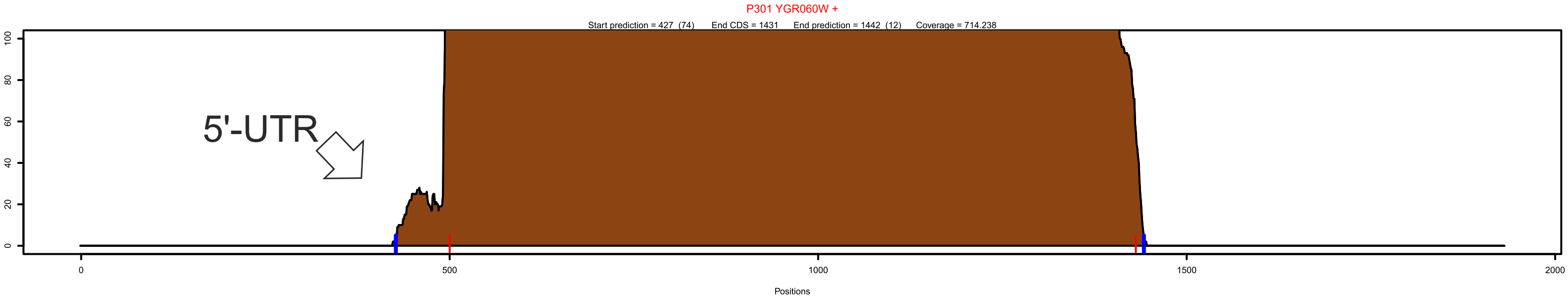

sterol metabolic process

YML008C; *ERG6*

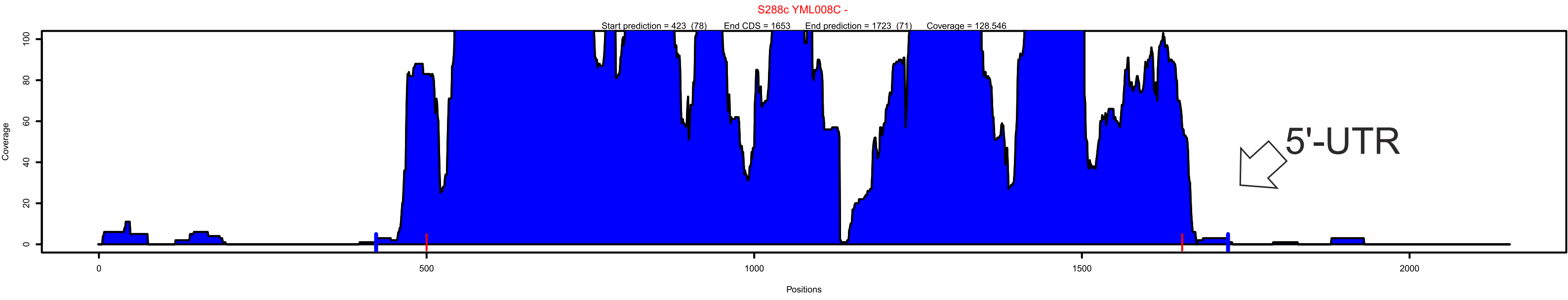

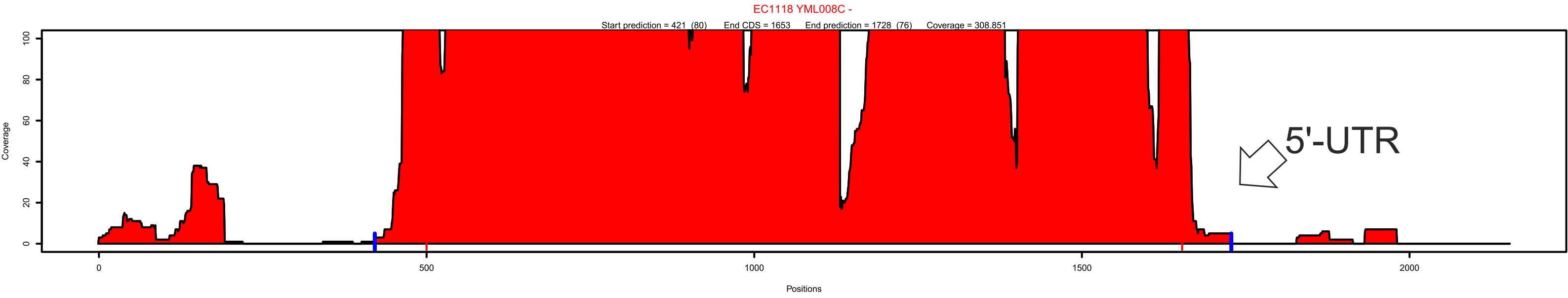

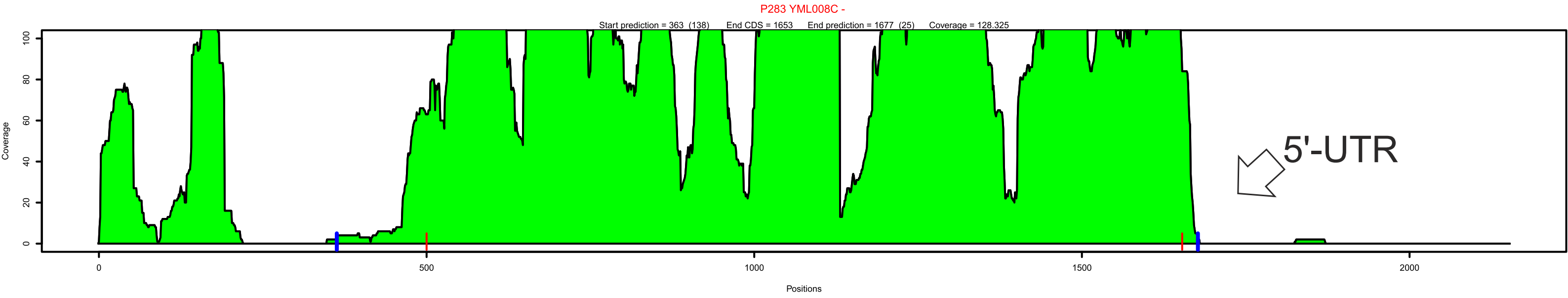

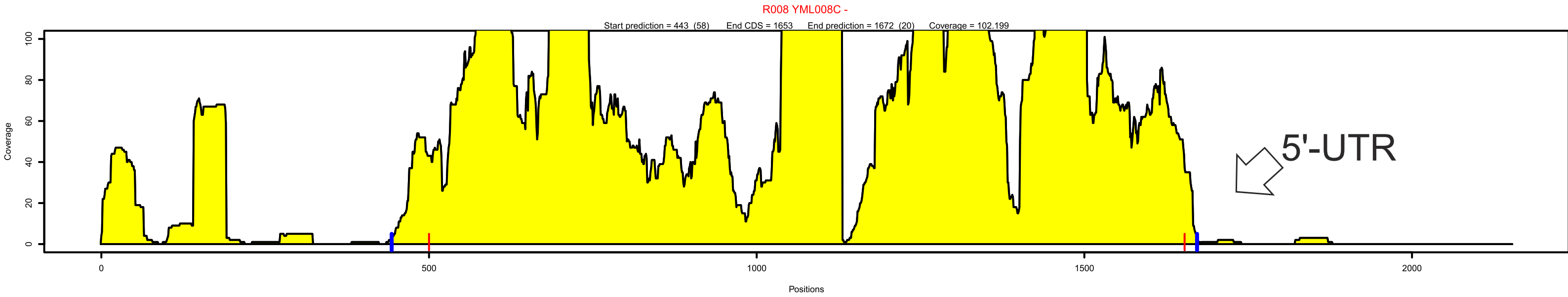

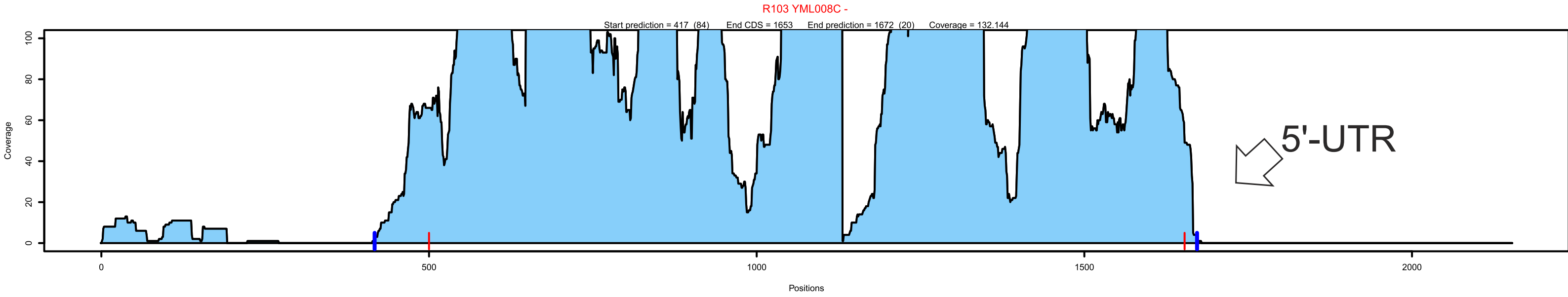

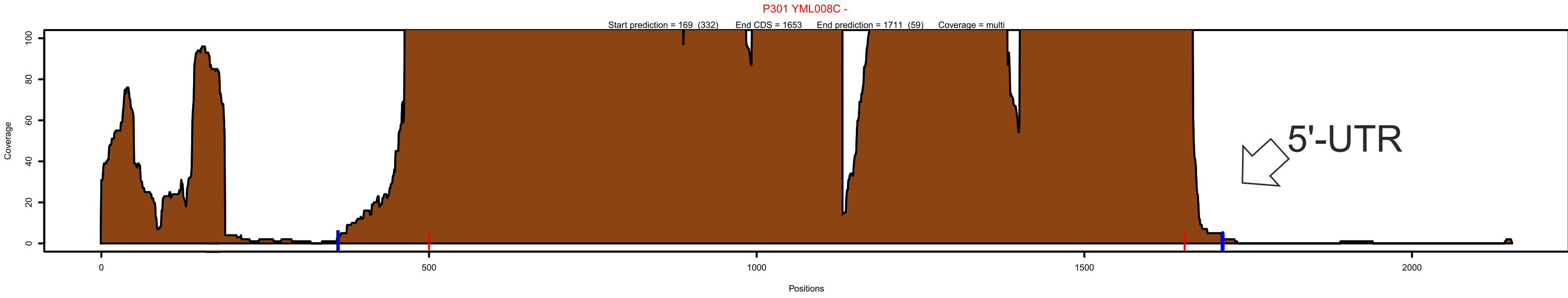

sterol metabolic process

YMR202W; *ERG2*

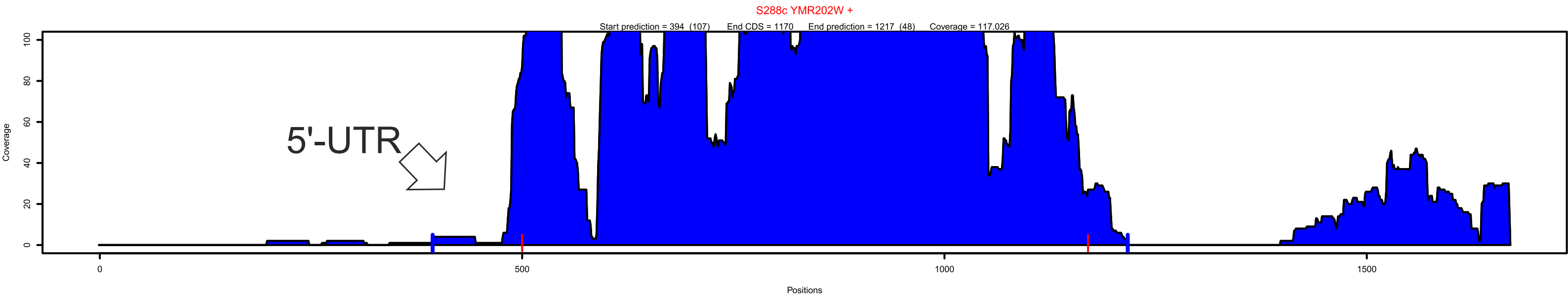

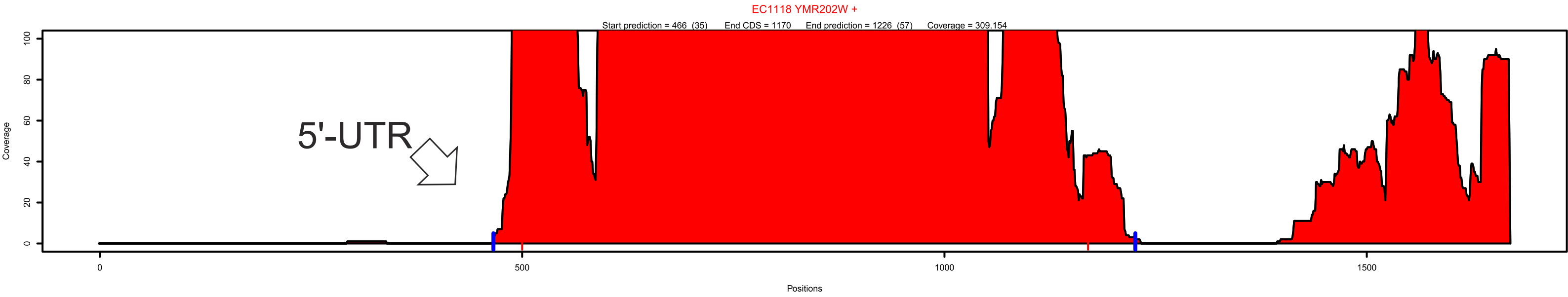

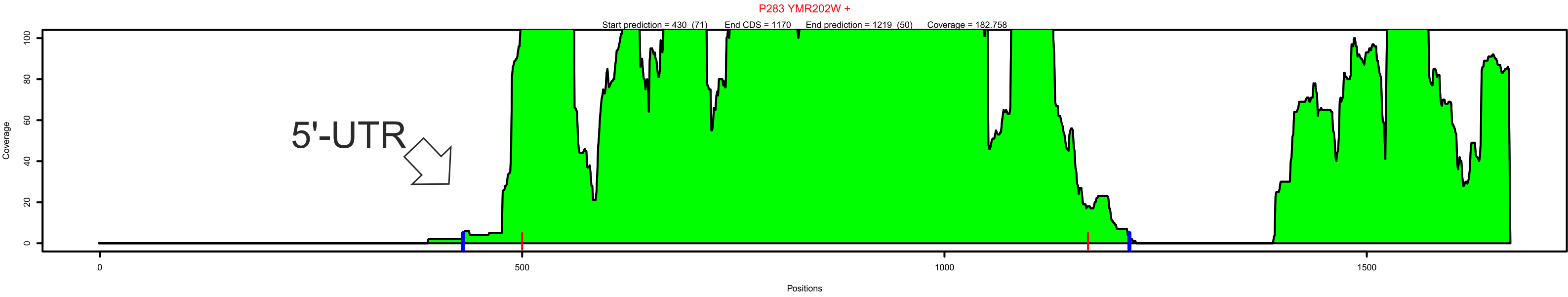

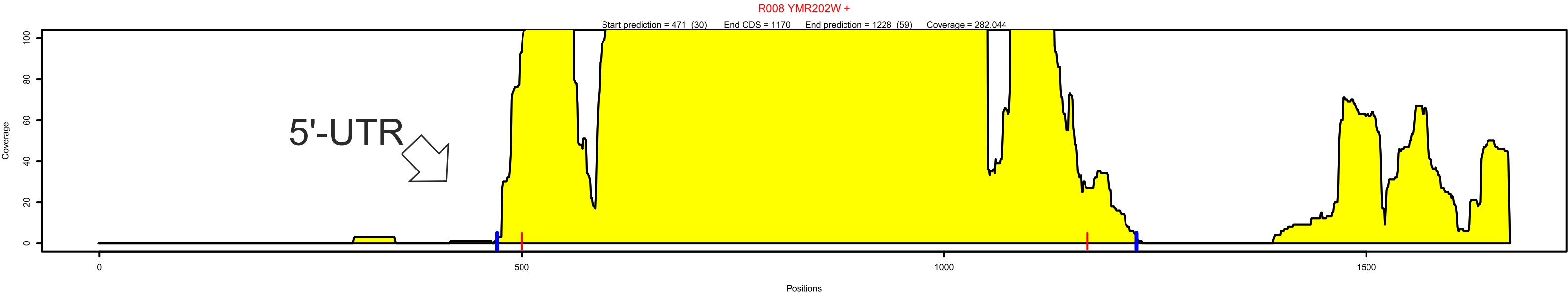

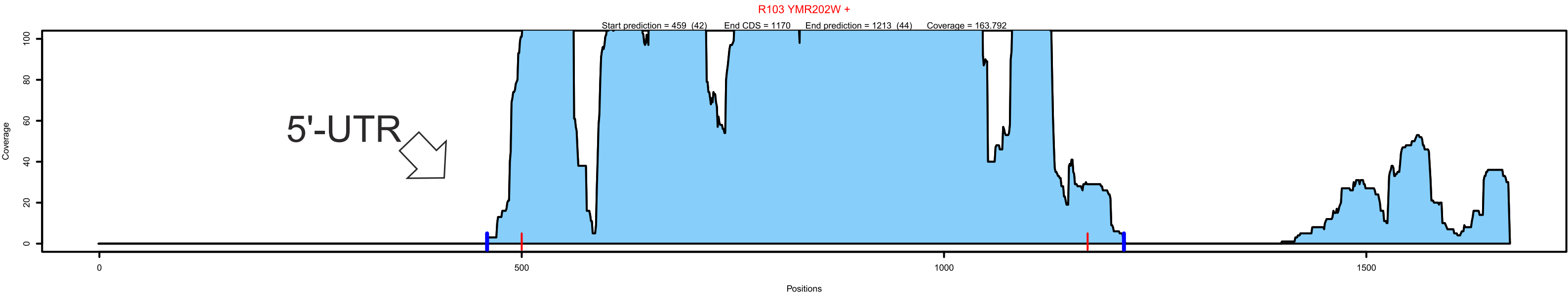

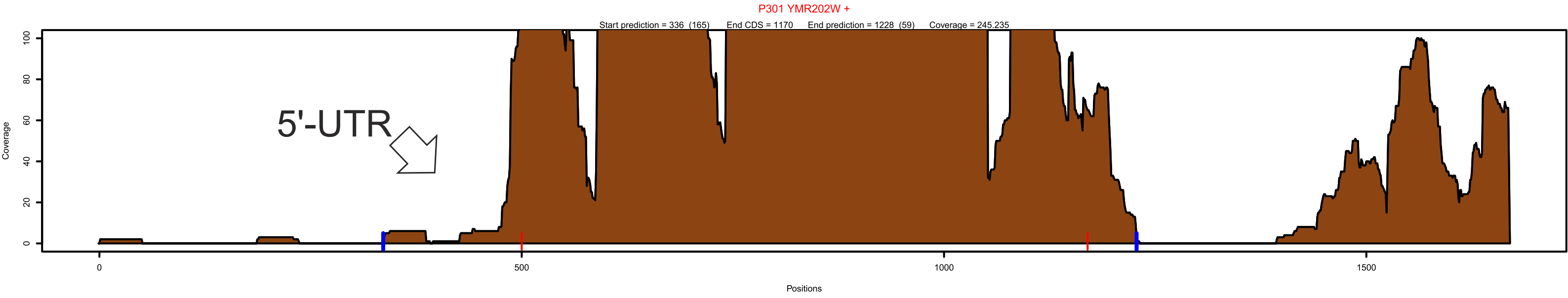

sterol metabolic process

YNL280C; *ERG24*

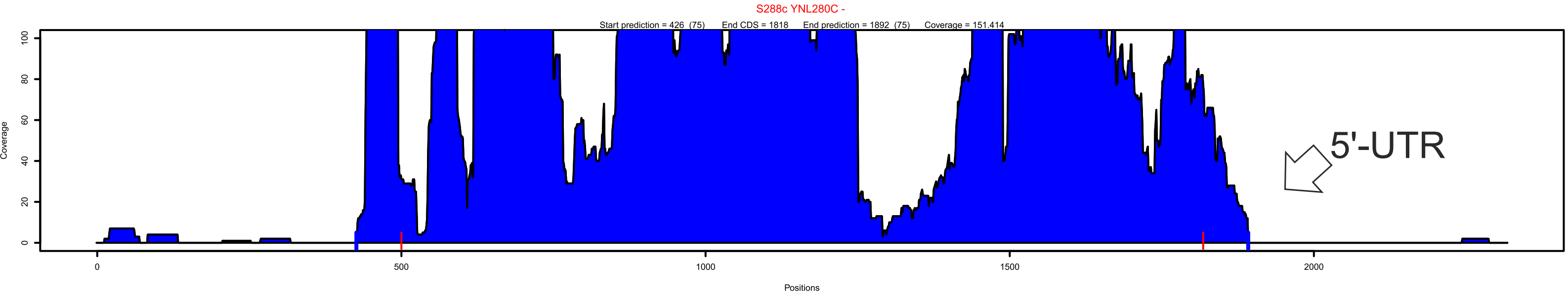

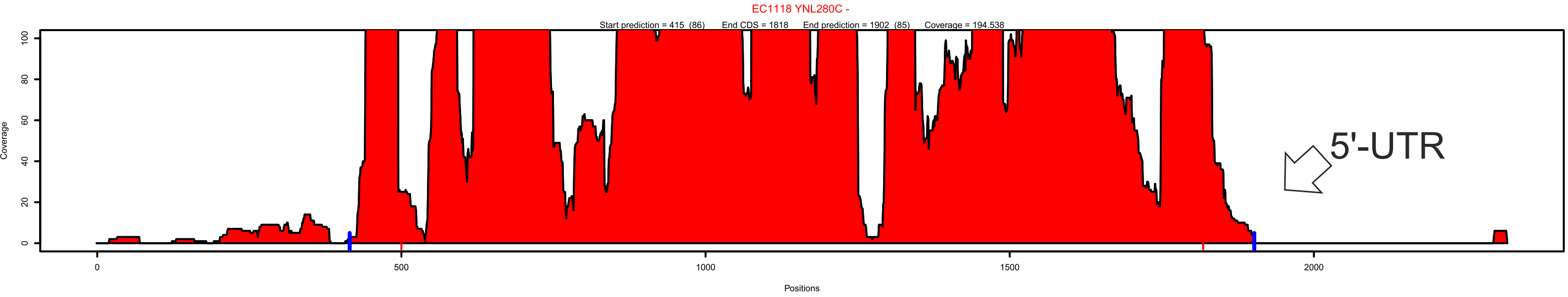

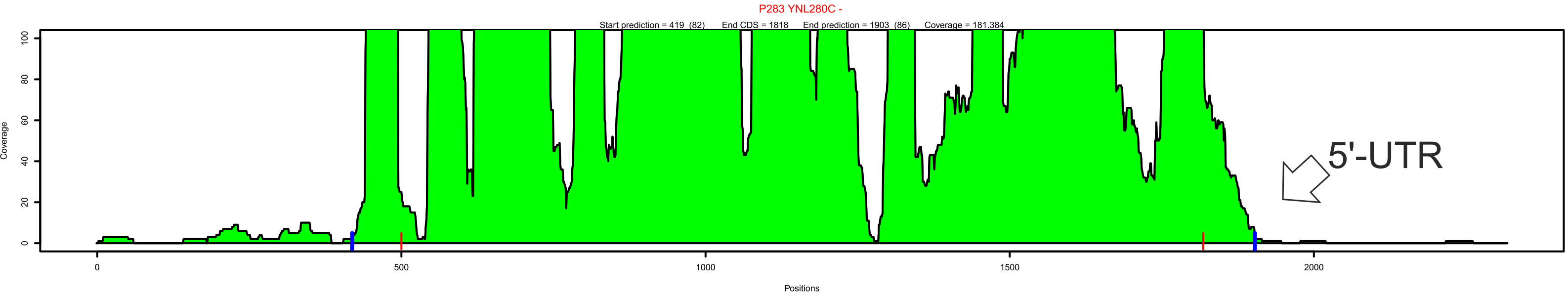

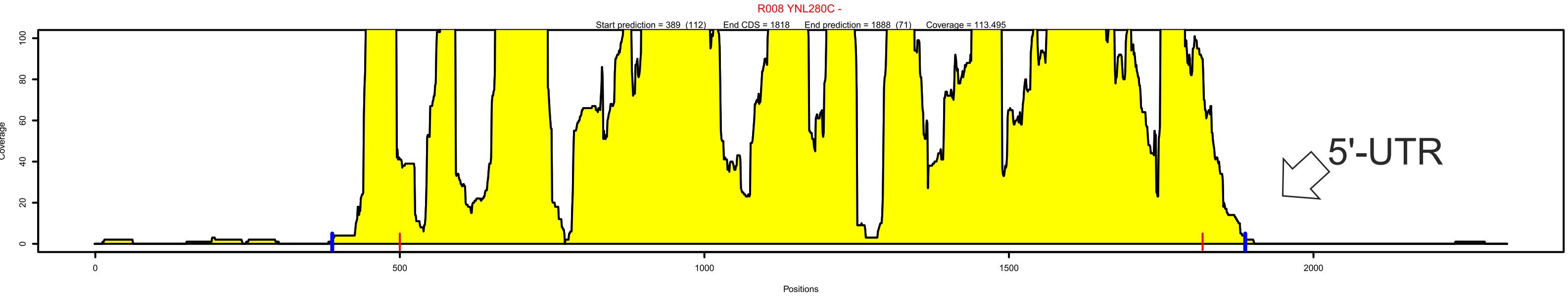

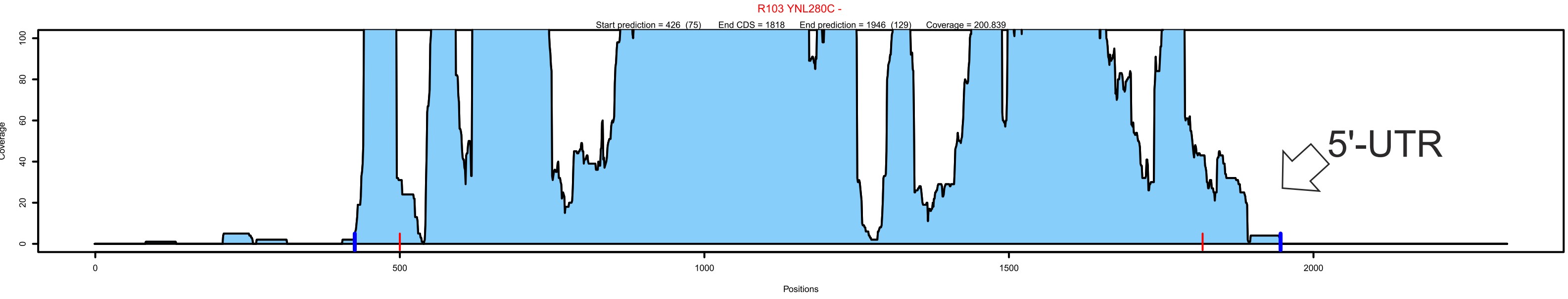

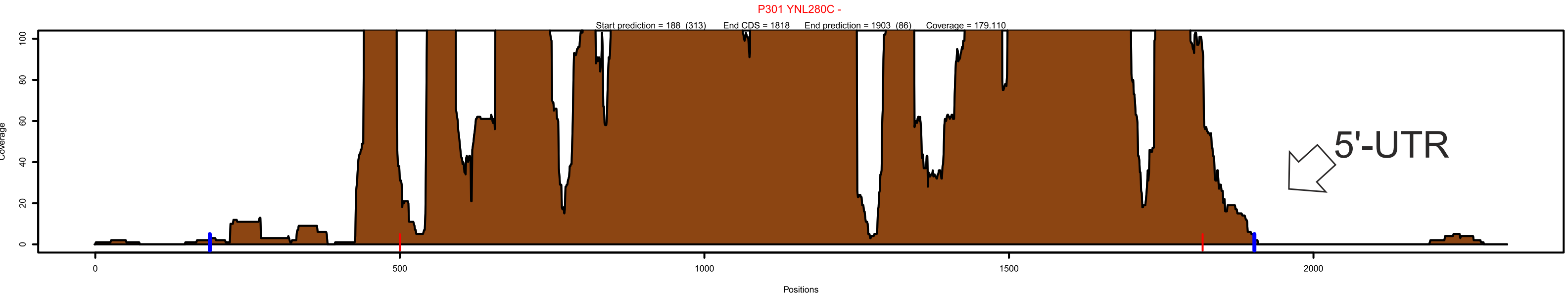

Additional file 3: Figure S2. Some selected results of the transcript assembly process obtained for the six strains under analysis. Genes described belong to the GO categories “sulfur compound metabolic process” and “sterol metabolic process” that are described in the main text. Genes YDR213W and YGL012W have highly conserved 5'-UTRs and were reported to show how gene expression level has little influence on the transcript structure prediction. The 5' transcript end is on the left for genes encoded on the forward strand and on the right for genes encoded on the reverse strand. 5'-UTR is indicated by a small arrow.
