# Supplementary material for: Transcriptome structure variability in Saccharomyces cerevisiae strains determined with a newly developed assembly software
Source: BMC Genomics. 2014 Dec 1;15(1):1045. doi: 10.1186/1471-2164-15-1045 (PMC4302112; doi:10.1186/1471-2164-15-1045)
Supplement: Supplementary file 6 — Additional file 6: Figure S3: Analysis of the 5’-UTR region of ARO4 gene. (a) To verify if the RBS in ARO4 (YBR249c) promoter was differentially represented between oenological and laboratory strains, PATMAN software [52] (https://bioinf.eva.mpg.de/patman/patman-1.2.html) was used. In Freeberg and colleagues [39] the consensus sequences for RBSs were not provided, for this reason the number of RBS in the genomes was estimated allowing up to 3 mismatches in the sequence of the RBS obtained from S288c strain. Results indicate that the RBS is only slightly over-represented in S288c. Since the genome of the enological strains is not complete, results in the table were normalized considering the length of the genomes. (b) Visual representation of the ARO4 promoter showing the predicted TATA box and the RBS. (PDF 249 KB) [file 12864_2014_6763_MOESM6_ESM.pdf]

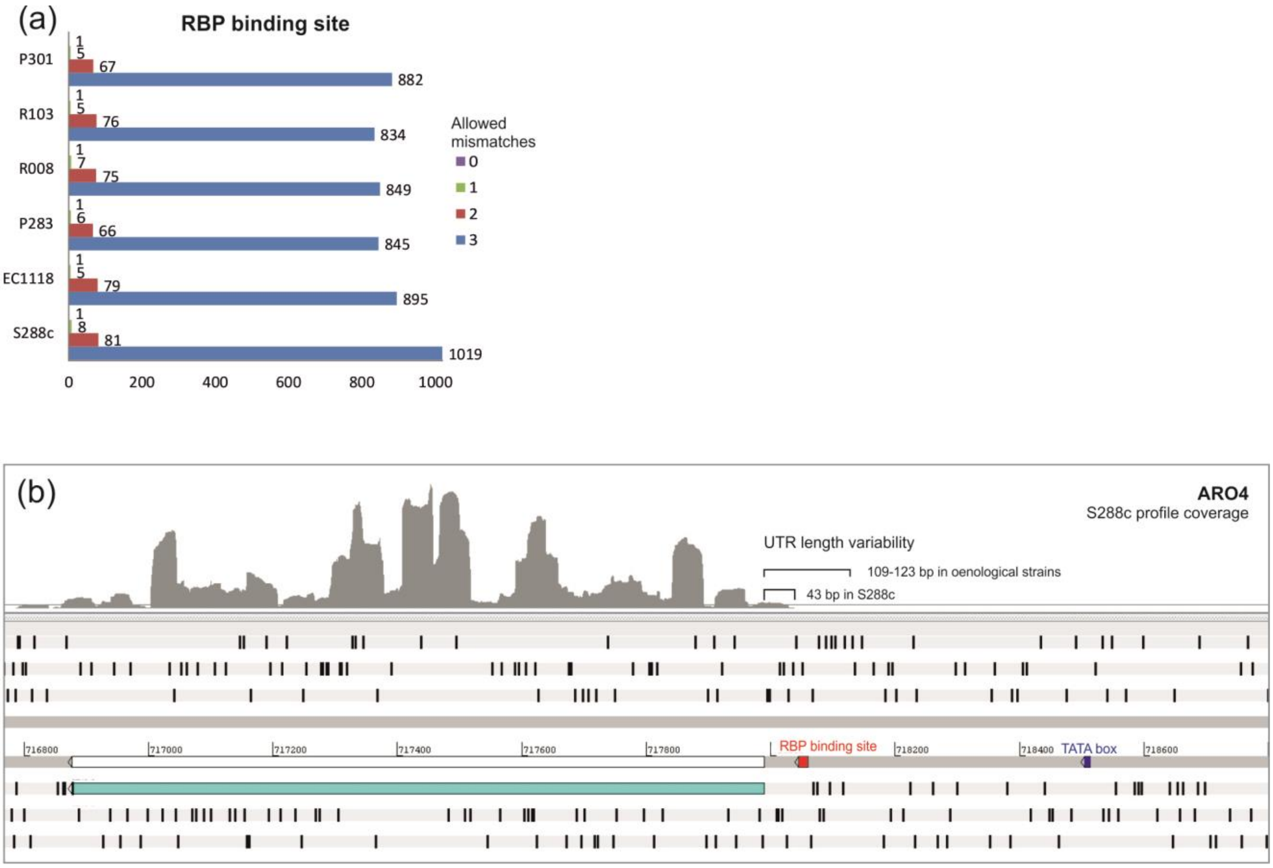

Additional file 6: Figure S3. Analysis of the 5'-UTR region of *ARO4* gene. (a) To verify if the RBS in *ARO4* (YBR249c) promoter was differentially represented between oenological and laboratory strains, PATMAN software (https://bioinf.eva.mpg.de/patman/patman-1.2.html) was used. In Freeberg and colleagues [38] the consensus sequences for RBSs were not provided, for this reason the number of RBS in the genomes was estimated allowing up to 3 mismatches in the sequence of the RBS obtained from S288c strain. Results indicate that the RBS is only slightly over-represented in S288c. Since the genome of the enological strains is not complete, results in the table were normalized considering the length of the genomes. **(b)** Visual representation of the *ARO4* promoter showing the predicted TATA box and the RBS.
