# Supplementary material for: Transcriptome structure variability in Saccharomyces cerevisiae strains determined with a newly developed assembly software
Source: BMC Genomics. 2014 Dec 1;15(1):1045. doi: 10.1186/1471-2164-15-1045 (PMC4302112; doi:10.1186/1471-2164-15-1045)
Supplement: Supplementary file 8 — Additional file 8: Figure S4: Directional reads validation. Frequency of antisense ncRNAs (named SAUTs in the paper) in highly expressed genes was determined and results are reported in (a). Frequency of genes having SAUT was calculated with respect to the coverage of the corresponding genes. Some selected images of highly expressed genes are reported (b-e) to show the high strand-specificity of the library, while two examples (f-g) are reported to show genes with high antisense transcription, SAUTs are highlighted by red boxes. (PDF 483 KB) [file 12864_2014_6763_MOESM8_ESM.pdf]

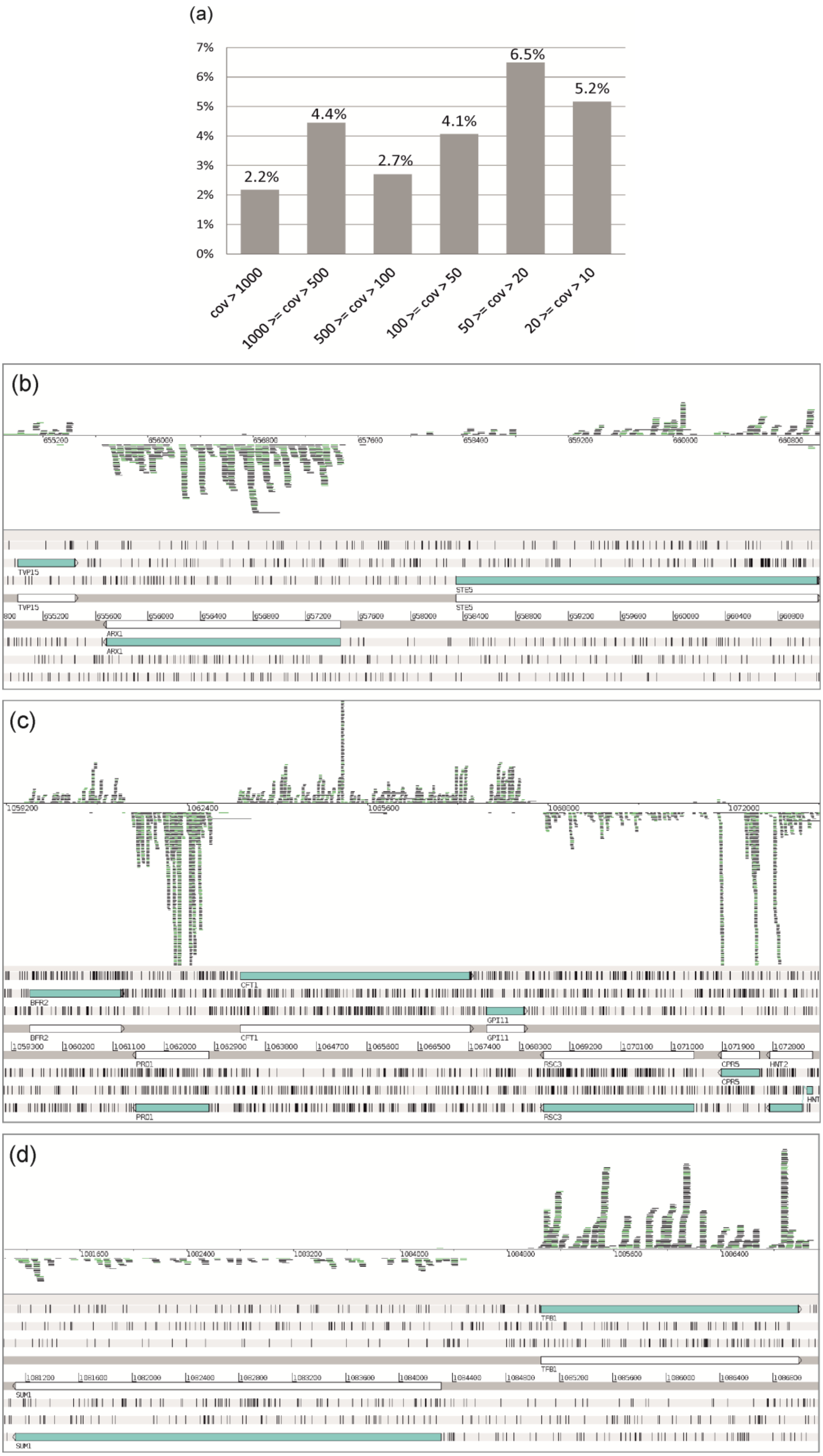

(a)
7%
6%
5%
4%
3%
2%
1%
0%
2.2%
4.4%
2.7%
4.1%
6.5%
5.2%
cov > 1000
1000 >= cov > 500
500 >= cov > 100
100 >= cov > 50
50 >= cov > 20
20 >= cov > 10
(b)
TVP15
STE5
ARX1
(c)
CFT1
BFR2
GPI11
PRO1
RSC3
CPR5
HNT2
(d)
TFB1
SUM1

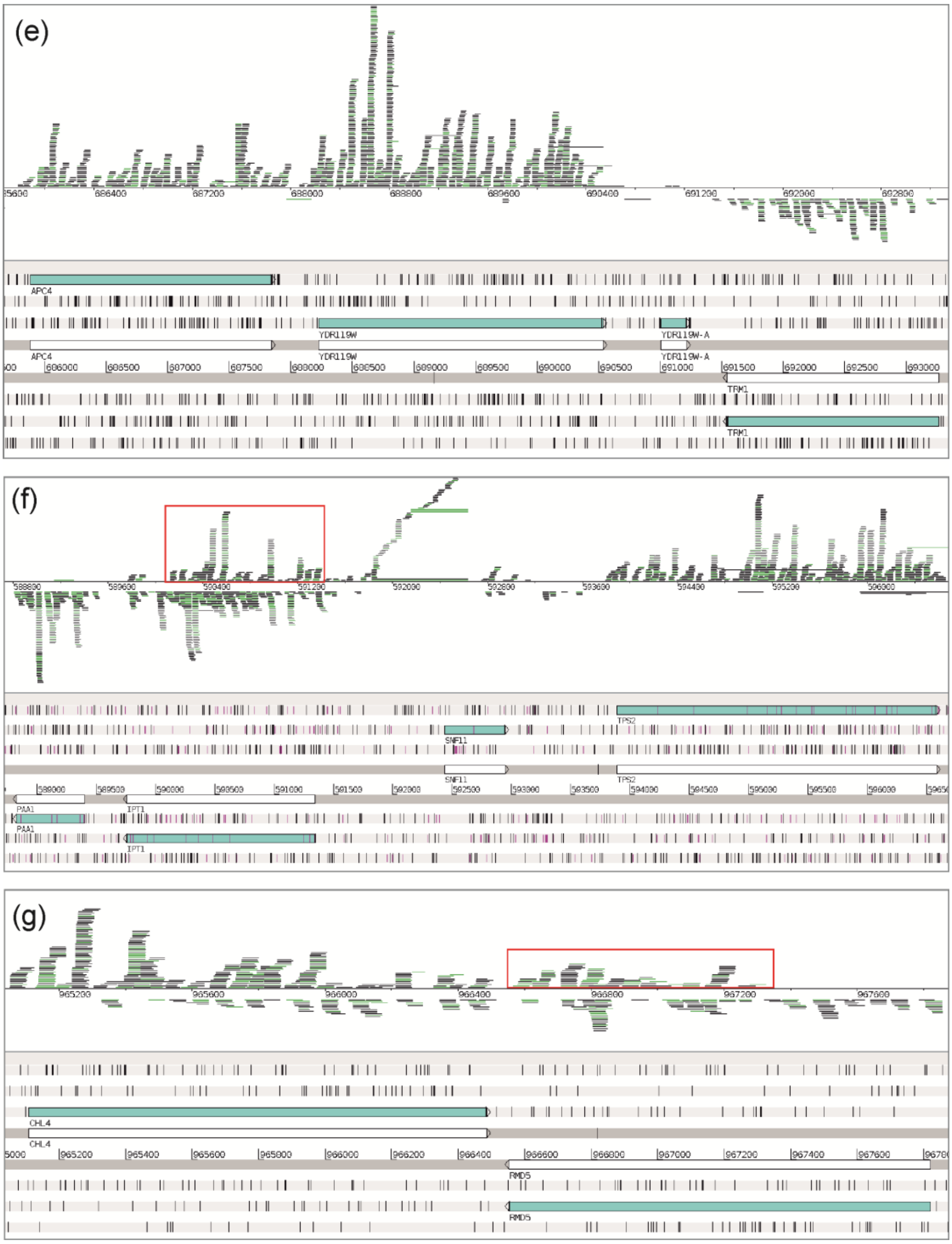

Additional file 8: Figure S4. Directional reads validation. Frequency of antisense ncRNAs (named SAUT in the paper) in highly expressed genes was determined and results are reported in **(a)**. Frequency of genes having SAUT was calculated with respect to the coverage of the corresponding genes. Some selected images of highly expressed genes are reported **(b-e)** to show the high strand-specificity of the library, while two examples **(f-g)** are reported to show genes with high antisense transcription, SAUT are highlighted by red boxes.
