# Supplementary material for: Transcriptome structure variability in Saccharomyces cerevisiae strains determined with a newly developed assembly software
Source: BMC Genomics. 2014 Dec 1;15(1):1045. doi: 10.1186/1471-2164-15-1045 (PMC4302112; doi:10.1186/1471-2164-15-1045)
Supplement: Supplementary file 9 — Additional file 9: Figure S5: Verification of bicistronic transcripts identified using ORA. Sixteen bicistronic transcripts were identified by ORA only in one of the two growth conditions (6 or 45 g/l) and were excluded from further investigation. This result highlights the importance of comparing transcriptome reconstruction in different growth conditions. The remaining three transcripts are reported in (a-c) and include three pairs of genes YFR033C-YFR034C, YFL057C-YFL056C and YIL165C-YIL164C. These transcripts were further verified considering two lines of evidence: the presence of polyadenylation sites previously determined (red boxes in the figure) [60] and the distribution of the paired-end reads obtained from a previous study (top of the figures) [56]. The first two bicistronic transcripts (a-b) are distinct transcripts not separated by untranscribed intergenic regions; this is evidenced by the high coverage differences, the paired-end distribution and/or the presence of polyadenylation sites. Only genes YIL165C-YIL164C seem to be transcribed in a real bicistronic transcript. Red arrows indicate putative transcription start sites. (PDF 369 KB) [file 12864_2014_6763_MOESM9_ESM.pdf]

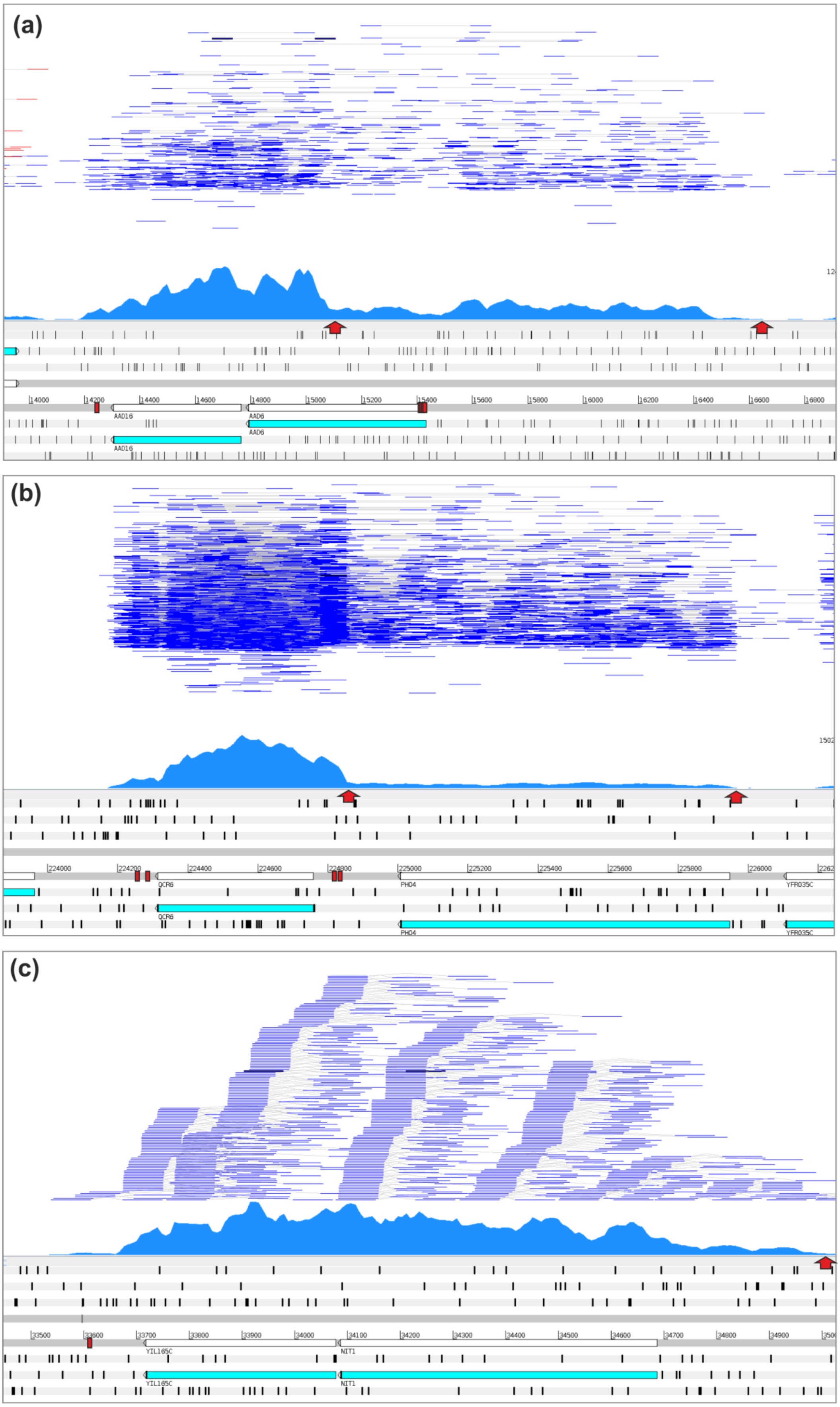
(a)
14000
14200
14400
14600
14800
15000
15200
15400
15600
15800
16000
16200
16400
16600
16800
AAD16
AAD6
AAD6
AAD16
(b)
1502
224000
224200
224400
224600
224800
225000
225200
225400
225600
225800
226000
QCR6
PHO4
YFR035C
QCR6
PHO4
YFR035C
(c)
33500
33600
33700
33800
33900
34000
34100
34200
34300
34400
34500
34600
34700
34800
34900
YIL165C
NIT1
YIL165C
NIT1

Additional file 9: Figure S5. Verification of bicistronic transcripts identified using ORA. Sixteen bicistronic transcripts were identified by ORA only in one of the two growth conditions (6 or 45 g/l) and were excluded from further investigation. This result highlights the importance of comparing transcriptome reconstruction in different growth conditions. The remaining three transcripts are reported in **(a-c)** and include three pairs of genes YFR033C-YFR034C, YFL057C-YFL056C and YIL165C-YIL164C. These transcripts were further verified considering two lines of evidence: the presence of polyadenylation sites previously determined (red boxes in the figure) [60] and the distribution of the paired-end reads obtained from a previous study (top of the figures) [56]. The first two bicistronic transcripts **(a-b)** are distinct transcripts not separated by untranscribed intergenic regions; this is evidenced by the high coverage differences, the paired-end distribution and/or the presence of polyadenylation sites. Only genes YIL165C-YIL164C seem to be transcribed in a real bicistronic transcript. Red arrows indicate putative transcription start sites.
